# Trained immunity of alveolar macrophages enhances injury resolution via KLF4-MERTK-mediated efferocytosis

**DOI:** 10.1084/jem.20221388

**Published:** 2023-08-24

**Authors:** Sreeparna Chakraborty, Abhalaxmi Singh, Li Wang, Xinge Wang, Mark A. Sanborn, Zijing Ye, Mark Maienschein-Cline, Amitabha Mukhopadhyay, Balaji B. Ganesh, Asrar B. Malik, Jalees Rehman

**Affiliations:** 1 Department of Biochemistry and Molecular Genetics, University of Illinois College of Medicine, Chicago, IL, USA; 2 Department of Pharmacology and Regenerative Medicine, University of Illinois College of Medicine, Chicago, IL, USA; 3 Division of Cardiology, Department of Medicine, University of Illinois College of Medicine, Chicago, IL, USA; 4 Department of Biomedical Engineering, University of Illinois College of Medicine, Chicago, IL, USA; 5 Research Resources Center, University of Illinois Chicago, Chicago, Illinois, USA; 6 University of Illinois Cancer Center, Chicago, IL, USA

## Abstract

Recent studies suggest that training of innate immune cells such as tissue-resident macrophages by repeated noxious stimuli can heighten host defense responses. However, it remains unclear whether trained immunity of tissue-resident macrophages also enhances injury resolution to counterbalance the heightened inflammatory responses. Here, we studied lung-resident alveolar macrophages (AMs) prechallenged with either the bacterial endotoxin or with *Pseudomonas aeruginosa* and observed that these trained AMs showed greater resilience to pathogen-induced cell death. Transcriptomic analysis and functional assays showed greater capacity of trained AMs for efferocytosis of cellular debris and injury resolution. Single-cell high-dimensional mass cytometry analysis and lineage tracing demonstrated that training induces an expansion of a MERTK^hi^Marco^hi^CD163^+^F4/80^low^ lung-resident AM subset with a proresolving phenotype. Reprogrammed AMs upregulated expression of the efferocytosis receptor MERTK mediated by the transcription factor KLF4. Adoptive transfer of these trained AMs restricted inflammatory lung injury in recipient mice exposed to lethal *P. aeruginosa*. Thus, our study has identified a subset of tissue-resident trained macrophages that prevent hyperinflammation and restore tissue homeostasis following repeated pathogen challenges.

## Teaser

Chakraborty et al. demonstrate that a subset of tissue-resident alveolar macrophages shows greater capacity for efferocytosis and prevents hyperinflammation following repeated pathogen challenges. These trained alveolar macrophages exhibit upregulation of the efferocytosis receptor MERTK mediated by the transcription factor KLF4.

## Graphical Abstract

**Figure:**
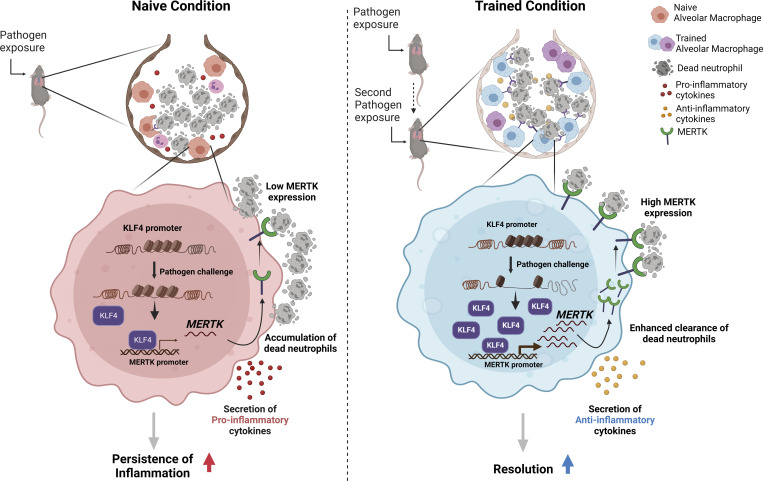

## Introduction

Immune memory as a consequence of repeated exposure to pathogens or pathogen components has been traditionally associated with the adaptive immune system and forms the basis of vaccine efficacy (Cupovic et al., 2021). Recent studies suggest that innate immune cells such as monocytes and macrophages also exhibit key features of immune memory and this is referred to as “trained immunity” (Netea et al., 2016). Most examples of trained innate immunity constitute amplified inflammatory responses in the setting of repeated pathogen exposure, thus promoting the rapid elimination of pathogens (Divangahi et al., 2021; Netea et al., 2020). Trained immunity is regulated via transcriptional, metabolic, and epigenetic reprogramming (Bekkering et al., 2018; Fanucchi et al., 2019; Lau et al., 2018) of monocytes or tissue macrophages following an initial pathogen exposure, thus triggering enhanced pathogen killing during subsequent exposures (Feuerstein et al., 2020; Kaufmann et al., 2018; Netea et al., 2020; Yao et al., 2018). However, in addition to pathogen elimination, timely and efficient resolution of inflammation is extremely important to avoid self-harm by excessively activated immune cells, thus enabling the restoration of homeostasis in the host (Medzhitov et al., 2012). However, less is known about how training of innate immune cells regulates their ability to resolve inflammation and prevent tissue injury.

Lungs are continuously exposed to environmental toxins and pathogens, thus requiring a robust and effective immune response by innate immune cells (Lambrecht, 2006). An indication of the uniqueness of lungs is that the lung endothelium (unlike other organ-specific endothelial cells) is enriched in immune cell trafficking and activation genes even during homeostasis (Jambusaria et al., 2020). This predisposition of the lung toward mounting a robust host–defense response also increases the vulnerability of the lung to inflammatory injury secondary to unchecked immune response (Lambrecht, 2006). Therefore, we studied innate immune cells of the lung to elucidate the role of trained immunity in injury resolution. Lung macrophages, alveolar macrophages (AMs), or interstitial macrophages (IMs) consist of heterogeneous subpopulations with distinct transcriptional profiles that promote inflammation or resolution of inflammation in a highly regulated and coordinated manner (Hoyer et al., 2019; Lavin et al., 2014; Misharin et al., 2017). AMs develop from fetal liver monocytes during embryonic development (Ginhoux and Guilliams, 2016; Ginhoux and Jung, 2014; van de Laar et al., 2016; Yu et al., 2017). AMs undergo cell death during exposure to pathogens (Dagvadorj et al., 2015) and have the potential to regenerate and restore tissue homeostasis (Zhu et al., 2021). In addition to the well-established host–defense function of AMs (He et al., 2017), they are critical for the resolution of inflammation and tissue integrity (Watanabe et al., 2019; Wynn and Vannella, 2016). Debris generated by cell death during inflammatory injury can itself promote immune cell activation, thus creating a feed-forward amplification of inflammation (Cai et al., 2017; Yurdagul et al., 2017). Termination of such a cycle can be brought about by efferocytosis, the phagocytosis of cellular debris by macrophages (Elliott et al., 2017), which is very important for injury resolution (Bosurgi et al., 2017). However, it remains to be determined whether training modulates the efferocytosis capacity of AMs which were previously challenged by pathogen exposure, thus promoting the resolution of inflammation during subsequent pathogen exposure and what molecular pathways underly the response.

Here, we studied the trained innate immune response in AMs using high-dimensional mass cytometry, unbiased transcriptomics, and functional studies with complementary in vivo models of inflammatory lung injury. This analysis identified a sub-population of AMs that expanded following initial exposure to either the bacterial endotoxin LPS or to live *Pseudomonas aeruginosa* (PA). Reprogrammed AMs exhibited increased activity of the transcription factor (TF) KLF4, which in other cell types has been associated with tissue repair and angiogenesis (Fan et al., 2017; Kapoor et al., 2015; Lou et al., 2020; Sangwung et al., 2017). We show that KLF4 promoted a pro-efferocytosis phenotype shift in trained AMs by upregulating the efferocytosis mediator MERTK, whereas targeted genetic deletion of KLF4 prevented the upregulation of MERTK and the pro-resolution phenotype in AMs. Importantly, reprogramming as a consequence of training was intrinsic to AMs because the adoptive transfer of trained AMs prevented severe inflammatory injury.

## Results

### Expansion of a lung macrophage subpopulation following repeat endotoxin exposure

**Figure 1.**
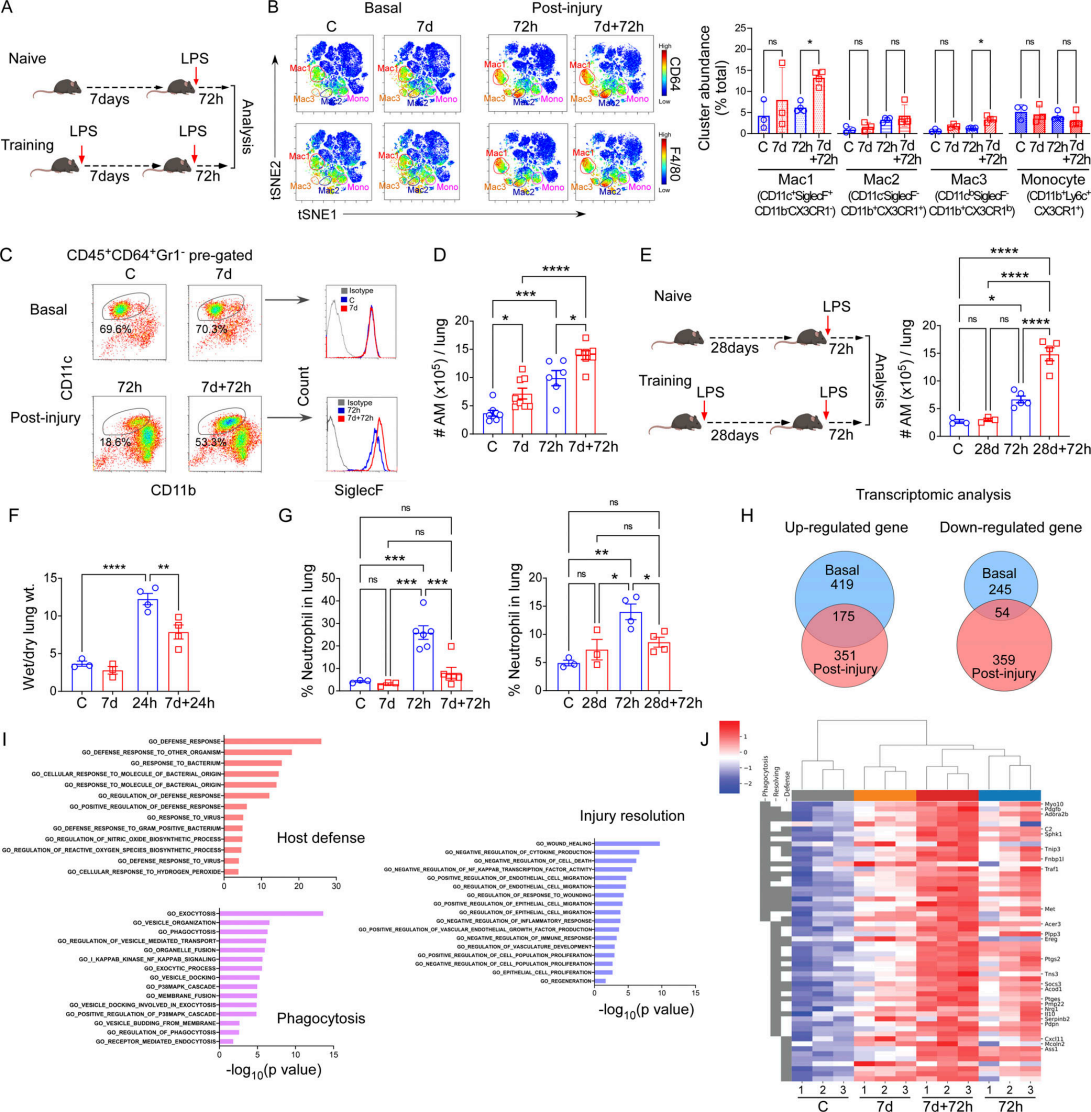
**Phenotypic characteristics of AMs generated via repeated LPS challenges. (A)** Schematic showing immune training in the inhaled LPS acute lung injury mouse model. **(B)** tSNE map derived from CyTOF analysis of lung CD45^+^ cells from naïve and trained mice at baseline (i.e., naïve baseline and 7 d-LPS after recovery baseline) and the acute 72-h LPS injury time points (i.e., 72 h and 7 d + 72 h). CD64 (upper panel) and F4/80 (lower panel) expressions are highlighted by colored gradient expression; three macrophage clusters (i.e., Mac1–3) and one monocyte cluster (i.e., mono) were manually gated (left panel). The differentially expressed markers for individual clusters are shown in the figure. In the right panel, the graph shows cluster abundance (% of total). Data were collected from four independent experiments, and the cells were combined from three mice per group with *n* = 9–12 samples and were analyzed by ANOVA with Bonferroni’s multiple comparisons test. **(C)** Flow cytometric plot shows CD45^+^CD64^+^Gr1^−^CD11c^+^CD11b^−/lo^SiglecF^+^ macrophage populations in basal conditions and 72 h after LPS in naïve and prechallenged mice. **(D)** Absolute number of CD45^+^CD64^+^Gr1^−^CD11c^+^CD11b^lo/−^SiglecF^+^ cells per lung was determined by flow cytometry. Data were collected from six independent experiments, (*n* = 6–8) and were analyzed by ANOVA with Bonferroni’s multiple comparisons test. **(E)** Schematic of long-term immune training (left panel). The right panel shows the absolute number of AM (CD45^+^CD64^+^Gr1^−^CD11c^+^CD11b^lo/−^SiglecF^+^) within the lung 72 h after LPS in naïve and long-term (28 d)-trained mice. Data were collected from three independent experiments (*n* = 3–5) and were analyzed by ANOVA with Bonferroni’s multiple comparisons test. **(F)** The graph shows the wet-to-dry lung weight ratio at the mentioned time points in naïve and trained mice. Data were collected from three independent experiments (*n* = 3–4) and were analyzed by ANOVA with Bonferroni’s multiple comparisons test. **(G)** The percentage of neutrophil (CD45^+^CD11b^+^Ly6g^+^) within CD45^+^ cells in the lung of naïve and prechallenged mice at basal and 72 h after LPS at 7-d intervals (upper panel) and 28-d intervals (lower panel) were shown. Data were collected from six independent experiments (*n* = 3–6) and were analyzed by ANOVA with Bonferroni’s multiple comparisons test. **(H)** Venn diagram represents shared upregulated and downregulated gene numbers between the homeostasis and post-LPS conditions in trained AMs as compared to naïve ones. **(I)** The enriched GO terms from upregulated genes between trained and naïve AMs in post-injury conditions. **(J)** Heatmap representation of differentially expressed genes, identified by comparing trained AMs to naïve AMs at homeostasis and 72 h after LPS challenge. The blue-to-white-to-red gradient represents the increased expression of the genes with blue representing minimal expression and red representing high expression. Data were collected from three independent experiments, and the cells were combined from two mice per group with *n* = 6 samples. Graphs show mean ± SD, with each dot representing an individual mouse data point. *P < 0.05, **P < 0.01, ***P < 0.001, ****P < 0.0001.

**Figure S1.**
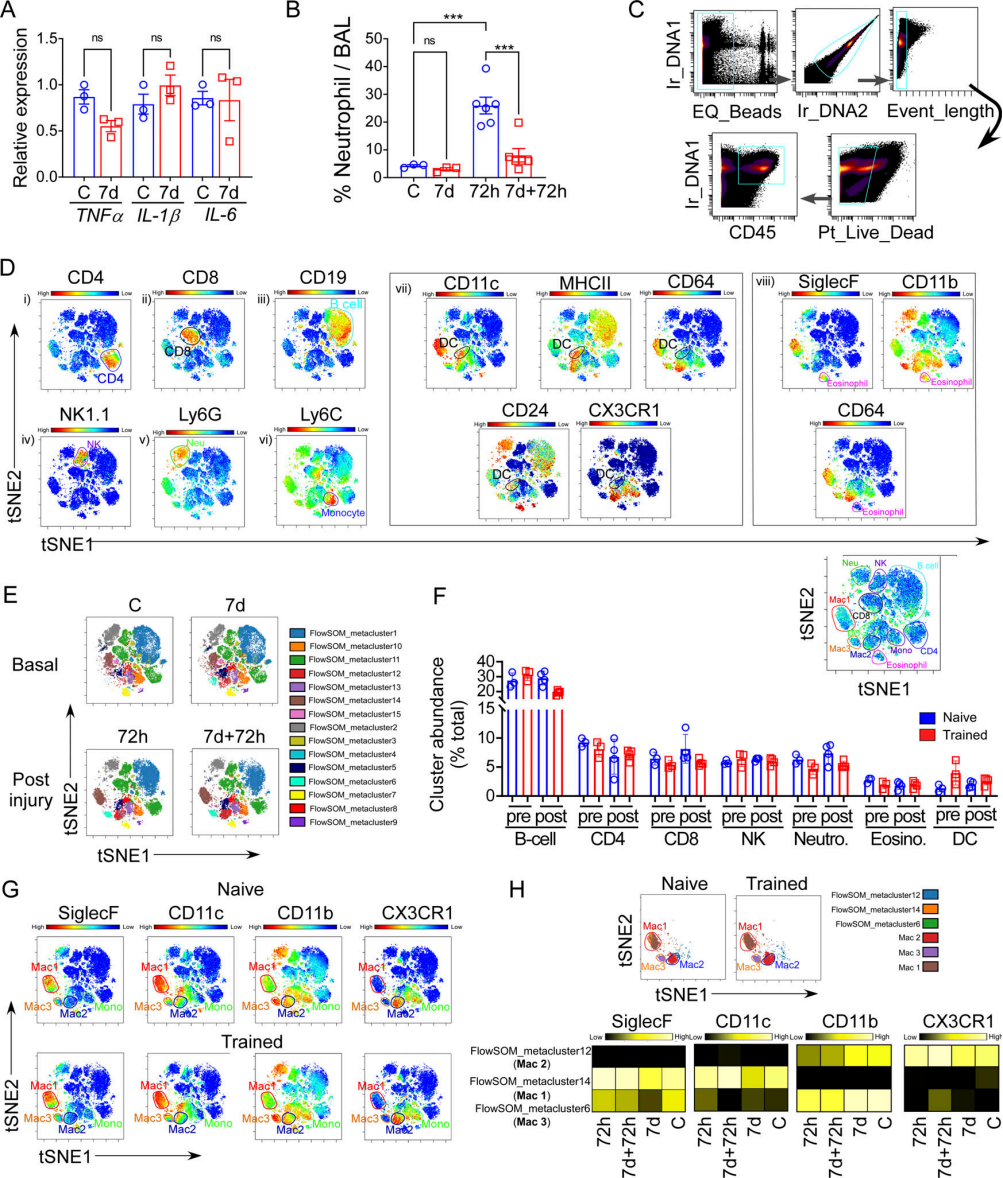
**Phenotypic characteristics of in vivo generated trained AM. (A)** The graph shows the relative mRNA expression of *TNFα*, *Il-1β*, and *IL-6* in digested lung tissue from naïve and 7 d after LPS–challenged trained mice. Data were collected from three independent experiments (*n* = 3) and were analyzed by ANOVA with Bonferroni’s multiple comparisons test. **(B)** The flow cytometry quantification of percent neutrophil in BAL in naïve and trained mice at basal (C and 7 d) and 72 h after LPS challenge (72 h, 7 d + 72 h). Data were collected from three independent experiments (*n* = 3–6) and were analyzed by ANOVA with Bonferroni’s multiple comparisons test. Graphs show mean ± SD with each dot representing an individual mouse data. ***P < 0.001. **(C)** The gating strategy for CyTOF analysis of CD45^+^ cells in mice lungs. **(D)** Representative tSNE plots were generated by mass cytometry of 50,000 lung cells isolated from naïve mice following 72 h after LPS challenge by using 17 metal-labeled antibodies to different surface markers. The colored graded expression of markers are as follows: (i) CD4; (ii) CD8; (iii) CD19; (iv) NK1.1; (v) Ly6G; (vi) Ly6c; (vii) CD11c, MHCII, CD64, CD24, CX3CR1; (viii) SiglecF, CD11b, CD64 to gate the cell population CD4, CD8, B cell, NK cell, neutrophil, monocytes, DC, and eosinophil, respectively. **(E)** tSNE plots show the metaclusters (MC) generated by the unsupervised clustering algorithm FlowSOM, from 50,000 lung cells isolated from naïve and trained mice at the basal condition and 72 h after LPS challenge by using aforementioned 17 metal-labeled antibodies. **(F)** The tSNE plot shows gated major immune subsets identified above, and the graph represents the cluster abundance (% total) of different immune cells subpopulations other than macrophages. **(G)** The tSNE plots shows the gradient expression of SiglecF, CD11c, and CD11b in gated Mac1–3 clusters of naïve and trained mice lungs at 72 h after LPS challenge. **(H)** In the upper panel, tSNE plots show the unsupervised clustering algorithm FlowSOM metaclusters overlap with Mac1–3 cluster generated by the viSNE in naïve and trained mice (72 h after LPS). In the bottom panel, heatmaps show expression of SiglecF, CD11c, CD11b, and CX3CR1 markers in the different clusters of macrophages in naïve and trained mice at basal (C and 7 d) and 72 h after LPS challenge (72 h and 7 d + 72 h). For above mentioned CyTOF experiments, the representative plots were generated with cells from *n* = 3 mice per group.

To investigate the trained immunity in the lung, we developed a model in which mice were challenged with inhaled bacterial endotoxin LPS to induce inflammatory lung injury, followed by a second similar LPS challenge 7 d later (Fig. 1 A). The proinflammatory cytokine profile of lung tissue, as well as neutrophil percentage in bronchoalveolar lavage (BAL; Fig. S1, A and B), confirmed that inflammation subsided to basal levels at 7 d following the initial challenge. This sequence of LPS challenges allowed us to study distinct injury phases: (i) the naïve baseline condition, (ii) the acute response following the initial challenge, (iii) the trained baseline condition, and (iv) the trained response to the second acute challenge given after recovery from the initial LPS challenge. We performed multidimensional time-of-flight mass cytometry (CyTOF) to identify which immune cell subpopulations showed the most prominent changes in trained mice as compared to naïve mice. Here, we used a standard gating strategy for CyTOF analysis (Fig. S1 C). For visualization, 50,000 lung cells from multiple biological replicates were projected onto a two-dimensional plane using visual t-distributed stochastic neighbor embedding (viSNE; Amir el et al., 2013) via the analysis platform Cytobank (https://www.cytobank.org; Fig. 1 B and Fig. S1 D). The use of 17 CyTOF cell identity markers identified 11 immune cell subsets; i.e., three clusters of macrophages (Mac1–3; CD45^+^CD64^+^F4/80^+^Ly6G^−^; Fig. 1 B and Fig. S1 D), (i) CD4^+^ T cells (CD45^+^CD4^+^), (ii) CD8^+^ T cells (CD45^+^CD8^+^), (iii) B cells (CD45^+^CD19^+^), (iv) NK cells (CD45^+^NK1.1^+^), (v) neutrophils (CD45^+^Ly6G^+^), (vi) monocytes (CD45^+^Ly6c^+^), (vii) dendritic cells (DCs; CD45^+^CD64^−^CD11c^+^MHCII^+^CD24^+^CX3CR1^−^), and (viii) eosinophil (CD45^+^CD64^−^CD11b^+^SiglecF^+^; Fig. 1 B and Fig. S1 D). We then used the unbiased clustering algorithm FlowSOM (Grandi et al., 2020; Van Gassen et al., 2015), which identified distinct cell clusters, thus corroborating the clusters identified by sequential gating of cell populations using established markers (Fig. S1 E). Quantification of relative cluster abundance showed that macrophage populations (Mac1–3) demonstrated the most prominent shifts with training (Fig. 1 B), whereas other immune cells were mostly stable (Fig. S1 F). To further extend the analysis, we used CD11c, SiglecF, CD11b, and CX3CR1 as established markers for distinguishing lung IM and AM populations (Guilliams et al., 2013; Janssen et al., 2011) among those three macrophage clusters (Mac1–3; Fig. S1 G). These Mac1–3 clusters corresponded to the FlowSOM metaclusters_14, 12, and 6 (Fig. S1 H) based on the differential expression of CD11c, CD11b, SiglecF, and CX3CR1 markers. Mac1 cells were primarily CD11b^−^CD11c^+^SiglecF^+^CX3CR1^−^, consistent with an AM phenotype, and Mac2 cells were predominantly CD11b^+^CD11c^−^SiglecF^−^CX3CR1^+^, consistent with an IM phenotype. Mac3 cells were CD11b^+^CD11c^lo^SiglecF^−^CX3CR1^lo^, and thus mirrored the recently described phenotype of transitional macrophages (Aran et al., 2019; Fig. S1 H).

**Figure S2.**
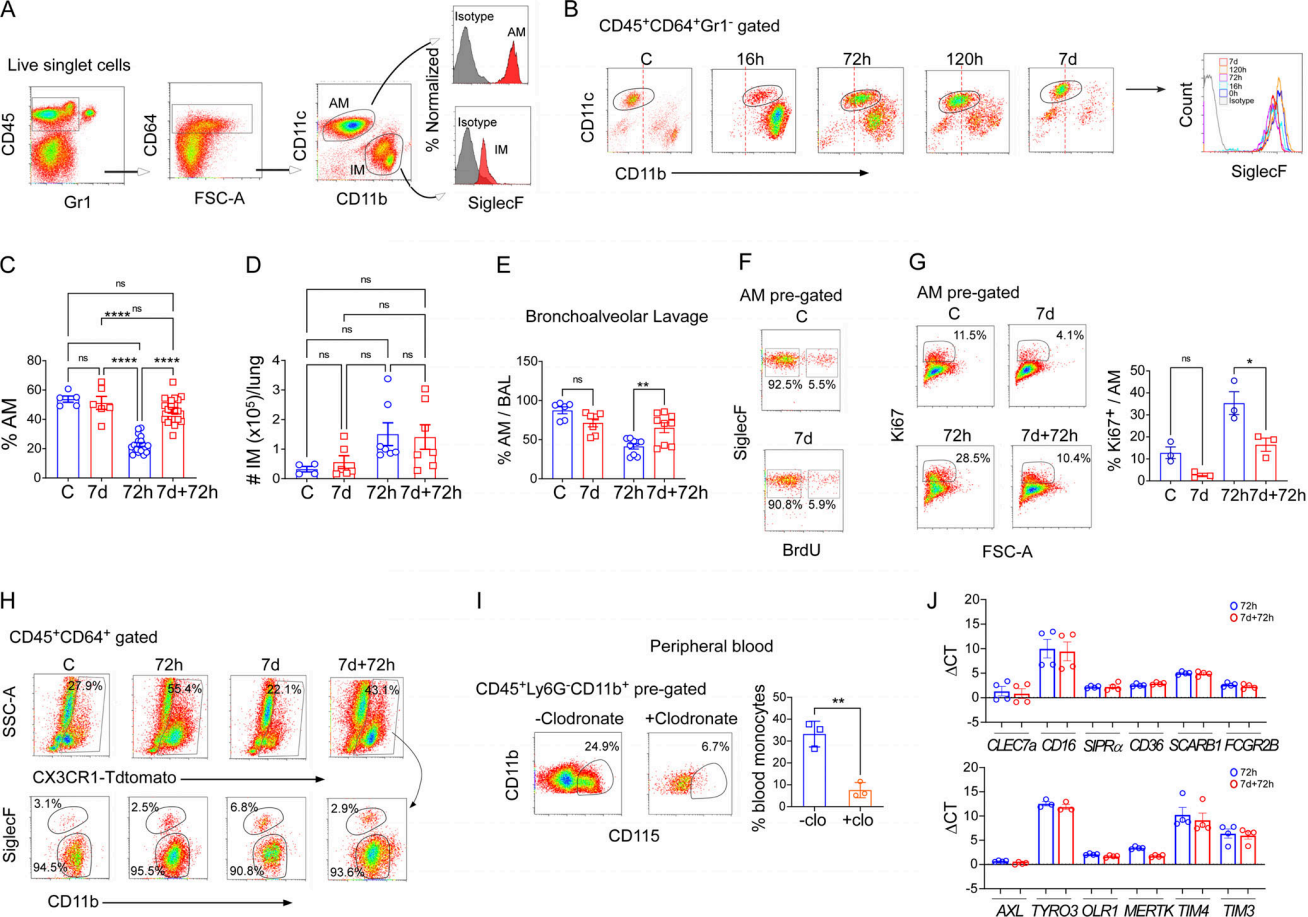
**AM dynamics in naïve vs. trained LPS challenged mice. (A)** Flow cytometry gating strategy for analysis of lung macrophages. **(B)** The representative flow cytometry plots show the dynamic changes in the phenotypic profile of AM at different stages of injury. Red lines mark the position of CD11c^+^CD11b^−^ naïve AM. In the right panel, the histogram overlay shows the SiglecF expression within CD45^+^CD64^+^Gr1^−^CD11b^lo^CD11c^+^ gated population in the above condition. **(C)** The graph represents the percentage of AMs (CD45^+^CD64^+^Gr1^−^CD11c^+^CD11b^lo/−^SiglecF^+^) within the total lung macrophage (CD45^+^CD64^+^Gr1^−^ cells pre-gated) population at basal and different stages of after LPS challenge. Data were collected from eight independent experiments (*n* = 5–20) and were analyzed by ANOVA with Bonferroni’s multiple comparisons test. **(D)** The graph shows the absolute numbers of IMs (CD45^+^CD64^+^Gr1^−^CD11c^−^CD11b^+^SiglecF^−^) in naïve and trained mice at basal (C and 7 d) and 72 h after LPS challenge condition (72 h, 7 d + 72 h). Data were collected from six independent experiments (*n* = 4–7) and were analyzed by ANOVA with Bonferroni’s multiple comparisons test. **(E)** The percentage of AM in BAL was analyzed by flow cytometry. Data were collected from six independent experiments (*n* = 7–9) and were analyzed by ANOVA with Bonferroni’s multiple comparisons test. **(F)** The flow cytometry plots show the BrdU positivity of naïve and trained AMs (CD45^+^CD64^+^CD11b^−/lo^CD11c^+^SiglecF^+^) at basal (C and 7 d) conditions. **(G)** The Ki67 positivity of naïve and trained AMs at basal and 72 h after LPS was analyzed and quantified by flow cytometry. Data were collected from three independent experiments (*n* = 3) and were analyzed by ANOVA with Bonferroni’s multiple comparisons test. **(H)** In the upper panel, representative flow cytometry plots show the percent CX3CR1 positivity within lung macrophages (CD45^+^CD64^+^ pre-gated) cells. Proportion of IM (CD11b^+^SiglecF^−/lo^) and AM (CD11b^−^SiglecF^+^) within the CX3CR1-tdTomato^+^ cells was shown in the bottom panel. **(I)** The flow cytometry dot plot shows circulating monocytes (CD115^+^CD11b^+^) within the CD45^+^Ly6G^−^CD11b^+^ pre-gated population in untreated and i.v. clodronate liposome injected mice (left panel) and analyzed (right panel). Data were collected from three independent experiments (*n* = 3) and were analyzed by unpaired *t* test. **(J)** The graphs show the delta CT values of the respective mRNA expression in naïve and trained AMs to show overall mRNA expression levels. *PPAI *(peptidylprolyl isomerase A) has been used as the internal control in qPCR. Data were collected from three independent experiments (*n* = 3–4). Graphs show mean ± SD with each dot representing an individual mouse data. *P < 0.05, **P < 0.01, ****P < 0.0001.

Next, we decided to further characterize the trained innate response in LPS-challenged AM by comparing the AM population dynamics between naïve and trained mice (gating strategy: Fig. S2 A). In naïve mice, we observed pronounced depletion of AMs (CD45^+^CD64^+^Gr1^−^CD11c^+^CD11b^−/+^SiglecF^+^) as early as 16 h after LPS, with the gradual replenishment to basal levels after 7 d (Fig. S2 B). The depletion and subsequent replenishment of AMs demonstrated a transient shift in the phenotype as the AMs expressed adhesion molecule CD11b at 16 h (CD11c^+^CD11b^+^SiglecF^+^), which was downregulated by 120 h such that AMs returned to the CD11b-negative state similar to naïve AMs (CD11c^+^CD11b^−^SiglecF^+^) at 7 d after LPS challenge (Fig. S2 B).

However, when we compared the dynamics of AMs (CD45^+^CD64^+^Gr1^−^CD11c^+^CD11b^−/+^) between the naïve and trained mice group (Fig. 1, C and D), we observed that the percentage of AMs was higher in trained mice (LPS challenge followed by 1-wk rest and subsequent second LPS challenge) at 72 h after LPS challenge as compared with naïve mice (subjected to single LPS challenge; Fig. 1 C and Fig. S2 C). To assess whether the training-induced increase in AMs was due to a shift in relative percentages or an absolute increase in AMs, we examined AM numbers per lung and observed significantly higher AM absolute numbers per lung in trained mice (Fig. 1 D). We also assessed the lung IM number and observed that, unlike the AMs, there was no significant difference in the lung IM numbers between trained and naïve mice (Fig. S2 D). A key feature of AMs is their recruitment into the alveolar space where they serve essential host–defense and tissue repair functions. By analyzing the percentage of AMs in BAL, we observed that the AM percentage in the alveolar space is markedly higher in trained mice as compared with naïve ones (Fig. S2 E).

We next investigated the robustness of the observed trained response of AMs by extending the time interval between the initial and the subsequent LPS challenge. Therefore, after the first LPS challenge, mice were allowed to recover for 4 wk prior to the second challenge (Fig. 1 E, left panel). Consistent with our previous result, at 72 h after LPS challenge, the absolute AM number was significantly greater in the trained mice when compared with naïve ones (Fig. 1 E, right panel). These findings indicate that the trained innate response in AMs in which sequential LPS challenges were separated by 4 wk showed similar training responses as those seen when the challenges were separated only by 1 wk.

Next, we compared lung edema between naïve and trained mice at 24 h after LPS challenge and the results showed that lung edema was significantly lower in trained mice, which suggested less injury following the subsequent endotoxin challenge (Fig. 1 F). We further assessed the neutrophil percentage at 72 h after LPS challenge to evaluate the resolution of injury, as a decrease in neutrophil accumulation is a hallmark of resolution. Interestingly, we observed that with both training intervals (7 or 28 d), the neutrophil percentage following the second challenge was significantly lower when compared with the exposure of naïve mice to the acute challenge (Fig. 1 G), thus indicating accelerated injury resolution. We next investigated the transcriptomic differences between naïve AM and trained AM. Using unbiased RNA sequencing (RNA-seq) analysis, we identified 594 upregulated genes and 299 downregulated genes in trained AMs when compared with naïve AMs at basal condition (Fig. 1 H). We also compared the transcriptomic profiles of trained versus naïve AMs exposed to an acute LPS challenge (72 h after acute LPS in both cases) and identified 526 upregulated and 413 downregulated genes in trained AMs (Fig. 1 H). We found that 175 upregulated genes and 54 downregulated genes in trained AMs were shared between basal and after injury conditions (Fig. 1 H), and these genes might be part of a core trained AM gene expression signature. To identify the underlying biological processes characterizing the trained AMs, we performed Gene Ontology (GO) pathway analysis, which showed that several GO terms associated with host defense such as “defense response,” “response to bacterium,” and “defense response to virus” were overrepresented in upregulated genes in trained AMs (Fig. 1 I). Interestingly, we also found that GO terms associated with phagocytosis and endocytosis (i.e., “regulation of phagocytosis,” “receptor-mediated endocytosis,” and “vesicle docking”), as well as injury resolution (i.e., “wound healing,” “negative regulation of cell death,” “regeneration,” and “negative regulation of inflammatory response”) were profoundly upregulated with training (Fig. 1 I). Examples of the genes upregulated by training included *IL-10* (Zhang et al., 2019b), *Socs3* (Yin and Heit, 2021), *C2* (Mehrotra and Ravichandran, 2022), and *Ptges* (Ampomah et al., 2022), which are associated with injury resolution or phagocytosis pathways and promote the phagocytosis of cellular debris, which is referred to as efferocytosis (Fig. 1 J and Table S4).

### Trained tissue-resident AMs show increased resilience following inflammatory challenges

We next addressed mechanisms underlying increased AM numbers in trained mice. We surmised that the potential underlying causes could be either (i) increased proliferation of surviving AMs after initial insult that rapidly replenished the trained AM pool, (ii) increased influx of circulating monocytes into lungs that could give rise to AMs, or (iii) resilience of trained AMs compared to naïve AMs thus blunting AM depletion in response to the second LPS insult.

**Figure 2.**
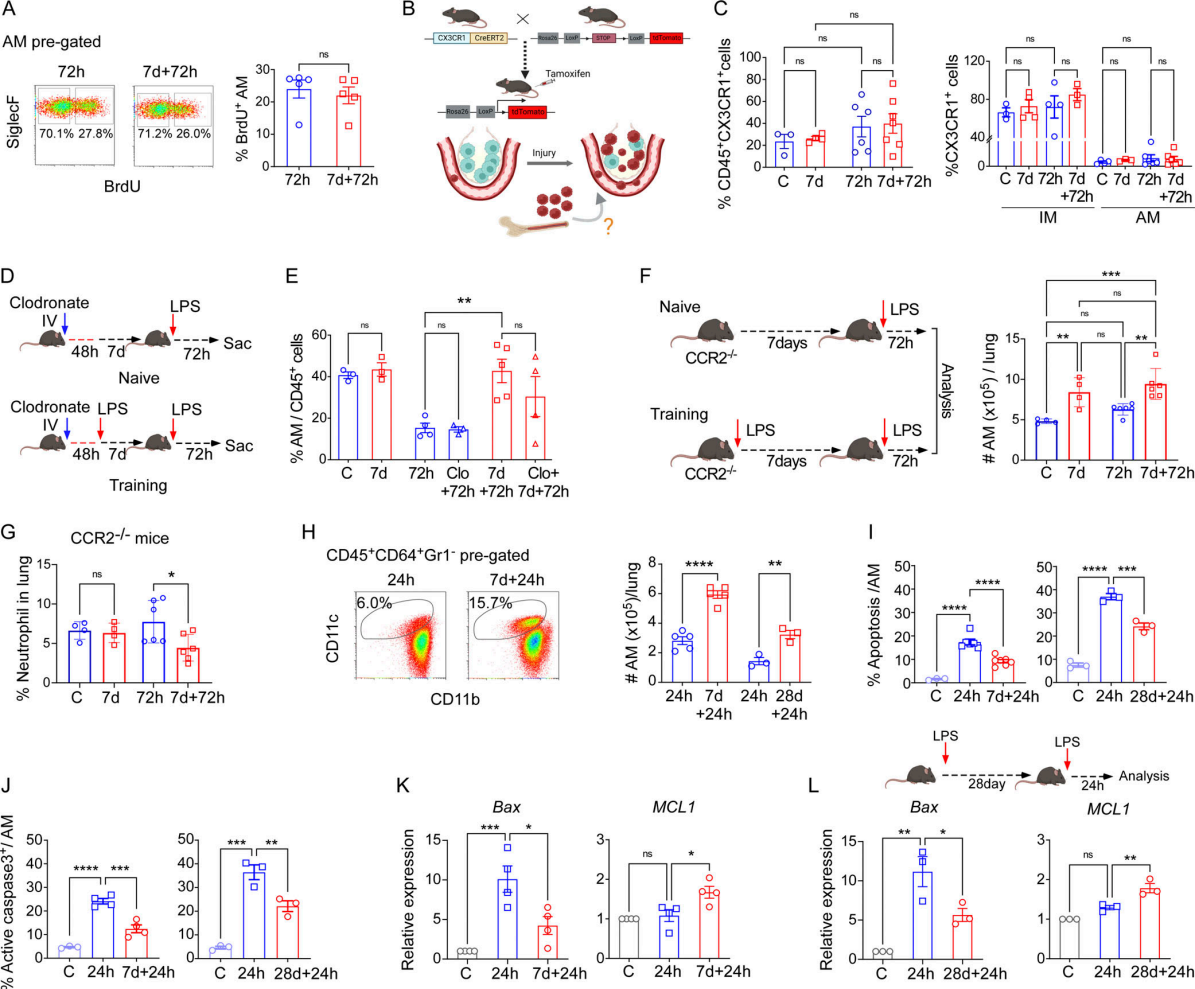
**Increased resilience of trained tissue-resident AMs following repeated inflammatory challenges. (A)** Flow cytometry plot shows AM proliferation by quantifying BrdU uptake within the AM population (CD45^+^CD64^+^CD11c^+^CD11b^lo/−^SiglecF^+^ gated) in naïve and trained mice 72 h after LPS (left panel). The right panel shows quantification of percent BrdU-positive AM within all lung macrophages (CD45^+^CD64^+^ gated). Data were collected from four independent experiments (*n* = 5) and analyzed by unpaired *t* test. **(B)** Schematic representation of lineage-tracing monocyte-derived AM after injury. **(C)** Graph in the left panel shows the percentage of total CX3CR1^+^ cells within CD45^+^ gated myeloid cells in naïve and prechallenged mice lungs at baseline condition and 72 h after LPS. In the right panel, the percent CX3CR1 positivity among CD45^+^CD64^+^CD11b^+^SiglecF^−^ (IM) and CD45^+^CD64^+^CD11b^−/lo^SiglecF^+^ (AM) gated populations was determined by flow cytometry. Data were collected from five independent experiments (*n* = 3–7) and were analyzed by ANOVA with Bonferroni’s multiple comparisons test. **(D)** Schematic representation of clodronate liposome delivery in naïve and prechallenged mice. **(E)** Graph shows percentage of AM within all lung macrophages (CD45^+^CD64^+^Gr1^−^pre-gated) in i.v. clodronate untreated or treated mice. Data were collected from three independent experiments (*n* = 3–5) and were analyzed by ANOVA with Bonferroni’s multiple comparisons test. **(F)** The schematic shows the training of CCR2^−/−^ mice by successive LPS challenges (left panel). In the right panel, the absolute number of AMs (CD45^+^CD64^+^Gr1^−^CD11b^−/lo^SiglecF^+^) was determined. Data were collected from four independent experiments (*n* = 4–6) and were analyzed by ANOVA with Bonferroni’s multiple comparisons test. **(G)** The percentage of neutrophils in CCR2^−/−^ mice in basal conditions as well as 72 h after LPS challenge was determined. Data were collected from four independent experiments (*n* = 4–6) and were analyzed by ANOVA with Bonferroni’s multiple comparisons test. **(H)** Flow cytometric plot shows CD45^+^CD64^+^Gr1^−^CD11c^−^CD11b^+/lo^SiglecF^+^ macrophage populations at 24 h after LPS in naïve and prechallenged mice (left panel). Absolute number of CD45^+^CD64^+^Gr1^−^CD11c^+^CD11b^lo/−^SiglecF^+^ cells per lung (7 and 28 d interval training) was determined (right panel). Data were collected from six independent experiments (*n* = 3–5). Data were collected from four independent experiments (*n* = 4–6) and were analyzed by ANOVA with Bonferroni’s multiple comparisons test. **(I)** Graph shows percentage of annexin V positivity within AM (CD45^+^CD64^+^Gr1^−^CD11c^+^CD11b^lo/−^SiglecF^+^ gated) at naïve and 24 h after LPS in naïve and trained mice. Data were collected from six independent experiments (*n* = 3–7) and were analyzed by ANOVA with Bonferroni’s multiple comparisons test. **(J)** The percentages of active caspase-3 positivity within gated AMs in the above-mentioned conditions were determined by flow cytometry. Data were collected from three independent experiments (*n* = 3–4) and were analyzed by ANOVA with Bonferroni’s multiple comparisons test. **(K)** Relative mRNA expression of pro-apoptotic (*Bax*) and anti-apoptotic (*MCL1*) genes in flow-sorted naïve, untrained, and trained AMs at 24 h after LPS injury was analyzed by qPCR. Data were collected from three independent experiments (*n* = 3–4) and were analyzed by ANOVA with Bonferroni’s multiple comparisons test. **(L)** Schematic of the long-term training model (upper panel). Relative mRNA expression of *Bax* and *MCL1* in naïve, untrained, and long-term (28 d) trained AMs at 24 h after second LPS challenge is shown. *PPIA* (peptidylprolyl isomerase A) was used as the internal control in qPCR. Data were collected from three independent experiments (*n* = 3) and were analyzed by ANOVA with Bonferroni’s multiple comparisons test. Graphs show mean ± SD, with each dot representing an individual mouse data. *P < 0.05, **P < 0.01, ***P < 0.001, ****P < 0.0001.

We used incorporation of the nucleotide analog BrdU in vivo to quantify AM proliferation and, as expected, we observed no AM proliferation in the basal condition of naïve mice or trained mice (Fig. S2 F). Upon LPS challenge, the proliferation of surviving AMs increased to compensate for the LPS-induced loss of AMs but there was no difference in post-LPS AM proliferation rates when comparing naïve versus trained mice, suggesting that the increased AM number in trained mice was not due to increased AM proliferation (Fig. 2 A). Quantification of AM proliferation was independently validated through the proliferation marker Ki67 (Fig. S2 G).

To study the fraction of AMs derived from circulating monocytes, we used CX3CR1-Cre^ERT2^/Rosa mice for genetic lineage tracing of circulating CX3CR1^+^ monocyte progeny (Aran et al., 2019; Yona et al., 2013; Fig. 2 B). Although we observed a trend toward an increased percentage of CX3CR1-lineage cells in the lungs at 72 h after LPS when compared to the naïve condition, it was not statistically significant (Fig. 2 C, left panel; and Fig. S2 H). Moreover, the vast majority (70–80%) of CX3CR1-derived cells in the lungs were IMs, whereas only 4% were AMs (Fig. 2 C, right panel; and Fig. S2 H). Importantly, there was no increase in CX3CR1-derived AMs in trained mice as compared with naïve mice (Fig. 2 C, right panel), indicating that the higher AM numbers observed in trained mice were not the result of increased circulating CX3CR1^+^ monocytes differentiation into AMs.

As CX3CR1-genetic lineage labeling of monocytes may not label all circulating monocyte populations (Aegerter et al., 2020), we employed a parallel approach to detect the contribution of circulating monocytes to generate the AM pool during repeat LPS insults. I.v. injection of clodronate-liposomes was used to deplete circulating monocytes (Li et al., 2016), and AM numbers were monitored at 72 h after LPS in naïve and trained mice (Fig. 2 D). Depleting circulating blood monocytes (Fig. S2 I) did not alter the greater number of AMs in trained mice (Fig. 2 E), corroborating the lineage tracing data. To evaluate the potential role of circulating monocytes in contributing to the trained AM population, we used the genetic model of CCR2^−/−^ mice in which circulating monocytes do not enter tissues due to the absence of the monocyte chemokine receptor CCR2 (Boring et al., 1997). We challenged these mice with an initial inhaled LPS exposure, followed by a second LPS challenge 7 d (Fig. 2 F, left panel). We observed that AM numbers increased with training in the absence of circulating monocytes (Fig. 2 F, right panel). Consistent with the preserved AM augmentation, we observed in CCR2^−/−^ mice that the neutrophil influx at 72 h after injury was significantly lower in trained mice (Fig. 2 G), thus confirming that circulating monocytes were not required for the training-induced enhancement of lung injury resolution.

As the results showed that increased AMs in the training setting were due to neither increased monocyte-to-AM differentiation nor increased proliferation of AMs, we posited that trained AMs likely exhibited greater resilience to LPS-induced depletion such as AM apoptosis (Dagvadorj et al., 2015). We therefore quantified the AM absolute number per lung at the early phase of injury, i.e., 24 h after LPS, and observed a significantly greater number of AMs in trained mice (Fig. 2 H). Quantitative analysis of AM apoptosis by Annexin V staining demonstrated markedly less LPS-induced apoptosis of trained AMs as compared with naïve AMs both at 7 and 28 d interval training models (Fig. 2 I). We then assessed the protein levels of active caspase-3 in AMs by flow cytometry and observed reduced apoptosis in trained mice at 24 h after LPS challenge (Fig. 2 J). In response to LPS, trained AMs showed downregulation of pro-apoptotic gene *Bax*, and upregulation of antiapoptotic gene *MCL1* at 24 h after LPS as compared with the response of naïve AMs to in vivo LPS exposure (Fig. 2, K and L). These results thus showed that training enhances AM resilience to LPS-induced apoptosis, thus increasing the AM pool.

### Trained AMs exhibit enhanced efferocytosis

**Figure 3.**
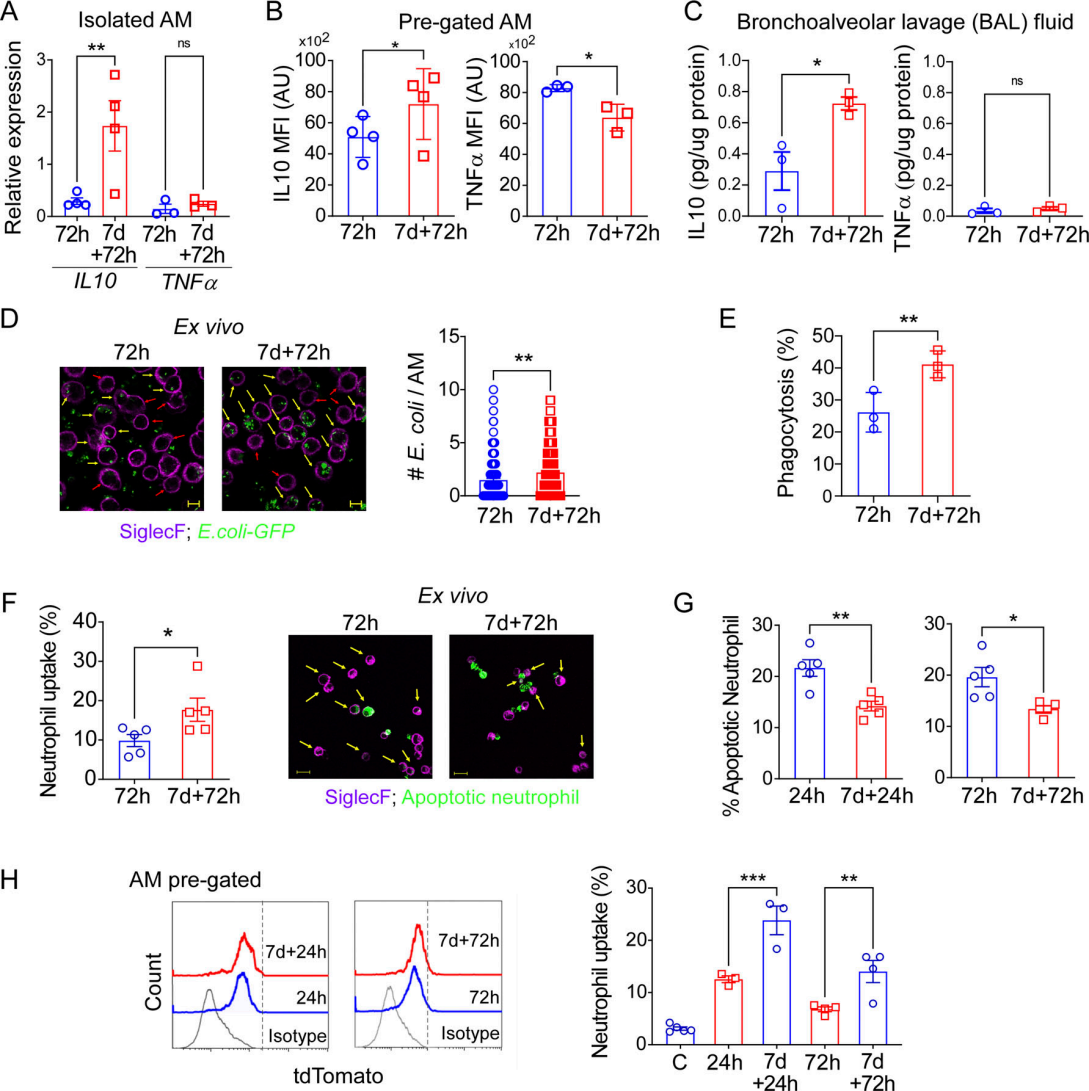
**Trained AMs exhibit enhanced efferocytosis. (A)** Relative mRNA expression of *IL-10* and *TNFα* was analyzed from flow-sorted AM from naïve and trained mice at 72 h after injury. Data were collected from four independent experiments (*n* = 3–4) and were analyzed by ANOVA with Bonferroni’s multiple comparisons test. **(B)** MFI (mean fluorescence intensity) of TNFα and IL-10 protein within naïve and trained AMs (CD45^+^CD64^+^CD11b^lo/−^SiglecF^+^ pre-gated) at 72 h after LPS was analyzed by flow cytometry. Data were collected from three independent experiments (*n* = 3–4) and were analyzed by unpaired *t* test. **(C)** The levels of IL-10 and TNFα were measured in BAL fluid by ELISA in the afore-mentioned conditions. Data were collected from three independent experiments (*n* = 3) and analyzed by unpaired *t* test. **(D)** Confocal microscopy shows the ex vivo phagocytosis of *E. coli*–GFP by naïve and trained AMs, isolated at 72 h after LPS injury, and the number of *E. coli* internalized by AMs as quantified. Scale bar, 10 µm. Data were collected from three independent experiments (*n* = 3) and were analyzed by unpaired *t* test. **(E)** Percent uptake of *E. coli*–GFP by AMs (CD45^+^CD64^+^CD11b^−/lo^SiglecF^+^ gated) was analyzed by flow cytometry. Data were collected from three independent experiments, (*n* = 3) and were analyzed by unpaired *t* test. **(F)** Percent uptake of dead neutrophils by naïve and trained AMs, isolated at 72 h after LPS, was quantified by flow cytometry (left panel). Data were collected from three independent experiments, (*n* = 3) and were analyzed by unpaired *t* test. Representative confocal image shows the efferocytosis in the aforementioned conditions (right panel). Scale bar, 10 µm. **(G)** Percentage of Annexin V^+^ neutrophil at 24 h after LPS (left panel) and 72 h after LPS (right panel) in naïve and trained mice was analyzed by flow cytometry. Data were collected from six independent experiments (*n* = 5) and were analyzed by unpaired *t* test. **(H)** In the left panel, flow cytometry histogram plot shows tdTomato positivity within AM (CD45^+^CD64^+^CD11c^+^ CD11b^lo/^SiglecF^+^gated). In the right panel, the percent uptake of neutrophils (tdTomato^+^) by naïve and trained AMs at 24 and 72 h after LPS challenge was analyzed by flow cytometry. Data were collected from three independent experiments (*n* = 3–4) and were analyzed by ANOVA with Bonferroni’s multiple comparisons test. Graphs show mean ± SD, with each dot representing individual mice. *P < 0.05, **P < 0.01, ***P < 0.001, ****P < 0.0001.

We investigated the cytokine profile of trained AMs following the second insult. We observed augmented expression of anti-inflammatory cytokine IL-10 in trained AMs without any increase in the proinflammatory cytokine TNFα level (Fig. 3, A and B). We also quantified the levels of IL-10 and TNFα in BAL fluid by ELISA at 72 h after injury. We observed significantly higher levels of the anti-inflammatory cytokine IL-10 in the trained after injury condition, whereas TNFα level remained low in both conditions (Fig. 3 C).

We next addressed the functional properties of trained AMs. Our previous transcriptomic analysis suggested upregulation of the genes associated with phagocytosis, endocytosis, and injury resolution (Fig. 1 I). First, to compare the phagocytic capacity of trained AMs and naïve AMs, we isolated AMs from naïve or trained mice subjected to LPS exposure and assessed phagocytosis of GFP-labeled *Escherichia coli*. Trained AMs demonstrated significantly higher bacterial phagocytosis as compared with naïve AMs (Fig. 3, D and E). As the transcriptomic analysis has also shown upregulation of receptor-mediated endocytosis and injury resolution after training, we next analyzed efferocytosis because it represents a form of phagocytosis that removes dead cells and proinflammatory cell debris and thus contributes to injury resolution (Mehrotra and Ravichandran, 2022; Yurdagul et al., 2017, 2020). When AMs were exposed to fluorescently labeled dead neutrophils, we observed a doubling of efferocytosis in trained AMs as compared with naïve AMs by quantitative flow cytometry as well as by confocal microscopy (Fig. 3 F). The increased ex vivo capacity of trained AMs to clear dead neutrophils corresponded to our in vivo observations showing the reduced presence of apoptotic neutrophils number in trained mice challenged with LPS (Fig. 3 G), consistent with rapid elimination of apoptotic neutrophils by trained AMs. To specifically quantify the in vivo uptake of apoptotic neutrophils by AMs in the naïve and training conditions, we utilized a mouse model in which all neutrophils were genetically labeled with red fluorescent tdTomato protein (“catchup mice”; Hasenberg et al., 2015) and performed the single and repeated LPS exposures. Lungs were isolated at early (24 h after LPS) and late (72 h after LPS) phases following the LPS challenge, followed by flow cytometry to quantify the percentage of AMs internalizing red fluorescent neutrophils. We observed that in naïve mice there was minimal uptake of labeled neutrophils by AMs, consistent with the minimal presence of neutrophils during homeostasis (Fig. 3 H). In the injury setting, trained AMs demonstrated a >50% increase in the uptake of apoptotic neutrophils in vivo when compared with untrained AMs (Fig. 3 H). These results suggested that increased efferocytosis is a key feature of trained immunity that facilitates injury resolution.

### Training upregulates key efferocytosis mediators in tissue-resident AMs

**Figure 4.**
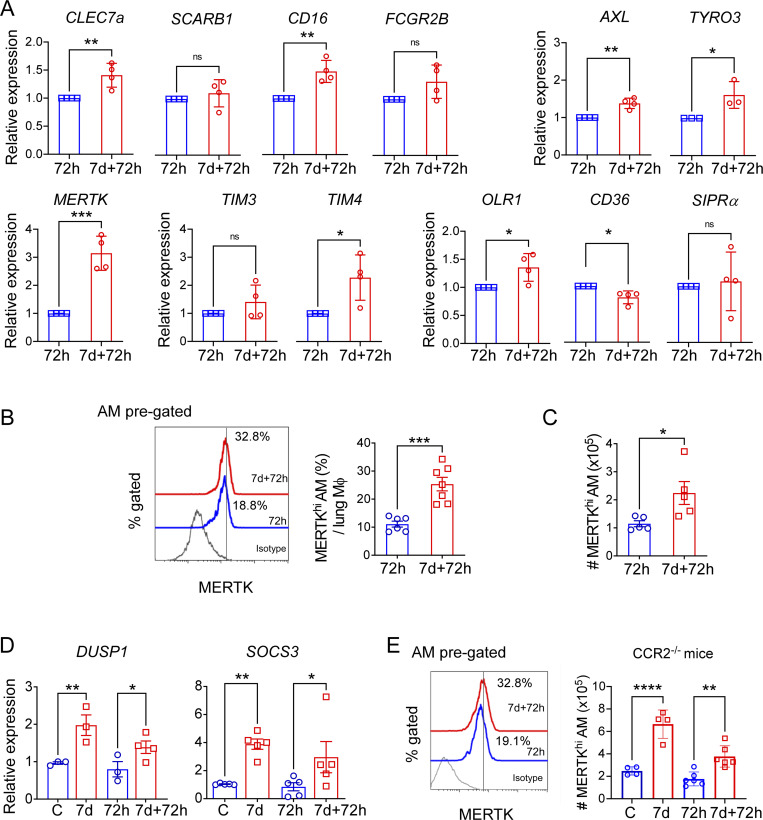
**Training upregulates key efferocytosis mediators in tissue-resident AMs. (A)** The relative mRNA levels for phagocytosis and efferocytosis genes (*CLEC7a*, *CD16*, *SCARB1*, *FCGR2B*, *TYRO3*, *AXL*, *OLR1*, *MERTK*, *TIM3*, *TIM4*, *CD36*, *SIPRα*) in naïve and trained AM at 72 h after LPS challenge are shown. Data were collected from four independent experiments (*n* = 4) and were analyzed by unpaired *t* test. **(B)** In the left panel, histogram overlay shows MERTK expression in AM (CD45^+^CD64^+^CD11b^lo/−^SiglecF^+^ gated) in naïve and trained mice at 72 h after LPS. In the right panel, the graph shows the percentage of MERTK^hi^ AMs (CD11b^−/lo^SiglecF^+^MERTK^hi^) subset within all lung macrophages (CD45^+^CD64^+^Gr1^−^ pre-gated) in above-mentioned conditions. Data were collected from four independent experiments, (*n* = 6–7) and were analyzed by unpaired *t* test. **(C)** The absolute number of MERTK^hi^ AMs (CD11b^−/lo^SiglecF^+^MERTK^hi^) subset within all lung macrophages (CD45^+^CD64^+^Gr1^−^ pre-gated) was quantified. Data were collected from three independent experiments (*n* = 5) and were analyzed by unpaired *t* test. **(D)** Relative mRNA expression of *SOCS3* and *DUSP1* in naïve and trained AMs, isolated at after 72 h LPS, were analyzed by qPCR. Data were collected from three independent experiments (*n* = 3–5) and were analyzed by ANOVA with Bonferroni’s multiple comparisons test. **(E)** In the left panel, histogram overlay shows MERTK expression in AM (CD45^+^CD64^+^CD11b^lo/−^SiglecF^+^ gated) in naïve and trained CCR2^−/−^ mice at 72 h after LPS. In the right panel, graph shows the absolute number of MERTK^hi^ AMs (CD11b^−/lo^SiglecF^+^MERTK^hi^) subset within all lung macrophages (CD45^+^CD64^+^Gr1^−^ pre-gated) in above-mentioned conditions. Data were collected from four independent experiments (*n* = 4–6) and were analyzed by ANOVA with Bonferroni’s multiple comparisons test. For all qPCR experiments *PPIA* was used as the internal control. Graphs show mean ± SD, with each dot representing individual mice. *P < 0.05, **P < 0.01, ***P < 0.001, ****P < 0.0001.

To study the mechanisms of increased efferocytosis of trained AMs, we assessed the mRNA expression of several established phagocytosis receptors, including *CD16*, *SCARB1*, *CLEC7a*, *FCGB*, as well as efferocytosis receptors such as *MERTK*, *TYRO3*, *AXL*, *TIM3*, *TIM4*, *OLR1*, *CD36*, and *SIPRα* (Fig. 4 A). Trained AMs showed increased expression of some general phagocytosis receptors like *CD16* and *CLEC7a*. All of the three components of the TAM receptor complex (*TYRO3*, *AXL*, and *MERTK*) as well as *TIM4*, a known efferocytosis mediator, showed increased relative mRNA expression levels in trained AM (Fig. 4 A). However, the overall mRNA expression levels of *TYRO3*, as well as *TIM4*, were found to be very low in AMs (Fig. S2 J), thus indicating that they may not be relevant for the enhanced efferocytosis we observed in trained AM. Moreover, the scavenger receptor *OLR1* was found to be also upregulated by training whereas the expression levels of *CD36* and *SIPRα* were not upregulated by training. Although we observed increased expression of *TIM4* mRNA, we did not find any significant change in surface expression as detected by CyTOF. On the other hand, our flow cytometry data confirmed the increased surface expression of MERTK, a key gatekeeper of efferocytosis (Cai et al., 2018), in the AMs of trained mice as compared with naïve mice (Fig. 4 B). Quantification of the absolute cell number showed that this difference became even more prominent following acute LPS exposure when the MERTK^hi^ AM population constituted 25% of the total lung macrophage population in trained mice whereas MERTK^hi^ AM comprised only 10% of the total lung macrophages in naïve mice (Fig. 4 C). We next examined the expression of genes known to be upregulated by MERTK activation, *SOCS3*, and *DUSP1*, which suppress inflammatory TLR signaling (Roberts et al., 2017; Rothlin et al., 2007), thus downregulating inflammation during efferocytosis. Trained AMs expressed higher levels of *SOCS3* and *DUSP1* mRNA (Fig. 4 D). We have also assessed the MERTK^hi^ AM population in CCR2^−/−^ mice. The results showed persistence of the training-induced increase of MERTK^hi^ AM, underscoring that circulating monocytes do not contribute to this increased population of MERTK^hi^ AMs following injury (Fig. 4 E).

**Figure S3.**
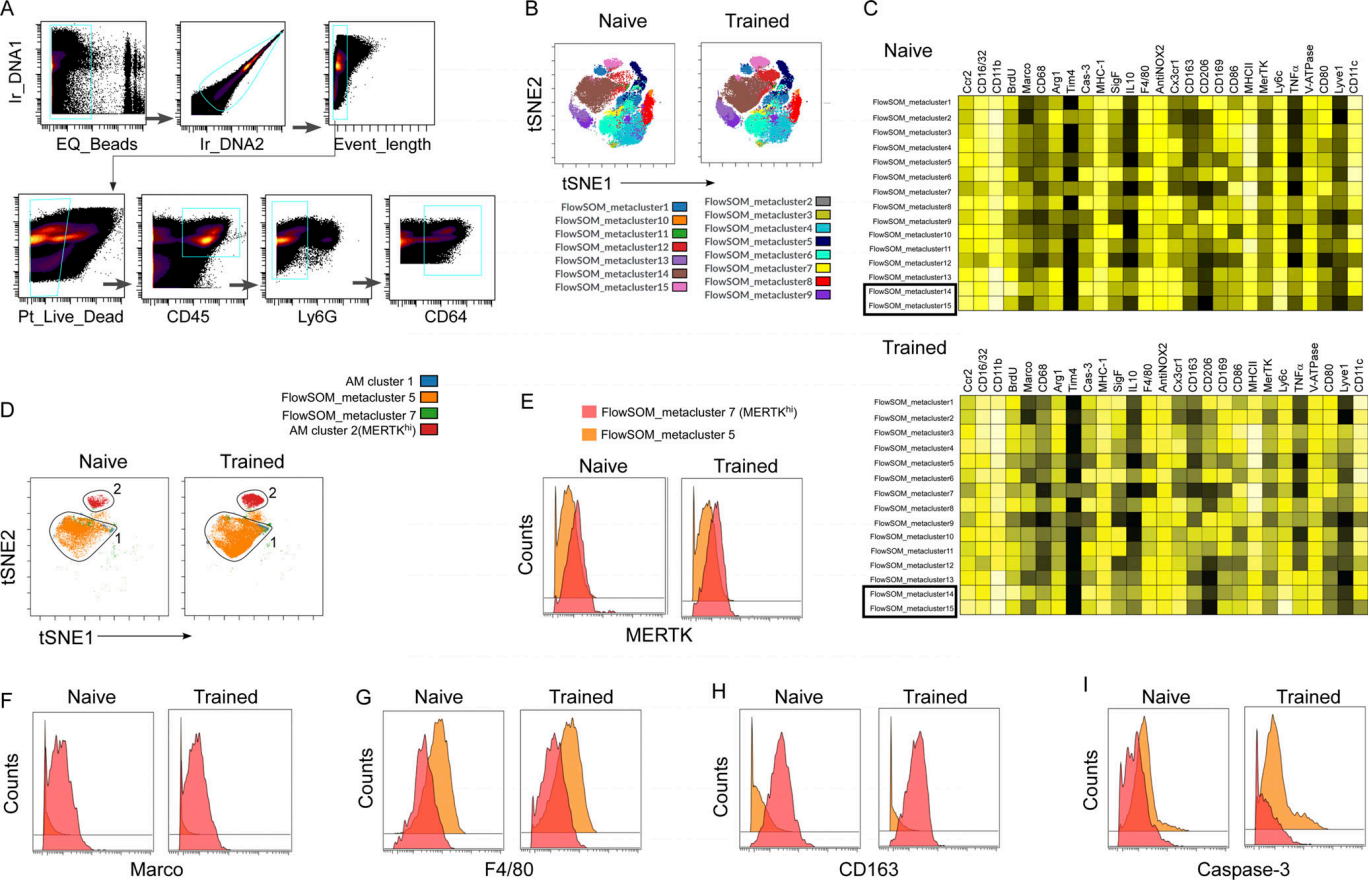
**Sub-population of lung macrophages in trained mice. (A)** The gating strategy used to enrich the macrophage population for further analysis in CyTOF. **(B)** The represented tSNE plots show the metaclusters, generated by the unsupervised clustering algorithm FlowSOM in naïve and trained macrophages at 72 h after LPS challenge. **(C)** Heatmap showing expression of 28 proteins that generated 15 metaclusters of naïve or trained macrophages. The representative heatmap was generated from individual samples using log_2_ ratio of mean intensity as the Z score. For all represented tSNE plots are analyzed with cells from *n* = 3 mice per group and repeated independently three times. **(D)** Representative viSNE plot shows the overlay of FlowSOM-metacluster_5,7 with AM cluster 1,2. **(E–I)** The representative histogram overlay shows the mean expression of (E) MERTK, (F) Marco, (G) F4/80, (H) CD163, (I) active caspase-3 in FlowSOM metacluster_5 (i.e., AM 1) and FlowSOM metacluster_7 (i.e., AM 2/MERTK^hi^) in trained mice at 72 h after LPS challenge. All the representative histogram plots are analyzed with cells from *n* = 3 mice per group and repeated independently three times.

### High-dimensional single-cell analysis of lung macrophages by mass cytometry identifies the expansion of MERTK^hi^ AM subpopulation after training

**Figure 5.**
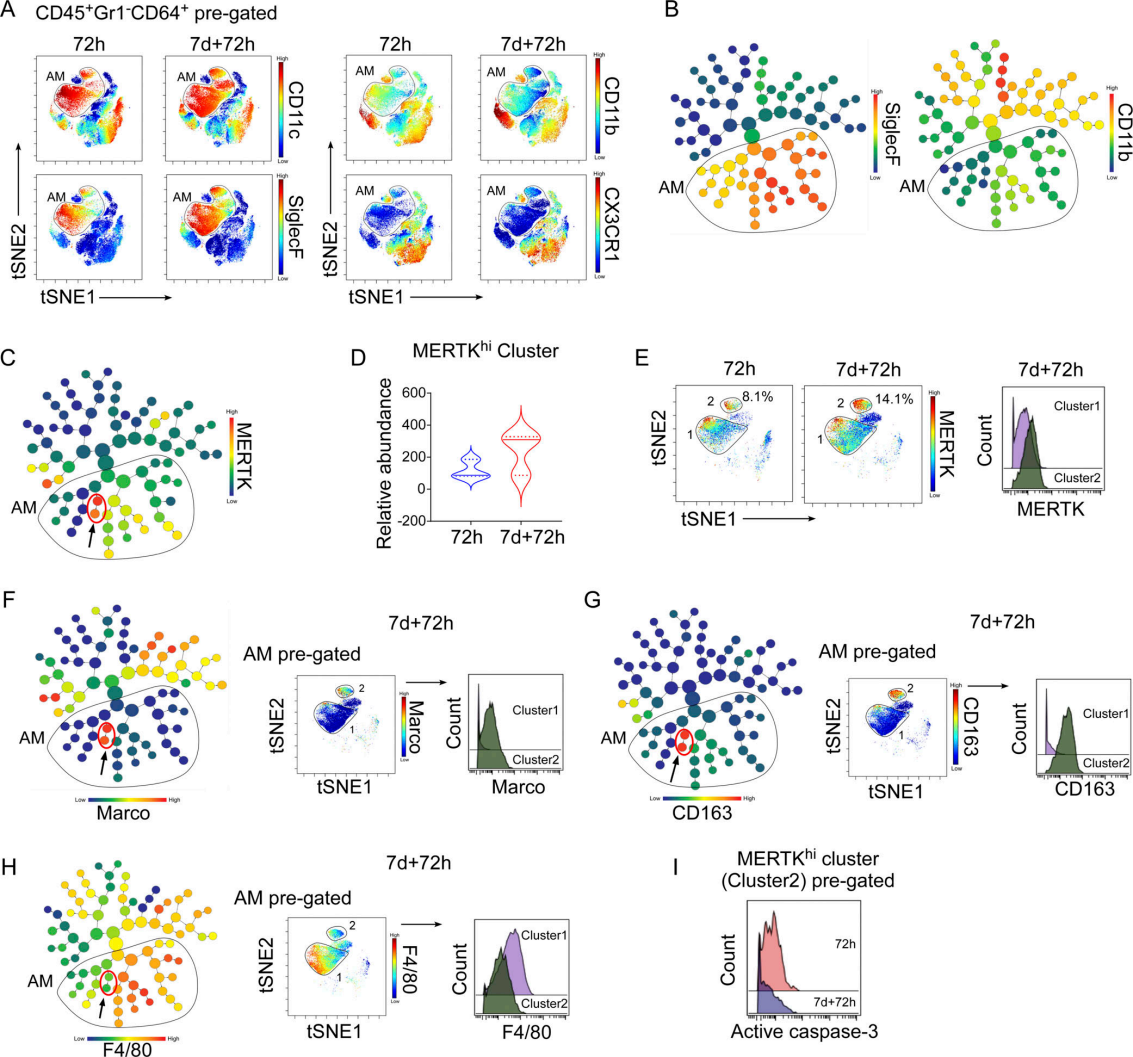
**Mass cytometry analysis of training-induced lung macrophage heterogeneity. (A)** tSNE map was derived by tSNE algorithm from CyTOF data of lung macrophages (CD45^+^CD64^+^Gr1^−^ gated) from naïve and trained mice at 72 h after LPS. Plots show the colored gradient expression of CD11c, SiglecF, CD11b, and CX3CR1 in different clusters. AM (CD45^+^CD64^+^Gr1^−^CD11c^+^SiglecF^+^CD11b^lo^CX3CR1^−^) clusters are highlighted by gating. **(B)** CyTOF data of lung macrophages (CD45^+^CD64^+^Gr1^−^ gated) is analyzed by the automated unsupervised hierarchical cell clustering algorithm CITRUS. The representation of CITRUS-tree with colored gradient expression of SiglecF and CD11b. The cluster nodes belonging to AM (CD45^+^CD64^+^Gr1^−^CD11c^+^ CD11b^lo^SiglecF^+^) are marked with a gate. **(C)** Colored gradient expression of MERTK is represented in CITRUS-tree and higher expressing cluster nodes are highlighted with a red circle. **(D)** The violin plot shows the relative abundances of MERTK^hi^ cluster in naïve and trained mice at 72 h after LPS. **(E)** The tSNE plot shows the colored gradient expression and percentage of MERTK^hi^ cluster (2) within pre-gated AM (CD45^+^CD64^+^Gr1^−^CD11b^lo^SiglecF^+^) in above-mentioned condition (left panel). In the right panel, the histogram overlay shows the mean expression of MERTK within cluster 1 and cluster 2 in trained AMs. **(F–H)** In the left panel, CITRUS-tree shows colored gradient expression of Marco, CD163, and F4/80; MERTK^hi^ clusters node is highlighted with red circles. In the right panel, representative viSNE plot shows the colored gradient expression of the above within pre-gated AM (CD45^+^CD64^+^Gr1^−^CD11b^lo^SiglecF^+^) in trained mice at 72 h after LPS challenge. The histogram overlay shows the mean expression of Marco, CD163, and F4/80 within cluster 1 and cluster 2 (MERTK^hi^) of pre-gated AM (CD45^+^CD64^+^Gr1^−^CD11b^lo^SiglecF^+^) in above mentioned condition. **(I)** The histogram overlay shows the mean expression of active caspase-3 within cluster 2 (MERTK^hi^) of pre-gated AM (CD45^+^CD64^+^Gr1^−^CD11b^lo^SiglecF^+^) in naïve and trained mice at 72 h after LPS challenge. For all CyTOF data, representative tSNE plots are analyzed with cells from *n* = 3 mice per group obtained from three independent experiments with a total *n* = 9 samples. Statistical analysis was performed using CITRUS.

We next carried out unbiased immunophenotyping of lung macrophages using CyTOF to assess whether increased surface MERTK expression constituted a defining feature of trained AMs. We generated a panel of 28 antibodies (Table S2) for known markers associated with macrophage phenotypes and functions, including an antibody targeting MERTK, the upregulated efferocytosis mediator and regulator identified in Fig. 4 B. Single live CD45^+^Gr1^−^CD64^+^ cells were pre-gated by a sequence of gating steps (Fig. S3 A) to enrich for macrophages. After standardizing each sample to 50,000 cells, we generated the viSNE plots that showed 9–10 clusters of macrophages from naïve and trained lungs 72 h after LPS challenge (Fig. 5 A). Depending on the differential expression of CD11c, SiglecF, CD11b, and CX3CR1, we have manually gated three AMs’ clusters (i.e., 1–3) which show higher expression of CD11c and SiglecF and low expression of CD11b and CX3CR1 that resemble the AMs’ phenotype (CD11c^+^SiglecF^+^CD11b^−/lo^CX3CR1^−^; Fig. 5 A). While viSNE is well-suited for subjective visual assessment of clusters, we also ran the unsupervised clustering algorithm FlowSOM (Grandi et al., 2020; Van Gassen et al., 2015) and identified 15 clusters, nearly all of which mapped to the major clusters seen viSNE (Fig. S3 B). The differential protein expression of the markers allowed us to identify distinct macrophage clusters for individual samples (Fig. S3 C). When we overlaid the FlowSOM_metaclusters on viSNE clusters, we observed that FlowSOM_metacluster-5 and 7 overlapped with the AM Clusters 1 and 2 (MERTK^hi^), respectively (Fig. S3 D).

To identify the cell markers that best defined the subpopulations that responded to training, we used an unsupervised hierarchical clustering algorithm CITRUS (Bruggner et al., 2014). We first distinguished the clusters of AMs and gated based on SiglecF and CD11b expression (Fig. 5 B). Among the AM-gated clusters, we identified two phenotypic clusters which expressed a higher level of MERTK (Fig. 5 C) and the MERTK^hi^ cluster showed higher frequency in trained mice as compared to naïve mice (Fig. 5 D). Next, we mapped back the MERTK^hi^ cluster in the viSNE plots of gated AM (CD45^+^Gr1^−^CD64^+^CD11b^−/lo^SiglecF^+^) and found these overlapped with AM cluster-2 and were increased in trained mice (Fig. 5 E, left panel). In parallel, we used the clustering algorithm FlowSOM which allowed us to compare expression levels of defined proteins between clusters and compared FlowSOM_5 (cluster 1) with FlowSOM_7 (cluster 2; Fig. S3). When comparing the mean expression of MERTK between AM cluster 1 and AM cluster 2, cluster 2 showed markedly higher MERTK expression in trained mice (Fig. 5 E, right panel; and Fig. S3 E). Together these data demonstrated that an unbiased analysis of potential training-specific AM markers identified MERTK^hi^ as a hallmark of trained AM. Our unbiased CITRUS analysis showed that the MERTK^hi^ cluster (cluster 2 in viSNE) was also characterized by high expression of Marco (also a mediator of efferocytosis) and CD163 as well as lower expression of F4/80 when compared with other AM clusters (cluster 1; Fig. 5, F–H; and Fig. S3, F–H). Next, we checked the active caspase-3 level in this MERTK^hi^ cluster (cluster 2) and found that the MERTK^hi^ cluster showed a significantly lower level of active caspase-3 in trained mice as compared with naïve mice (Fig. 5 I and Fig. S3 I), consistent with our results in Fig. 2, showing resilience to LPS-induced apoptosis as the cause of higher AM number in trained mice.

### KLF4 is upregulated during training and promotes MERTK^hi^ AM expansion

**Figure S4.**
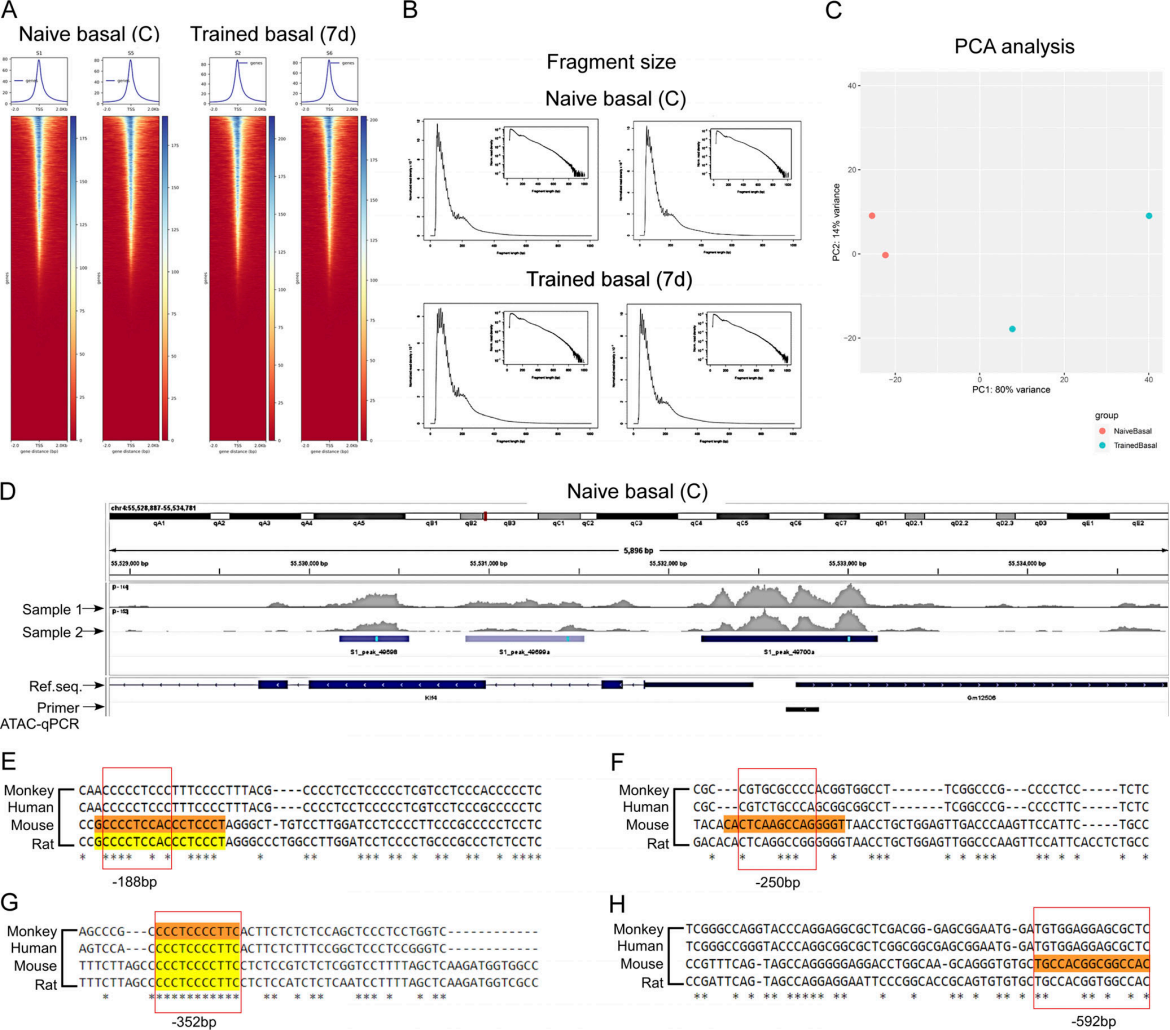
**Transcriptional regulation of trained AMs. (A)** ATAC-seq TSS reads coverage heatmap of all samples. Within the heatmap, each line represents a transcript. The reads coverage is summarized with a color code from red to blue. Red indicates no coverage; blue indicates the maximum coverage observed. All the TSS were aligned in the middle, with a 2 kb account the TSS displayed. On top of the heatmap, is the mean coverage signal distribution around TSS. The left two panels represent the naïve AM replicates, and the right two panels represent the trained AM replicates. **(B)** ATAC-seq fragment length distribution of all samples is shown. Distribution of read length for QC. X axis represents the fragment length in base pair. Y axis represents the normalized read density. The small figure is the log-transformed density distribution. Reads <100 bp represent the nucleosome-free region. **(C)** Principal component analysis (PCA) plot of ATAC-seq of all samples. ATAC-seq analysis generates peak file for each replicate. Each sample was characterized by the peak count table. After normalization and log-transformation, samples were grouped by biological condition. Red represents replicates from the naïve group. Blue represents replicates from the trained group. **(D)** The IGV (Integrative Genomics Viewer) snapshot shows the open chromatin regions within the *KLF4* promoter from basal AMs (control). The black short stretch region shows the amplicon region for the primers used for ATAC-qPCR. **(E–H)** Sequence conservation of the KLF4-binding sites (E) −188 bp, (F) −250 bp, (G) −352 bp, and (H) −592 bp in *MERTK* promoter between human, mouse, monkey, and rat was analyzed by CLUSTALW.

**Figure 6.**
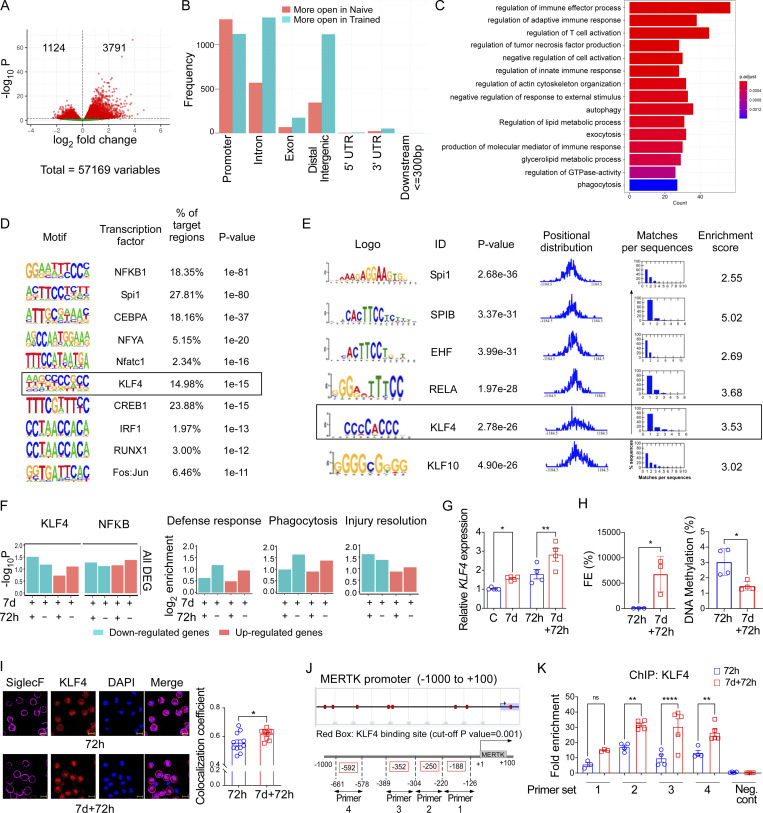
**TF KLF4 is upregulated during training and promotes MERTK**^**hi**^ **AM expansion. (A)** The volcano plot shows the DARs from ATAC-seq analysis. X axis indicates the log_2_ fold change. Y axis is a log_10_-transformed adjusted P value using DESeq2 analysis. Red indicated significant with FDR <0.05. **(B)** The bar plot shows the distribution of the genomic location of DARs. DARs genomic annotation were grouped into promoter, intron, exon, distal intergenic, 5′ UTR and -3′ UTR, and downstream ≤300 bp. Red indicates log_2_ fold change is larger in the naïve group, and blue indicates log_2_ fold change is larger in the basal group. **(C)** GO enrichment analysis of the genes with promoter more open in the trained group is represented. Fisher’s exact test was applied with a FDR <0.05. X axis is the gene number; y axis is the corresponding GO term. The color shades represent the adjusted P value from the enrichment analysis. **(D)** The TF motif enrichment was analyzed by Homer. The top 10 TF motifs were enriched from the more open promoter DARs from the trained group using Homer. FDR <0.05 were applied. Each row is a TF motif, with sequence logo details. **(E)** The TF motif enrichment was performed using MEME. The top six TF motifs were enriched from the more open promoter DARs from the trained group using MEME. Each row is a TF motif, with sequence logo details. Data were collected from two independent experiments with two mice in each experiment with a total *n* = 4. **(F)** The bar diagram shows the KLF4- and NF-κB–binding motif enrichment in the differentially expressed genes between trained AMs and naïve AMs (left panel). In the right panel, bar diagrams represent the KLF4-binding motif enrichment in the upregulated genes associated with host defense, phagocytosis, and inflammatory injury resolution, respectively. Data were collected from three independent experiments, and cells were combined from two mice per group with *n* = 6 samples. **(G)** Relative mRNA expression of *KLF4* in flow-sorted naïve and trained AMs at baseline and 72 h after LPS challenge was represented. *PPIA* was used as the internal control. Data were collected from three independent experiments (*n* = 4–5) and were analyzed by ANOVA with Bonferroni’s multiple comparisons test. **(H)** The graph shows the percent FE of chromatin accessibility of *KLF4* promoter (−600 bp) in naïve and trained AMs isolated 72 h after LPS challenge, detected by ATAC-qPCR by designed primer (left panel). The DNA methylation of the *KLF4* promoter was analyzed by methylation-specific PCRethylation-specific PCR)-qPCR in the above-mentioned condition and shown graphically (right panel). Data were collected from six independent experiments, and cells were combined from two mice per group with *n* = 6–8 samples and analyzed by unpaired *t* test. **(I)** KLF4 colocalization with DAPI in naïve and trained AMs, isolated from 72 h post-LPS challenge lung (left panel), was measured by confocal microscopy. Scale bar, 10 µm. Pearson’s r was quantified by ImageJ and represented graphically (right panel). Data were collected from three independent experiments (*n* = 4) and were analyzed by unpaired *t* test. **(J)** The upper panel shows putative binding sites of KLF4 on *MERTK* promoter (−1,000 to +100 bp relative to TSS), and the bottom panel shows the primer sets used for ChIP-qPCR. **(K)** FE of KLF4 binding on *MERTK* promoter in isolated 72-h post-LPS naïve and trained AMs was analyzed by ChIP-qPCR. Data were collected from five independent experiments (*n* = 3–5) and were analyzed by ANOVA with Bonferroni’s multiple comparisons test. Graphs show mean ± SD with each dot representing individual mouse data. *P < 0.05, **P < 0.01, ****P < 0.0001.

Next, we addressed the question of whether trained AMs showed evidence of differential chromatin opening as a potential epigenetic mechanism underlying training. We performed ATAC-seq (assay for transposase-accessible chromatin with sequencing) of naïve vs. trained AM (Fig. S4, A–C). We analyzed differentially accessible regions (DARs) in the chromatin of the naïve basal group and trained basal group. As shown in Fig. 6 A, we identified 4,951 significant (false discovery rate [FDR] ≤ 0.05) DARs from 57,169 consensus ATAC peak regions, which accounted for 8.6% of the total regions. Among these, 3,791 regions were significantly more accessible in the trained basal group and 1,124 regions were less accessible in the trained basal group compared with the naïve basal group. Each significant DAR is located in one of the seven major types of genomic regions, as shown in Fig. 6 B, including promoters, introns, exons, distal intergenic, 5′ untranslated regions (UTRs), 3′ UTRs, and downstream regions. The majority of DARs in our study were located within promoters, introns, and distal intergenic regions. The trained basal group showed more DARs located within introns than the naïve group (Fig. 6 B). Next, we analyzed more open promoter DARs and linked them to corresponding genes to determine the biological processes which were more likely to be activated after the training. These genes with their promoters, significantly more open after the challenge, were significantly enriched in several cellular processes (FDR ≤ 0.05), such as regulation of immune effector process, regulation of adaptive immune response, autophagy, exocytosis and phagocytosis, and other 10 related GO terms (Fig. 6 C). Next, we performed TF binding motif enrichment analysis using these promoter DARs that are more accessible from the trained group to infer which TFs were more likely to be active in the trained condition. To increase the robustness of our analysis, we applied two independent approaches, Homer (Heinz et al., 2010) and MEME suite (Bailey et al., 2015), for the enrichment analysis. Interestingly, both methods showed that the TF KLF4 ranked among the top 10 TFs in terms of TF-binding site enrichment in the differentially open promoter regions (Fig. 6, D and E).

In silico analysis of our transcriptomic data (RNA-seq) data comparing naïve AMs and trained AMs concordantly showed that the promoters of the differentially expressed genes were enriched for the binding motifs of the TFs KLF4 and NF-κB (Fig. 6 F, left panel). The promoters of upregulated genes associated with injury resolution were enriched for KLF4 binding motifs (Fig. 6 F, right panel), suggesting that the proresolving phenotype of trained AMs was likely driven by KLF4-mediated transcription. Trained AMs also expressed higher *KLF4* mRNA than naïve AMs (Fig. 6 G). Next, we compared the chromatin accessibility of the *KLF4* promoter (+100 to −600 bp) between trained and naïve AMs by using ATAC-qPCR (quantitative PCR) with our designed primer sets. The results showed that the chromatin accessibility was significantly higher in trained AM as compared with naïve AM 72 h after LPS challenge (Fig. 6 H, left panel). However, naïve AMs showed no chromatin accessibility by ATAC-qPCR despite some degree of mRNA expression at that time point (Fig. 6 G). Therefore, we matched the predicted ATAC-qPCR amplicon with the *KLF4* promoter regions as assessed by ATAC-seq, which provides a more comprehensive assessment of chromatin accessibility (Fig. S4 D). We found that the ATAC-qPCR amplicon for the *KLF4* promoter stretched into a region with a portion of closed chromatin (“valley”), which likely resulted in the impaired amplification and thus underestimated the extent of chromatin accessibility of the *KLF4* promoter. We also assessed the DNA methylation of the *KLF4* promoter, and results showed that DNA methylation is significantly higher in naïve AMs as compared with trained AMs (Fig. 6 H, right panel). Next, we evaluated the protein expression of KLF4 by confocal microscopy of isolated trained and naïve AMs. We observed intracellular localization of KLF4 and greater nuclear localization of KLF4 in trained AMs (Fig. 6 I) indicating increased KLF4 transcriptional activity in trained AMs. Computational analysis of the *MERTK* promoter predicted five putative KLF4-binding sites at −592, −352, −250, and −188 bp upstream of the transcription start site (TSS; Fig. 6 J, upper panel), which are evolutionarily conserved (Fig. S4, E–H). We assessed the active binding of KLF4 to the *MERTK* promoter using four sets of overlapping primers from −661 to −124 bp region of the *MERTK* promoter (Fig. 6 J, lower panel). Chromatin immunoprecipitation (ChIP) assay showed positive ChIP signals for all KLF4-binding sites in AMs; however, the relative binding of KLF4 was significantly greater in trained AMs as compared with naïve AMs at −250 and −352 bp sites (Fig. 6 K), indicative of increased KLF4-mediated transcription of the efferocytosis regulator *MERTK* in trained AMs.

### KLF4 promotes MERTK^hi^ AM-mediated injury resolution in trained mice

**Figure S5.**
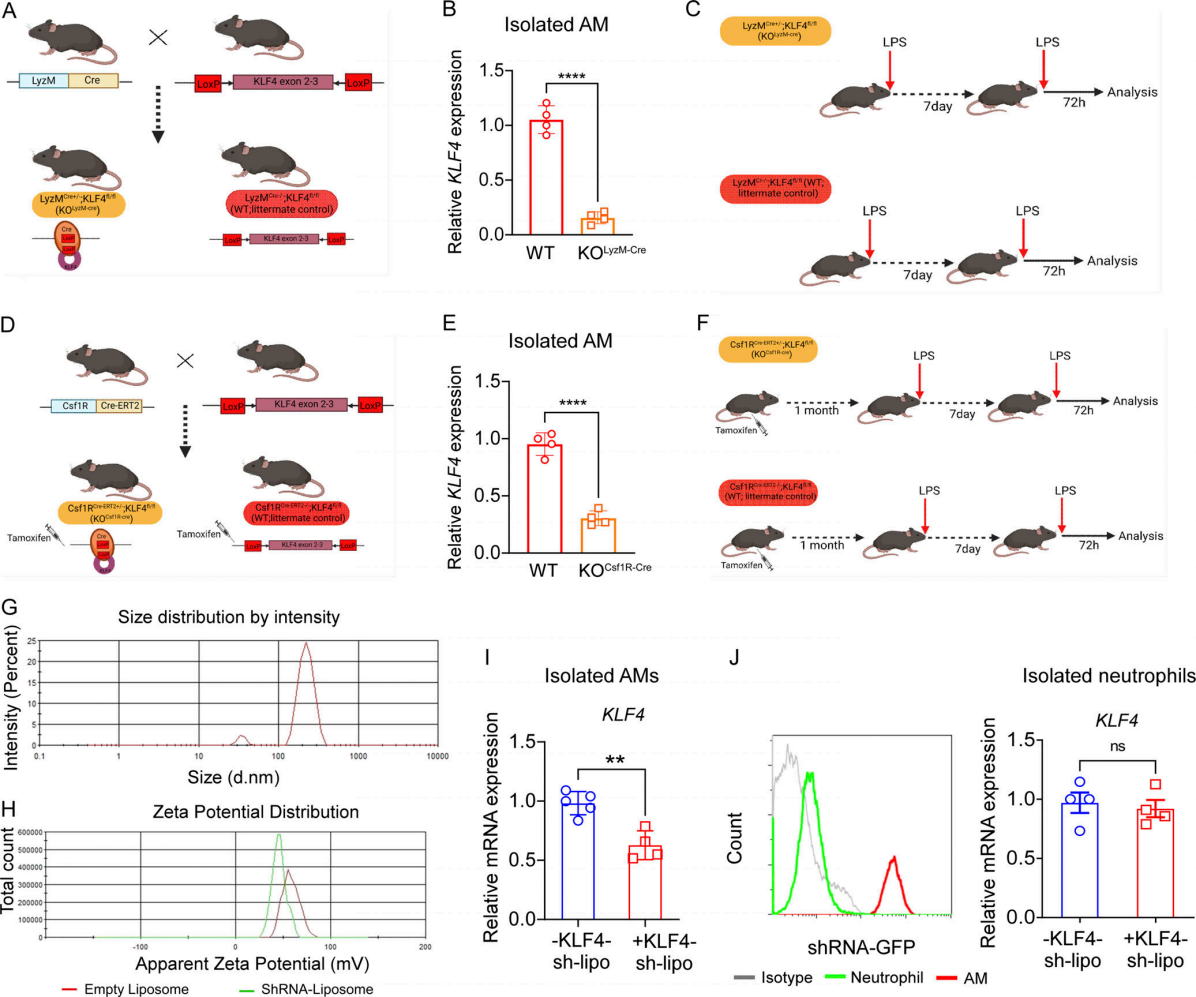
**Training in KLF4-depleted AM. (A)** Representation of breeding scheme of LyzM-^Cre+/−;^KLF^fl/fl^ (KO^LyzM-Cre^) mice. **(B)** Relative mRNA expression of *KLF4* in KO^LyzM-Cre^ and WT (littermate control) in trained AM. Data were collected from four independent experiments (*n* = 4) and were analyzed by unpaired *t* test. **(C)** Training scheme of KO^LyzM-Cre^ and littermate control mice. **(D)** Representation of breeding scheme of Csf1R-^CreERT2+/−;^KLF^fl/fl^ (KO^Csf1R-CreERT2^) mice. **(E)** Relative mRNA expression of *KLF4* in KO^Csf1R-CreERT2^ and WT (littermate control) in trained AM. Data were collected from four independent experiments (*n* = 4) and were analyzed by unpaired *t* test. **(F)** Training scheme of KO^Csf1R-CreERT2^ and littermate control mice. **(G and H)** The graphs show the prepared liposome size analyzed by dynamic light scattering (G) and the zeta potential of empty liposome and shRNA-liposome (H), as studied by Malvern Zetasizer. **(I)** The graph represents the *KLF4*-mRNA expression in isolated trained AMs in the presence of scramble/KLF4-shRNA-liposomes. Data were collected from three independent experiments (*n* = 4–5) and were analyzed by unpaired *t* test. **(J)** The histogram overlay shows the GFP expression in the gated neutrophils in green (CD45^+^Ly6G^+^) and gated AMs (CD45^+^CD64^+^CD11b^−^SiglecF^+^) in red (left panel) in KLF4-shRNA–treated trained mice. The *KLF4* mRNA expression in isolated neutrophils from KLF4-shRNA–treated and corresponding control mice in the 7 d + 72 h condition was measured. Data were collected from three independent experiments (*n* = 4) and were analyzed by unpaired *t* test. *PPAI* has been used as the internal control in qPCR. Graphs show mean ± SD with each dot representing an individual mouse data. **P < 0.01, ****P < 0.0001.

**Figure 7.**
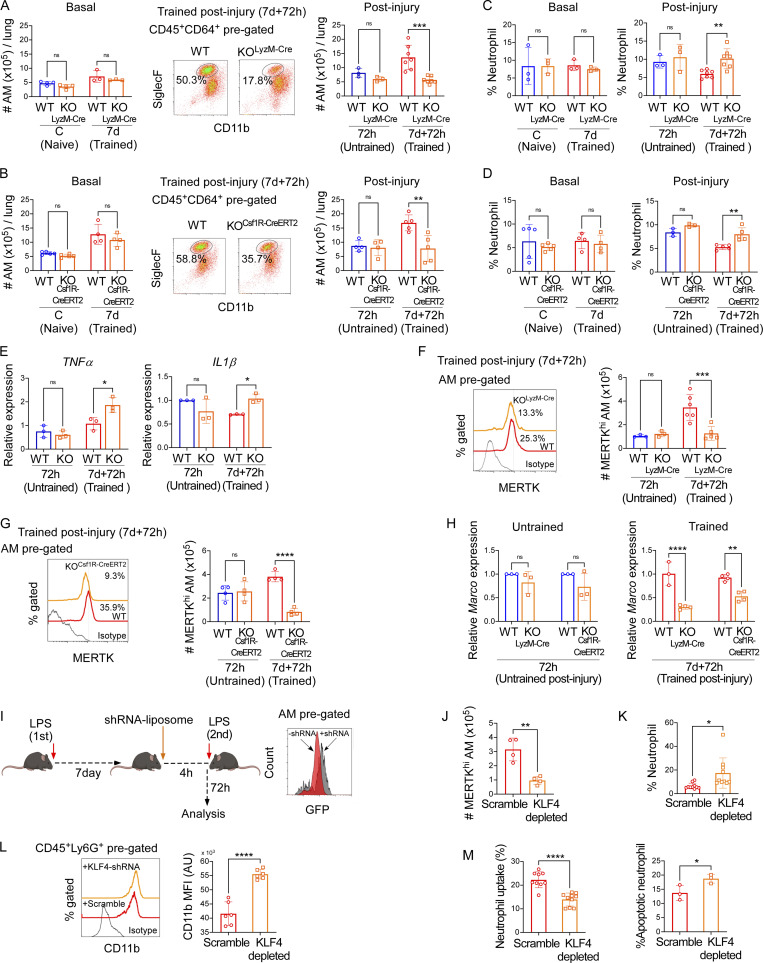
**KLF4 is required for MERTK**^**hi**^ **AM expansion in trained mice. (A)** The left panel shows the absolute AM number at the basal condition in naïve (C) and trained (7 d) KO^lyzM-cre^ mice and WT littermate control mice. In the middle panel, the representative flow cytometry plots show the percentage of AMs (CD45^+^CD64^+^CD11b^−/lo^SiglecF^+^) within CD45^+^CD64^+^ (lung macrophages) pre-gated population in LPS (7 d)-trained WT littermate control and KO^lyzM-cre^ at 72 h after LPS challenge. The graph in the right panel represents the absolute AM number in above mentioned conditions. Data were collected from six independent experiments (*n* = 3–6) and were analyzed by ANOVA with Bonferroni’s multiple comparisons test. **(B)** The left panel shows the absolute AM number at the basal condition in naïve (C) and trained (7 d) KO^Csf1R-CreERT2^ and WT littermate control mice. In the middle panel, the representative flow cytometry plots show the percentage of AMs (CD45^+^CD64^+^CD11b^−/lo^SiglecF^+^) within CD45^+^CD64^+^ (lung macrophages) pre-gated population, in LPS (7 d)-trained WT littermate control (WT) and KO^Csf1R-CreERT2^ at 72 h after LPS challenge. The graph in the right panel represents the absolute AM number in the above-mentioned conditions. Data were collected from six independent experiments (*n* = 4–5) and were analyzed by ANOVA with Bonferroni’s multiple comparisons test. **(C)** The graph shows the percentages of neutrophils in KO^lyzM-Cre^ and WT littermate control mice at basal conditions (C and 7 d; left panel) and at 72-h post-LPS challenge conditions. Data were collected from six independent experiments (*n* = 3–7) and were analyzed by ANOVA with Bonferroni’s multiple comparisons test. **(D)** The percentages of neutrophils in KO^Csf1R-CreERT2^ and WT littermate control mice at basal conditions (C and 7 d; left panel) and 72 h after LPS challenge were represented (right panel). Data were collected from six independent experiments (*n* = 3–5) and were analyzed by ANOVA with Bonferroni’s multiple comparisons test. **(E)** The relative mRNA expression of *TNFα* and *IL1β* in total lung tissue isolated from untrained and trained WT littermate control and KO^Csf1R-CreERT2^ mice 72 h after LPS challenge. Data were collected from six independent experiments (*n* = 3) and were analyzed by ANOVA with Bonferroni’s multiple comparisons test. **(F)** The histogram overlay shows the MERTK expression and percent positivity within pre-gated AM (CD45^+^CD64^+^CD11b^−/lo^SiglecF^+^) in trained littermate control (WT) and KO^lyzM-Cre^ at 72 h after LPS challenge (left panel). The graph in the right panel shows the absolute number of MERTK^hi^ AM in untrained and trained KO^lyzM-Cre^ and WT mice at 72 h after LPS challenge. Data were collected from six independent experiments (*n* = 3–6) and were analyzed by ANOVA with Bonferroni’s multiple comparisons test. **(G)** The histogram overlay shows the MERTK expression and percent positivity within pre-gated-AM (CD45^+^CD64^+^CD11b^−/lo^SiglecF^+^) in trained littermate control (WT) and KO^Csf1R-CreERT2^ mice at 72 h after LPS challenge (left panel). The absolute number of MERTK^hi^ AM in untrained and trained KO^Csf1R-CreERT2^ and WT mice at 72 h after LPS challenge is shown in the right panel. Data were collected from six independent experiments (*n* = 4) and were analyzed by ANOVA with Bonferroni’s multiple comparisons test. **(H)** The relative *Marco* mRNA expression in isolated 72 h after LPS–challenged untrained (left panel) and LPS (7 d)-trained AM (right panel) in KO^lyzM-Cre^ and KO^Csf1R-CreERT2^ mice and respective littermate controls (WT) is represented. Data were collected from six independent experiments (*n* = 3–4) and were analyzed by ANOVA with Bonferroni’s multiple comparisons test. **(I)** Schematic representation of KLF4-shRNA-liposome delivery experiment in trained mice (left panel). In the right panel, representative histogram overlay shows the mean expression of GFP (KLF4-shRNA) in AM (CD45^+^CD64^+^CD11c^+^CD11b^lo/−^SiglecF^+^) pre-gated cells in untreated and KLF4-shRNA-liposome–injected trained mice. **(J)** Absolute numbers of MERTK^hi^ AM within the whole lung of scrambled- and KLF4 shRNA-liposome–injected trained mice were shown. Data were collected from three independent experiments (*n* = 4) and were analyzed by unpaired *t* test. **(K)** Neutrophil percentage within all lung immune cells (CD45^+^ gated) was analyzed in the aforementioned condition. Data were collected from five independent experiments (*n* = 10) and were analyzed by unpaired *t* test. **(L)** Histogram overlay shows the mean expression of CD11b in pre-gated neutrophils (CD45^+^Ly6G^+^) in the absence or presence of KLF4 shRNA-liposome (left panel) and mean fluorescence intensity (MFI) are statistically analyzed (right panel). Data were collected from three independent experiments (*n* = 6) and were analyzed by unpaired *t* test. **(M)** In trained catchup mice, the percent uptake of tdTomato-labeled neutrophil by AMs in the absence or presence of KLF4-shRNA-liposomes was analyzed by flow cytometry (left panel). Data were collected from five independent experiments (*n* = 10) and were analyzed by unpaired *t* test. Flow cytometry analysis of annexin V positivity of neutrophil (CD45^+^Ly6G^+^) in above-mentioned conditions is represented graphically (right panel). Data were collected from three independent experiments, (*n* = 3) and were analyzed by unpaired *t* test. For all qPCR experiments, *PPIA* was used as the internal control. Graphs show mean ± SD, with each dot representing individual mouse data. *P < 0.05, **P < 0.01, ***P < 0.001, ****P < 0.0001.

To define the mechanistic and causal involvement of KLF4 in AM training, we generated myeloid-specific constitutive KLF4 deletion mice; LyzM-Cre^+/−^KLF4^fl/fl^ (KO^LyzM-Cre^; Fig. S5 A). Deletion of KLF4 in isolated AMs from KO^LyzM-Cre^ mice was confirmed by qPCR (Fig. S5 B). The KO^LyzM-Cre^ mice and the littermate control (WT) were trained with LPS at 7-d intervals and analyzed 72 h after second-LPS challenge (Fig. S5 C). First, we assessed the absolute numbers of AMs in KLF4- KO^LyzM-Cre^ and WT mice at basal conditions in naïve and 7-d trained mice (C and 7 d, respectively). The results showed that there was no significant change in the AM number during baseline in KLF4- KO^LyzM-Cre^ vs. WT mice (Fig. 7 A, left panel). Next, we analyzed the AM number 72 h after LPS challenge in both untrained and trained conditions. Results showed that in trained mice, the number of AMs was significantly lower in KLF4- KO^LyzM-Cre^ mice as compared with the WT (Fig. 7 A, middle right panel), whereas in untrained after injury conditions, there was no significant change (Fig. 7 A, right panel), which suggested that myeloid KLF4 was required for the training-induced increase in AM numbers. Even though LyzM-Cre–mediated deletion is very effective, it also results in the deletion of targeted genes in neutrophils. We therefore developed a more specific genetic deletion model to study macrophage-specific KLF4 deletion in mice using a Csf1R-Cre-ERT2. After breeding of Csf1R-Cre^ERT2+/−^KLF4^fl/fl^ (KO^Csf1R-CreERT2^) mice, tamoxifen was used to induce KLF4 deletion specifically in macrophages (Fig. S5 D), and the deletion of KLF4 in isolated AMs was confirmed by qPCR (Fig. S5 E). These mice were trained similarly (Fig. S5 F) and we used flow cytometry to compare the AM number in KO^Csf1R-CreERT2^ vs. WT at basal conditions (C and 7 d). Results showed that there was no significant difference in AM numbers between KO^Csf1R-CreERT2^ and WT mice at baseline conditions (Fig. 7 B, left panel). Further analysis confirmed that at 72 h after injury, the absolute number of AMs in KO^Csf1R-CreERT2^ mice was significantly lower as compared with littermate control (WT) in trained mice (Fig. 7 B, middle right panel), whereas it remained unchanged in untrained mice (Fig. 7 B, right panel).

Next, we compared the neutrophil percentages in both KO^LyzM-Cre^ and KO^Csf1R-CreERT2^ vs. WT mice at naïve (C) and trained (7 d) basal conditions and found no significant differences (Fig. 7, C and D, left panels). However, we observed that at 72 h after LPS challenge, the percentage of neutrophils in lungs in trained KLF4 deletion mice was significantly higher than WT (Fig. 7, C and D. right panel), suggesting that deletion of KLF4 in macrophages prevents the training-induced augmentation of injury resolution. Importantly, in the absence of training, KO^LyzM-Cre^ or KO^Csf1R-Cre^ mice were similar to WT mice (Fig. 7, C and D, right panel), which suggested that the KLF4 role was specific to the training. Importantly, the inflammatory cytokine (i.e., *TNFα*, *IL1β*) expression in the lung was significantly higher in KO^Csf1R-Cre^ trained mice when compared to trained WT, again supporting a key role for KLF4 in macrophages promoting injury resolution (Fig. 7 E).

Next, we evaluated the MERTK^hi^ AM population in KLF4-KO untrained and trained mice 72 h after LPS challenge. We observed that in both the KO^LyzM-Cre^ and KO^Csf1R-CreERT2^ trained mice, the percentage and the absolute number of MERTK^hi^ AMs was significantly lower (Fig. 7, F and G). However, KLF4 deletion in untrained mice did not show any significant changes in MERTK^hi^ AM at 72 h after injury (Fig. 7, F and G). As we had observed (Fig. 5) that MERTK^hi^ AM also expressed a higher level of the efferocytosis mediator Marco, we assessed *Marco* levels in KO^LyzM-Cre^ and KO^Csf1R-CreERT2^ mice and found that the reduction in MERTK was mirrored by a reduction of *Marco* in KO^LyzM-Cre^ and KO^Csf1R-CreERT2^ trained mice but not in untrained mice (Fig. 7 H), suggesting that KLF4 in AMs drives the gene expression of efferocytosis mediators induced by training.

We next evaluated the in vivo efferocytosis efficiency of trained AMs following LPS challenge. We depleted KLF4 in AMs by intratracheal (i.t.) liposome delivery of shRNA targeting KLF4 and encoding GFP for the purpose of assessing targeted delivery. The liposomes were well dispersed in water, and their hydrodynamic diameter was ∼229 nm, with a PDI of 0.3, as measured by dynamic light scattering (Fig. S5 G). Due to the electronegative nature of siRNA, the zeta potential of shRNA-loaded liposomes was reduced (42 mV at 25°C) compared with blank liposomes, which had a zeta potential of 58 mV (Fig. S5 H). KLF4 shRNA-loaded liposomes were injected i.t. into trained catchup (tdTomato-labeled neutrophil) mice (which had been challenged with LPS 7 d before). After liposomal delivery, mice were challenged again with LPS and assessed at 72 h after a repeat LPS challenge (Fig. 7 I, left panel). Flow cytometry analysis confirmed the presence of KLF4-shRNA-liposomes within AMs by GFP expression as well as KLF4 knockdown in the AMs (Fig. 7 I, right panel; and Fig. S5 I). To assess the specificity of liposome KLF4-shRNA delivery, we compared the GFP expression of KLF4-shRNA within the gated AM or gated neutrophil population. As shown in the histogram overlay (Fig. S5 J, left panel), the neutrophil population (green) did not show any noticeable GFP expression, whereas the AM population did (red), which suggests negligible uptake of KLF4-shRNA by neutrophils. We also found no significant reduction in *KLF4* mRNA expression in isolated neutrophils (Fig. S5 J, right panel).

Depletion of KLF4 markedly reduced the numbers of MERTK^hi^ AMs in trained mice 72 h after LPS (Fig. 7 J). Consistent with our earlier results, depletion of KLF4 in trained AMs resulted in greater neutrophil accumulation in the lungs (Fig. 7 K), which showed augmented expression of adhesion molecule CD11b and indicated higher levels of proinflammatory neutrophil activation (Fig. 7 L). Furthermore, we observed that in vivo efferocytosis of neutrophil debris was markedly reduced following KLF4 depletion in trained AMs (Fig. 7 M, left panel), consistent with the increased number of apoptotic neutrophils in lungs (Fig. 7 M, right panel). These results together show the obligatory role of KLF4 in the reprogramming of pro-resolving trained AMs.

### Adoptive transfer of trained AMs prevents inflammatory injury and promotes resolution in PA pneumonia

**Figure 8.**
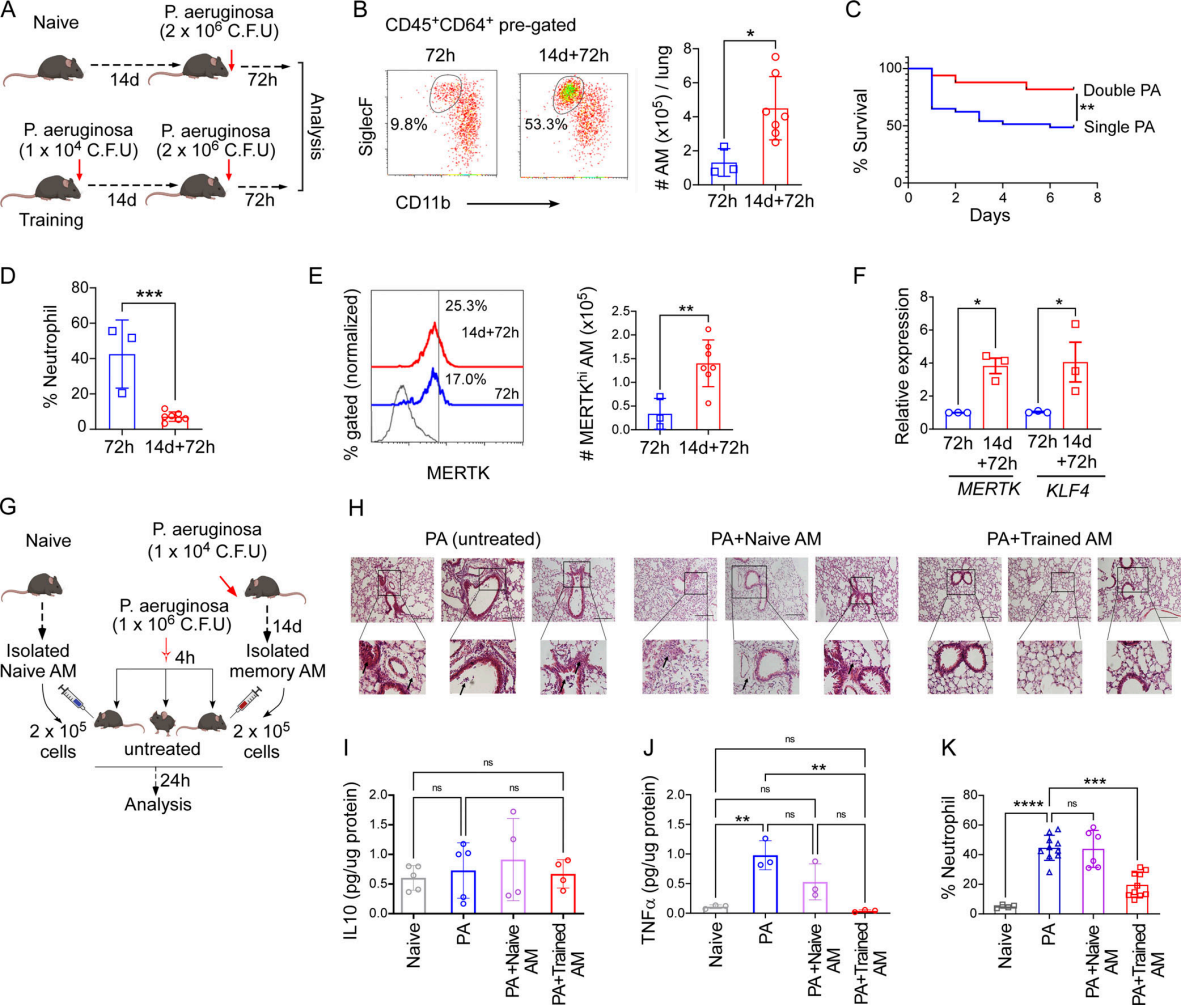
**Adoptive transfer of trained AMs confers protection in recipient mice with PA-induced pneumonia. (A)** Schematic showing training in PA-induced pneumonia model. One group of mice received a sublethal dose of PA (1 × 10^4^ CFU) intranasally to induce pneumonia. After 14 d, along with a naïve group of mice, PA-challenged mice were again challenged with a lethal dose of PA (2 × 10^6^ CFU) and after 72 h, all mice were sacrificed for further analysis. **(B)** In the left panel, representative flow cytometry plots show the naïve and trained AM population (CD45^+^CD64^+^CD11b^−/lo^SiglecF^+^) in the 72 h post-acute PA insult (72 h and 14 d + 72 h). In the right panel, AM absolute numbers were quantified. Data were collected from three independent experiments, (*n* = 3–7) and were analyzed by unpaired *t* test. **(C)** The survival curve shows the percent survival of naïve and trained mice after counter PA (2 × 10^6^ CFU) challenge. Data were collected from three independent experiments (*n* = 21) and were analyzed by Kaplan–Meier survival analysis. **(D)** Graph shows the percentage of neutrophils (CD45^+^Ly6G^+^) within total lung immune cells (CD45^+^ pre-gated) in naïve and trained mice at 72 h after the second PA insult. Data were collected from three independent experiments (*n* = 3–7) and were analyzed by unpaired *t* test. **(E)** The histogram overlay shows the MERTK positivity within pre-gated AM in naïve and PA-trained mice (left panel). In the right panel, the absolute number of MERTK^hi^ AM is shown. Data were collected from three independent experiments (*n* = 3–7) and were analyzed by unpaired *t* test. **(F)** The relative mRNA expression of *MERTK* and *KLF4* in isolated AMs from naïve and trained mice following a PA challenge. Data were collected from three independent experiments (*n* = 3) and were analyzed by ANOVA with Bonferroni’s multiple comparisons test. **(G)** Schematic showing the adoptive transfer of naïve and trained AMs. Trained AMs were collected from PA (1 × 10^4^ CFU) pre-challenged mice, and naïve AMs were collected from naïve mice. Three groups of naïve mice were inoculated with PA (1 × 10^6^ CFU). After 4 h, either 2 × 10^5^ trained AMs or 2 × 10^5^ naïve AMs were injected i.t., whereas a third group of mice received no cell therapy (PA). Then, after 24 h, all mice were sacrificed for further analysis. **(H)** H&E staining of lung sections show the lung histology of PA-infected untreated mice, naïve AM treated, and trained AM treated mice which were exposed to lethal PA. Scale bar, 100 µm. The box section has been enlarged to show the highlighted region in the bottom panel. Arrowhead shows the perivascular accumulation of leukocytes and perivascular edema. **(I and J)** The level of IL-10 and TNFα was measured from BAL fluid by ELISA in the aforementioned group of mice. Data were collected from three independent experiments (*n* = 4–5) and were analyzed by ANOVA with Bonferroni’s multiple comparisons test. **(K)** Flow cytometry quantification of the percentage of neutrophil (CD45^+^Ly6G^+^) within all lung immune cells was compared between PA-infected untreated (PA), naïve AM-treated (+naïve AM) and trained AM-treated (+trained AM) mice. Data were collected from three independent experiments (*n* = 4–10) and were analyzed by ANOVA with Bonferroni’s multiple comparisons test. Graphs show mean ± SD, with each dot representing an individual mouse data point. *P < 0.05, **P < 0.01, ***P < 0.001, ****P < 0.0001.

Next, we used the PA-induced pneumonia model of lung injury because of its translational relevance and to delineate the therapeutic potential of generating pro-resolving trained AMs. We challenged mice with i.t. PA infection with the sublethal dose of 1 × 10^4^ CFU PA intranasally and allowing for resolution of this initial injury (14 d). Subsequently, prechallenged mice were further infected with a lethal dose of 2 × 10^6^ CFU PA (Fig. 8 A). Corresponding to our previous endotoxin-training model, the AM number was significantly higher in PA-trained mice 72 h after insult (Fig. 8 B). Survival studies also showed that training with sublethal PA insult significantly decreased mortality in trained mice as compared with naïve mice following counter challenge with a lethal dose of PA (Fig. 8 C). When naïve mice were exposed to PA, the percentage of neutrophils increased to over 40% of the lung immune cells, whereas trained mice showed only minimal neutrophil accumulation after 72 h of counter PA challenge (<10% of total lung immune cells; Fig. 8 D), suggesting that—similar to LPS-induced training described above—PA-induced trained AMs dampened the extent of inflammatory lung injury. We next addressed whether PA-induced training also leads to the expansion of MERTK^hi^ AMs. The absolute number of MERTK^hi^ was assessed by flow cytometry and showed a significantly greater number in trained mice, comparable with endotoxin-trained mice (Fig. 8 E). We also checked the transcript level of *MERTK* and *KLF4* and it showed higher expression in trained AMs as compared with naïve Ams (Fig. 8 F).

Next, to determine whether promoting injury resolution was intrinsic to the trained AMs or a function of the lung AM niche, we performed adoptive cell transfer of trained AMs into naïve mice that were exposed to a lethal dose of PA (Fig. 8 G). One group of mice received sublethal 1 × 10^4^ CFU PA for the purpose of AM training, and after 14 d of recovery, the trained AMs were isolated from this group for adoptive transfer (Fig. 8 G). Next, we challenged three groups of mice with a lethal dose of 1 × 10^6^ CFU PA. Among them, the first group received an i.t. injection of 2 × 10^5^ trained AMs after receiving the lethal PA dose (Fig. 8 G). To compare the therapeutic efficacy of trained and naïve AMs, in the second group, 2 × 10^5^ naïve AMs were injected (i.t.), and the third group received no AMs (Fig. 8 G). Lung histology showed profound perivascular and peribronchial cellular accumulation as well as perivascular edema in PA-infected mice. The severity of lung injury was significantly reduced in the trained AM-treated mice as observed by markedly reduced accumulation of cells in perivascular or peri-bronchial regions whereas the treatment with naïve AM showed modest benefits (Fig. 8 H). We next quantified the levels of TNFα and IL-10 in the BAL fluid. The level of the anti-inflammatory cytokine IL-10 was not significantly affected by the infection (Fig. 8 I). However, as expected, the levels of the proinflammatory cytokine TNFα were significantly increased with infection (Fig. 8 J). Importantly, treatment with trained AM reduced levels to those seen in homeostasis, whereas treatment with naïve AM did not significantly impact TNFα levels (Fig. 8 J). Furthermore, neutrophil accumulation was significantly increased at 24 h following the lethal PA challenge, but this increase was markedly blunted in mice receiving trained AMs as compared with the mice that received naïve AMs (Fig. 8 K). Together, all these results demonstrate that trained immunity is an intrinsic property of lung AMs and highlight the potential of adoptively transferring trained AMs to promote the resolution of the injury.

## Discussion

Trained innate immunity typically involves phenotype shifts in innate immune cells such as monocytes, macrophages, or NK cells without pathogen specificity (Min-Oo and Lanier, 2014; Netea et al., 2016; Saeed et al., 2014; Seeley et al., 2018). Recent studies have identified metabolic, epigenetic, and transcriptional mediators of trained immunity (Cheng et al., 2014; Ishii et al., 2009; Khan et al., 2020; Netea et al., 2016; Saeed et al., 2014) and shown that trained immunity is characterized by augmented immune responses with enhanced bacterial killing capacity (Quintin et al., 2012; Saeed et al., 2014). There are also reports which suggest that sequential pathogen exposures suppressed immune responses and lead to tolerogenic immune responses (Ishii et al., 2009; Seeley et al., 2018). However, the influence of trained immunity in the regulation of proresolving features and functions of AMs remains unknown.

Our current study focused on investigating features of trained immunity in lung macrophages. Using transcriptomic analysis and high dimensional mass cytometry, we observed that repeated exposure to bacterial endotoxin augmented the resilience and survival of AMs after pathogen challenge and increased their efferocytosis capacity mediated by the TF KLF4. The increased capacity for clearance of inflammatory cell debris by efferocytosis allowed trained AMs phenotype shifts that accelerated the resolution of inflammation. In a translationally relevant model of repeated PA pneumonia, we also observed that adoptive transfer of AMs, which had previously been exposed in vivo to the same bacteria, accelerated the resolution of lung injury and reduced mortality in recipient mice encountering de novo PA challenge. Taken together, our results show that in the epigenetically reprogrammed trained AM, KLF4 plays a critical role in triggering efferocytosis to accelerate injury resolution, and this feature of trained immunity is intrinsic to the tissue-resident AMs.

Exposure to pathogenic toxins such as the bacterial endotoxin LPS can result in the rapid depletion of tissue macrophages including AMs (Dagvadorj et al., 2015; Evavold et al., 2021). Depleted lung macrophage pools are replenished via proliferation of surviving tissue macrophages and influx of circulating CX3CR1^+^ or CCR2^+^ bone marrow–derived monocytes which differentiate into lung macrophages (Aegerter et al., 2020; Aran et al., 2019; Misharin et al., 2017). In our present study, we observed that in trained mice, the numbers of AMs were higher after exposure to a subsequent endotoxin or live PA challenge than AM numbers of mice without the preceding exposure, thus suggesting that increased AM numbers are one of the hallmarks of the training.

To precisely address whether bone marrow–derived circulating monocytes contributed to the higher AM number, we used genetic lineage tracing with CX3CR1^+^ reporter mice, a genetic model of impaired monocyte infiltration (*CCR2*^*−/−*^ mice), and clodronate mediated blood monocyte depletion experiments. All three approaches consistently demonstrated that the increased AM number during the training condition was not due to the increased recruitment of circulating monocytes. In an acute lung injury model, an increased level of IL4 causes AMs proliferation and lung remodeling (Vaz de Paula et al., 2020). However, post-injury proliferation of AMs was similar in naïve and trained mice. Instead, we found that the increased AM number following the repeat in vivo pathogen challenges was due to reduced apoptosis, and thus, our study highlights the potential reduction of apoptosis as another important feature of trained immunity.

A recent study has shown that the mice recovered from influenza infection are better protected from following *Streptococcus pneumoniae* infection, which is associated with a high IL-6–producing monocyte-derived AMs with enhanced bacterial killing capacity (Aegerter et al., 2020). Another study also reported a SiglecF^low^MHCII^high^ AM population in *S. pneumoniae* challenged mice, which was phenotypically different from resident AM and metabolically reprogrammed to control bacterial growth during the counter challenge (Guillon et al., 2020). Our studies here complement this prior work by using high-dimensional mass cytometry analysis to identify the MERTK^hi^ AM subpopulation that expands in trained mice upon receiving a second challenge. MERTK^hi^ AM are characterized by a specific combination of markers (Marco^hi^CD163^+^F4/80^low^). Our mass cytometry analysis also complements recent studies that have used single-cell RNA-seq analysis to address macrophage heterogeneity in the brain and lung microenvironments (Ochocka et al., 2021; Schyns et al., 2019). Previous single-cell RNA-seq analysis has identified two distinct subpopulations of lung IMs (Chakarov et al., 2019; Schyns et al., 2019). The identification of the MERTK^hi^ AM population in the present study underscores the value of using mass cytometry to identify different macrophage subsets in trained immunity as a complement to transcriptomic analysis because expression differences for some genes are more readily observed at the protein level.

Several studies have investigated how trained immunity in AMs improves bacterial killing (Aegerter et al., 2020; Guillon et al., 2020), which could possibly involve the upregulation of several phagocytosis receptors (Fossati et al., 2002; Mentrup et al., 2022; Zhang et al., 2019a). Efferocytosis, the clearance of cell debris, is essential for the resolution of inflammation because inefficient clearance of dead cells and debris amplifies inflammatory signaling (Cai et al., 2017; Elliott et al., 2017; Zhang et al., 2019b). Several receptors are reported to be associated with the efferocytosis processes, and among them, TAM receptors, i.e., TYRO3, AXL, and MERTK are known to play critical roles in the maintenance of homeostasis and promote reparative response in macrophages (Akalu et al., 2022; Chang et al., 2018). Among the three TAM receptors, MERTK is a critical component in the signaling cascade of efferocytosis which recognizes apoptotic cells via the evolutionary conserved “eat me” signal phosphatidylserine. It has been shown that disruption of MERTK activity severely compromised injury resolution in atherosclerosis (Cai et al., 2018; Cai et al., 2017). Our study complements these findings by emphasizing how trained immunity causes the expansion of MERTK^hi^ AMs which triggers injury resolution in pathogen-prechallenged mice by augmented uptake of neutrophilic debris in the injured lung microenvironment. Moreover, our data showing the upregulation of the scavenger receptor OLR1 in trained AM complements the recent finding that increased levels of oxidized low-density lipoprotein and C-reactive protein) exacerbated lung injury (Korkmaz et al., 2022) because trained AMs with high scavenger receptor expression could prevent injury-propagating high oxidized low-density lipoprotein concentrations. However, the expression of other efferocytosis receptors like CD36, which detects damage-associated molecular patterns and also acts as a fatty acid transporter (Chen et al., 2022), or SIPRα, which is involved in senescent cells clearance (Rothlin and Ghosh, 2023), was not increased in trained AMs, suggesting that primarily the efferocytosis programs in AMs are upregulated by training.

The proficiency of efferocytosis in trained AMs may be due to metabolic-epigenetic reprogramming. It has been reported that IL-4, which is upregulated in acute lung injury or acute respiratory distress syndrome (Vaz de Paula et al., 2020), along with apoptotic cells induces the tissue repair program of macrophages (Bosurgi et al., 2017). Efferocytosis at injured tissue loads the macrophages with metabolites, which leads to metabolic reprogramming via regulation of fatty acid oxidation (Zhang et al., 2019b) as well as solute carrier–mediated aerobic glycolysis that releases lactate (Morioka et al., 2018) and causes an escalation of different anti-inflammatory signatures, i.e., IL-10, VEGFα, and TGFβ (Morioka et al., 2018; Zhang et al., 2019b). Our transcriptomic analysis complements these reports by demonstrating that trained AMs which show enhanced efferocytosis efficiency, show upregulated genes associated with lipid metabolism (Korkmaz et al., 2022) and anti-inflammatory genes like IL-10 and VEGFα, as well as genes associated with injury resolution. Moreover, our data suggested that trained AMs shows the upregulation of gene associated with arginine metabolism, which is reported to be essential for prolonged efferocytosis (Yurdagul et al., 2020). Thus, the present study demonstrates that preferential release of anti-inflammatory cytokines and increased efferocytosis after injury are hallmarks of trained immunity that facilitate injury resolution.

We also addressed the transcriptional reprogramming underlying trained AM-induced accelerated efferocytosis and injury resolution. A previous study showed that the TF ATF7 reduced the expression of inflammatory chemokines in peritoneal macrophages and bone marrow–derived macrophages (Yoshida et al., 2015). On the other hand, C/EBPβ reports mediating memory reprogramming in hematopoietic stem cells exposed to LPS, which enhances bacterial killing capacity (de Laval et al., 2020). Our unbiased epigenetic analysis of chromatin accessibility showed that trained AMs are programmed to upregulate genes involved in phagocytosis, exocytosis, lipid metabolism, and that KLF4 targets are among the most differentially open chromatin regions in AMs following training. KLF4 is known to regulate tissue homeostasis and repair in the endothelial cells by promoting vascular integrity (Cowan et al., 2010) and regulating smooth muscle cell phenotype transition in atherosclerosis (Lou et al., 2020; Shankman et al., 2015). In macrophages, KLF4 regulates lipid metabolism and macrophage M2-like polarization (Liao et al., 2011) as well as TLR9 expression in peritoneal macrophages (Roberts et al., 2017). Our findings show that upregulated KLF4 transcriptionally induces the efferocytosis mediator MERTK in trained AMs and is essential for the enhanced efferocytosis proficiency of trained AMs following counter pathogen challenge, which helps to restore tissue homeostasis. Moreover, KLF4 is known to negatively regulate p53 transcription and thus inhibit apoptosis (Rowland et al., 2005) which explains the possible molecular mechanism associated with death resilience of trained AMs as in KLF4 knockout mice. In these mice, we observed a lower number of trained AMs following the counter-pathogen challenge. We observed only a modest increase in KLF4 expression, but TFs such as KLF4 are also regulated at a posttranslational level (Dhaliwal et al., 2019), and training may concomitantly affect KLF4 activity via additional regulatory mechanisms and its nuclear localization (Dhaliwal et al., 2018). Therefore, future studies addressing the importance of posttranslational regulation of KLF4 function in the trained immunity of tissue-resident macrophages may prove to be useful.

Prior studies suggest a critical role for the tissue niche in the programming of tissue-specific macrophages such as secretion of GM-CSF and TGFβ by type II alveolar epithelial cells which maintain AM identity via regulating PPARγ expression (Schneider et al., 2014; Yu et al., 2017). Similarly, endothelial and stellate cells instruct the differentiation of monocytes into liver macrophages (Kupffer cells) via BMP/TGFβ signaling (Bonnardel et al., 2019). This raises the intriguing question of whether the trained immunity of AMs is a function of niche memory or whether the memory is intrinsic to the AMs themselves. In our study, we observed that adoptive transfer of trained AMs, isolated from PA challenged mice, limited inflammatory lung injury of naïve recipient mice infected with PA. Thus, the pro-resolution AM phenotype induced by training appears to be intrinsic to the AMs. This finding does not rule out the possibility that niche cues from epithelial, stromal, or endothelial cells may also contribute to the reprogramming of trained AMs, but it does suggest that once AMs are reprogrammed, their pro-resolving function does not require continued niche input.

Cell therapies are emerging as an important therapeutic approach to limit inflammatory tissue injury in settings of deleterious inflammation such as autoimmune disease or transplantation (Wang et al., 2020). Transfer of autologous tolerogenic DCs can ameliorate type I diabetes and mitigate inflammation following kidney transplantation by downregulation of the required co-stimulatory signals to autoreactive lymphocytes (Bouchet-Delbos et al., 2021; Giannoukakis et al., 2011). Initial clinical studies suggest that allogeneic mesenchymal stromal cell transfer in drug-resistant systemic lupus erythematosus patients has the potential to reduce disease progression and improve survival (Wang et al., 2018). The profound efficacy of adoptively transferred trained AMs in the present study in reducing lung injury highlights the potential of developing AM-based cell therapies in which trained AMs are delivered during a severe lung infection as a means of reducing inflammation-induced lung injury.

In summary, our study identifies a lung-resident tissue macrophage population that expands with trained immunity and shows augmented efferocytosis induced by the TF KLF4. There are emerging studies that have identified potential cross-talk between the training of adaptive and innate immune cells. For example, a non-canonical pathway in T cells can modulate the trained immunity of macrophages after viral infections via the production of IFN-γ (Yao et al., 2018). Our focus was on the pro-resolving phenotype of trained AMs, but we cannot rule out additional co-regulation of this AM phenotype by adaptive immune cells. Future studies using distinct pathogens—viral and bacterial—could focus on characterizing potential cross-talk between adaptive immune cells and AMs. Furthermore, even though we established a key role for KLF4 as a regulator of enhanced efferocytosis in trained AMs to promote injury resolution, there may be additional TFs involved in establishing the pro-resolving trained AM phenotype which comprises death resilience or enhanced bacterial killing capacity (Aegerter et al., 2020; Guillon et al., 2020). According to our unbiased epigenetic analysis, trained AMs show increased chromatin accessibility for other TFs, and these could be the starting point for additional TF analyses to understand the genetic programs underlying AM training. In our present study, these pro-resolving and anti-inflammatory functions of trained AMs underscore their therapeutic potential in lung diseases with severe tissue injury and inflammation such as acute respiratory distress syndrome and autoimmune diseases.

## Materials and methods

### Mice

C57BL/6J, CX3CR1^CreERT2^ (Jax cat. no. 021160), B6.Cg-Gt(ROSA)26Sor^tm9(CAG-tdTomato)Hze^/J (tdTomato^fl/fl^; Jax cat. no 007909), LyzM^Cre^;B6.129P2-Lyz2^tm1(cre)Ifo/J^ (Jax cat. no. 004781), Csf1R^CreERT2^; B6J.FVB-Tg (Csf1r-icre)1Jwp/BacJ (Jax cat. no. 034470), and B6.129S4-Ccr2tm1Ifc/J mice were originally purchased from Jackson Laboratory, and KLF4fl/fl; B6.129S6-Klf4^tm1Khk^/Mmmh (Mutant Mouse Resource and Research Centers [MMRRC] cat. no. 29877) mice were originally purchased from MMRRC. tdTomato^fl/fl^ mice were crossed with CX3CR1^CreERT2^ (tamoxifen-inducible CX3CR1 monocyte-specific Cre recombinase) mice to generate inducible CX3CR1^CreERT2+^; tdTomato^fl/fl^ mice, lineage tracing mice. KLF4^fl/fl^ mice were crossed with LyzM^Cre^ and Csf1R^CreERT2^ (tamoxifen-inducible Csf1R Cre recombinase) mice to generate myeloid specific: LyzM^Cre+/−^;KLF4^fl/fl^ (KO^LyzM-Cre^) and monocyte-macrophage specific: Csf1R^CreERT2+/−^;KLF4^fl/fl^ (KO^Csf1R-Cre^) KLF4 knockout mice. Genotyping of mice strains was performed by regular PCR using the recommended primers on the Jackson Laboratory (https://www.jax.org) and the MMRRC (https://www.mmrrc.org) website. CX3CR1^CreERT2^ mice were administered tamoxifen (75 mg/kg body weight) via intraperitoneal injection every other day throughout the treatment for Cre induction (Aran et al., 2019). Csf1R^CreERT2+/−^;KLF4^fl/fl^ (KO^Csf1R-Cre^) mice were administered tamoxifen (75 mg/kg body weight) via intraperitoneal injection for consecutives 5 d for Cre induction. Catchup mice (Ly6g^tm2621(cre)Arte^; Hasenberg et al., 2015) were used for genetically labeling neutrophils in efferocytosis assays. Mice aged 6–8 wk were used for all experiments. All mice were housed in a temperature-controlled specific pathogen–free facility with a standard diet and water under 12-h light–dark cycles at the Animal Care Facility of the University of Illinois at Chicago. All animal experiments were conducted under National Institutes of Health guidelines for the Care and Use of Laboratory Animals and were approved by the Institutional Animal Care and Use Committee of the University of Illinois.

### Bacterial culture

GFP-tagged PA (PA-GFP-PA01 strain) and *E. coli*–GFP (#25922GFP; ATCC) were grown in LB broth in the presence of ampicillin (100 μg/ml) under constant shaking at 37°C overnight. For in vitro phagocytosis experiments, *E. coli*–GFP bacteria in the log growing phase (OD 0.4) were centrifuged (4,000 rpm, 10 min) and resuspended in DMEM without penicillin/streptomycin. For in vivo experiments, PA-GFP was titrated by serial dilution to calculate CFUs.

### Animal treatment

Trained responses were induced using either the bacterial endotoxin LPS or PA. 7 ml (concentration, 1 mg/ml) of LPS (*E. coli* O55:B5) was nebulized and given i.t. to mice. After 7 or 28 d (as indicated), mice were challenged with the same dose of LPS. For PA experiments, mice were challenged with 1 × 10^4^ CFU of PA intranasally. After 12–14 d, mice were again challenged with 1–2 × 10^6^ CFU of PA.

### Lung edema

Left lobes of lungs from mice were excised at defined time points following LPS-induced injury, weighed, and dried at 55°C for 24 h, and the lung wet-to-dry ratio was calculated as a measure of lung edema.

### Mouse lung single-cell suspensions

Mice were anesthetized with ketamine/xylazine and killed by cervical dislocation. Lungs were perfused with 10 ml of PBS via the right ventricle. The lungs were excised and minced with scissors, followed by enzymatic digestion with 1 mg/ml collagenase A (Millipore-Sigma) in a shaking water bath (37 °C) for 45–60 min. The digested lung pieces were forced through a 17-gauge needle and filtered by a 40-μm nylon mesh to obtain a single-cell suspension. The remaining red blood cells were lysed using RBC lysis buffer. The acquired lung single-cell suspensions were used for further applications, like flow cytometry, cell sorting, and CyTOF.

### BAL

BAL was performed after deep anesthesia with ketamine/xylazine, a brief cut in the mouse trachea was made, followed by slow injection of 1 ml of cold PBS into the lung using an 18-gauge blunt needle, aspirated back after 30 s, and repeated four times. Isolated cells were collected by centrifugation and further processed for flow cytometry or plated for ex vivo functional assays.

### Flow cytometry

For flow cytometry, lung cells and BAL cells were resuspended in FACS buffer (BD Bioscience) and incubated with Fc blocking antibody (Trustain; BioLegend) for 10 min to prevent nonspecific binding. Cells were incubated with the following fluorophore-labeled antibodies: anti-mouse CD45, CD64, Gr1, CD11c, CD11b, SiglecF, CD80, CD86, MHCII, and MERTK (BioLegend; Table S1). To analyze IL-10, TNFα cells were treated with Golgi plug/Golgi stop (BD Bioscience) for 3 h. Then cells were fixed and permeabilized using Cytofix/Cytoperm buffer (BD Bioscience) according to the manufacturer’s protocol. Next, treated cells were labeled with fluorophore-labeled antibodies: anti-mouse IL-10 and TNFα (BioLegend; Table S1). To evaluate the level of active caspase-3 in AMs, cells were surface-stained with antibodies, then fixed and permeabilized using Cytofix/Cytoperm buffer (BD Bioscience) according to the manufacturer’s instructions, followed by antibody staining with anti-active caspase-3 antibody (BD Bioscience). To check KLF4 expression, cells were fixed and permeabilized with eBioscience Transcription Factor Staining Buffer Set and labeled with anti-KLF4 antibodies (Biorbyt; Table S1). According to the experimental requirement panels for the lungs, macs were modified to minimize spectral overlap between antibody-conjugated fluorescent dyes. Samples were run on Gallios and CytoFLEX S Flow Cytometer (Beckman), and data were analyzed by Kaluza Analysis 2.1 (Beckman) and Flowjo software. For FACS, samples were run on Moflo Astrios, and the cells were collected in DMEM media until further processed.

### Blood monocyte depletion

To deplete blood monocytes, 50 μl clodronate liposomes (Encapsula Nanosciences) were i.v. injected through the retro-orbital vein. After 48 h, monocytes were depleted by >90%, as validated by flow cytometry.

### BrdU assay of proliferation

Mice were injected i.p. with BrdU (#B5002; Sigma-Aldrich) 75 mg/kg 24 h before sacrifice. After sacrifice, the lungs were flushed with PBS and the single-cell suspension was used for flow cytometry and CyTOF. For flow cytometry, anti-Brdu antibody (BioLegend; Table S1) was used for staining according to the manufacturers protocol.

### Cytometry by CyTOF

We used the CyTOF for high-dimensional analysis of cell surface markers, cytokines, and signaling molecules simultaneously at the single-cell level. A panel of 37 metal-labeled antibodies (CD45, CD16/32, CD64, CD11b, CD11c, CD206, CD80, CD86, MHC-II (I-A/I-E), MHC-I, CX3CR1, F4/80, CD169, Ly6G, Ly6C, CD19, CD4, CD8, NK1.1, Epcam, TNFα, IL-10, and active Caspase-3 were purchased from FLUDIGM (Table S2). Primary antibodies for SiglecF, MERTK, Tim4, Marco, CD24, Arginase-1 (Arg1), CCR2, CD68, V-ATPase, Anti-NOX2, Lyve1, CD163, CD31, CD103, and BrdU were purchased from BD Bioscience, R&D Biosystems, BioLegend, and Thermo Fischer (Table S2), and labeled with FLUDIGM metal labeling kit (Maxpar X8 metal Labeling Kit) according to the manufacturer’s protocol. Cells were treated with Golgistop/GolgiPlug (BD Bioscience) for 2–3 h at 37°C and stained according to the FLUIDIGM recommended protocol. Samples were run on the Helios CyTOF mass cytometer (FLUIDIGM) at the flow cytometry core of the Research Resources Center of the University of Illinois at Chicago. Data from CyTOF were analyzed using the Cytobank online analysis tool (https://www.cytobank.org).

### tSNE (t-distributed stochastic neighbor embedding) and FlowSOM analysis

Manually gated singlet viable cells (Fig. S1 C) from naïve and trained mice were subjected to dimensionality reduction algorithms viSNE/tSNE (Amir el et al., 2013) analysis (https://www.cytobank.org) using 17 parameters (Ly6G, CD31, CD4, CD8, CCR2, CD19, Ly6c, CD64, CD16/32, CD11b, SiglecF, F4/80, CX3CR1, NK1.1, Epcam, CD103, CD11c). The automated analysis was performed by the FlowSOM algorithm. Hierarchical consensus clustering, with 15 metaclusters, 64 clusters, iterations 10 has been used to generate the SOM (self-organizing map) clusters. The heatmaps were generated using log_2_ ratio of mean intensity by the lowest expressing protein in individual clusters.

### CITRUS analysis

We ran the CITRUS algorithm (Bruggner et al., 2014) using the Cytobank platform (https://www.cytobank.org) to identify clusters and biological signatures for our single cell datasets, which contain six samples (*n* = 9) across two endpoints (naïve and trained). We manually gated singlet viable macrophages (Fig. S5 D) from naïve and trained mice and used the chosen population for CITRUS analysis. To configure the CITRUS clustering algorithm, we chose 28 parameters including active Caspase-3, MHCI, CCR2, CD68, Ly6c, CD64, Arginase-1, CD16/32, CD11b, SiglecF, BrdU, IL-10, F4/80, NOX2, MERTK, TNFα, V-ATPase, CX3CR1, Tim4, CD163, CD206, CD169, CD80, CD86, MHCII, Marco, Lyve1, and CD11c. We set the number of clustered cells as 5,000 events and specified the minimum cluster size percent of interest to be 2%. Markers were transformed before analysis. For the association model selection, we applied the significance analysis of microarrays (SAM)–Correlative association model. We set other parameters as follows: cross-validation folds equals 1, and FDR threshold equals 1%.

### RNA isolation and real-time qPCR

Total RNA from lung AMs was isolated using the RNeasy Plus kit (#74034; Qaigen). After quantification by Nanodrop 1000 (Thermo Fisher Scientific), 500 ng of RNA were reverse-transcribed into complementary DNA using a High-Capacity cDNA Reverse Transcription Kit (#4368814; Thermo Fischer Scientific) according to the manufacturer’s instructions. The cDNA obtained was mixed with FastStart Universal SYBR Green Master (Rox; #4913914001; Millipore Sigma) with specific qPCR primers (Table S3) for qPCR on an ABI Prism 7000 system and analyzed for relative quantification on the ViiA-7 and Quant studio Real-Time PCR System (Thermo Fisher Scientific).

### Bulk RNA-seq

Agilent Bio-analyzer was used to check RNA quality and quantity. All samples showed RNA integrity number >8. RNA-seq libraries were prepared using Illumina mRNA TruSeq kits according to the protocol by Illumina. Library quality and quantity were checked, and the pool of libraries was sequenced using an Illumina HiSeq4000 and Illumina reagents. The data are available via Gene Expression Omnibus (GEO) accession no. GSE231219 and its linked datasets.

### RNA-seq analysis

Raw reads were aligned to reference genome mm10 using STAR (Dobin et al., 2013). ENSEMBL genes were quantified using Feature Counts (Liao et al., 2014). Differential expression statistics (fold-change and P value) were computed using edgeR (McCarthy et al., 2012; Robinson et al., 2010) using the generalized linear model (GLM) capability to correct for a batch effect between groups of replicates. P values were adjusted for multiple testing using the FDR correction of Benjamini and Hochberg. Significant genes were determined based on an FDR threshold of 5% (0.05), and up- and downregulated genes from 7-d LPS treatment were obtained based on either LPS treatment at 72 h or no LPS at 72 h. GO biological process pathways were obtained from MSigDB, and enrichment statistics for up- and downregulated gene lists at 72 h LPS and no-72 h LPS were computed using Fisher’s exact test. Genome-wide binding sites for KLF4 and NF-κB for mm10 were downloaded from the Gene Transcription Regulation Database (Yevshin et al., 2017) and were intersected with gene promoters, defined as 2,000 bp upstream to 1,000 bp downstream of the TSS. Intersections between differentially expressed genes and KLF4- or NF-κB–binding sites were obtained, and enrichment statistics were computed using Fisher’s exact test.

### ATAC-seq

ATAC-seq library preparation was performed on FACS-sorted mouse AMs (200,000 cells per sample) using the ATAC-seq kit from Active motif (#13150) according to the manufacturer’s protocol. The purified DNA was amplified for 10 cycles using indexed primers and size-selected using SPRI bead solution. A quality control (QC) was performed to verify the size distribution of the PCR-enriched library fragments. To this end, aliquots of the DNA libraries were analyzed on a TapeStation, and quantification of the libraries was done using the Qubit. The bar-corded amplicons from ATAC-Library preparation were sequenced in a NovaSeq SP (Illumina) under a 2 × 150 pair-ended format. Reads were quality filtered according to the standard Illumina pipeline, de-multiplexed, and fastq files were generated (GEO accession no. GSE231219).

### ATAC-seq data processing and alignment

ATAC-seq libraries have an average of 39.6 million paired reads per sample, with a total of 158.4 million sequencing reads for four samples. Adapter sequences were identified using fastqc (http://www.bioinformatics.babraham.ac.uk/projects/fastqc/), and we applied TrimGalore (https://www.bioinformatics.babraham.ac.uk/projects/trim_galore/) to remove the adapters for each sample. Trimmed reads were aligned to the mouse mm10 reference genome by using Bowtie2 (v22; Langmead and Salzberg, 2012). We filtered reads by only keeping the unique mapping paired reads with a quality score equal to or more than 30, with alignmentSieve using the option setting of --minMappingQuality 30. PCR duplicated reads were removed by Picard (http://broadinstitute.github.io/picard/). We also filtered out mitochondrial DNA by using SAMtools (Danecek et al., 2021). For Tn5 offset adjustment, the start sites of all the reads were adjusted to represent the center of the transposon binding event. We added 4 bp to plus-strand insertion and −5 bp to minus-strand insertion.

### ATAC-seq peak calling

MACS2 (Zhang et al., 2008) was applied for peak calling with default parameters and peaks filtered based on a FDR <0.01. Irreproducibility discovery rate (I5) framework was applied to handle biological replicates. We kept peaks with an irreproducibility discovery rate score equal to or more than 540 to ensure peaks were consistent among biological replicates. BEDTools (Quinlan and Hall, 2010) were used to merge the peaks across samples into consensus peak regions. We also then filtered out the consensus peak regions using mm10 blacklist from ENCODE. To quantify the number of reads that are located within consensus peak regions, we applied the *featurecounts* function from the Rsubread (Liao et al., 2019) R package.

### ATAC-seq data QC

We assessed the TSS enrichment after alignment using the *computeMatrix* function from deepTools (Ramírez et al., 2016). TSS heatmap was plotted using *plotHeatmap* function. Then we plotted the fragment size distribution from the aligned BAM files using *fragSizeDist* function from ATACSeqQC R package (Yu et al., 2015). For each sample, we observed TSS enrichment across multiple genes and multimodal fragment size distribution as seen in Fig. S4, A and B.

### ATAC-seq data analysis

The DESeq2 (Love et al., 2014) R package was applied to identify DARs. Read counts piled within the consensus peak regions were normalized and log-transformed. Significant DARs were identified using FDR <0.05. To functionally annotate the DARs’ genomic location, we applied the annotate Peak() function from ChIPseeker R package (Yu et al., 2015).

### TF motif enrichment analysis

For TF motif enrichment analysis, first, we collected TF motif information from the JASPAR 2022 database (Castro-Mondragon et al., 2022). Then we extracted promoter DARs regions that have more accessibility in the trained group than the naïve group. Using this region’s bed file, we applied two TF motif enrichment approaches, Homer (Heinz et al., 2010) and MEME suit (Bailey et al., 2015). The parameter setting for Homer is as follows: -p 12 -mask -mknown Jaspar_mm_core_homer.motifs -mcheck Jaspar_mm_core_homer.motifs. The parameter setting for the MEME suite is as follows: sea --verbosity 1 --oc. --thresh 10.0 --align center.

### Phagocytosis assay

To measure phagocytosis of *E. coli*–GFP by AMs, cells were isolated from lavage as outlined before. A total of 1 × 10^5^ AMs were incubated with bacteria (multiplicity of infection, 1:50) for 1 h in the dark at 37°C and 5% CO_2_. Cells were washed three times with PBS and then stained with appropriate antibodies for 30 min at 4°C. Samples were analyzed by flow cytometry and confocal microscopy.

### Efferocytosis ex vivo

AMs were harvested after BAL and cells are plated in DMEM (with 10% FBS) for 2 h. Bone marrow neutrophils were isolated by Percoll density gradient. Cells were first labeled with CMTPX-red cell tracker (#C34552; Invitrogen) according to the manufacturer’s instruction and were induced to apoptosis by UV irradiation (Zhang et al., 2019b). After 2 h, early apoptotic neutrophils were verified with annexin V positivity and plated with AMs at a ratio of 5:1 (neutrophil:AMs). Non-engulfed neutrophils were removed from adherent AMs after 2 h of coculture. The uptake of dead neutrophils was measured by confocal microscopy and flow cytometry analysis.

### Immunofluorescence and confocal microscopy

Isolated cells from lavage were plated in a glass-bottom dish. After 2 h, the cells were washed with PBS followed by a 10-min Fc block. Then surface staining was done with anti-SiglecF-APC (BioLegend) tagged antibody. After 30 min, cells were fixed with 4% paraformaldehyde (#P6148; Sigma-Aldrich) and permeabilized with 0.05% Triton X-100 (#BP151–100; Thermo Fisher Scientific). After a wash with PBS/Tween-20 (#BP337–100; Thermo Fisher Scientific), cells were blocked with 5% BSA (Sigma-Aldrich) in PBS/Tw-20 for 1 h at room temperature. Afterward, cells were incubated with anti-KLF4 primary antibodies (#AF3158; R & D system) and incubated overnight at 4°C. The next day, slides were washed and incubated with the fluorescence-conjugated secondary antibody (AF546 donkey anti-goat 1:300, #A-11056; Invitrogen). Images were taken with a confocal microscope LSM880 (Zeiss) and analyzed by ImageJ.

### Chromatin accessibility

*KLF4* gene promoter accessibility was determined using the EpiQuik Chromatin Accessibility Assay Kit (Epigentek:P-1047-48) following the protocol provided by the manufacturer. Chromatin was isolated from AMs isolated from naïve and trained mice (*n* = 3/group) and treated with a nuclease mix (Nse). Isolated DNA was then amplified using quantitative PCR and gene-specific primers for the *KLF4* promoter (listed in Table S2). Control mouse primers specific for euchromatin regions were provided to determine the successful digestion of the chromatin. The fold enrichment (FE) was calculated by the ratio of amplification efficiency of the Nse-treated DNA sample vs. sample that was not treated with a nuclease (no-Nse) for each group by using the formula $\text{FE}=2^{\left(\text{Nse CT}-\text{no-Nse CT}\right)}\times 100\%$.

### Methylation-specific qPCR

Tissue DNAs were extracted using PureLink genomic DNA Mini Kit (K1820-01; Invitrogen) following the manufacturer’s instructions. Extracted genomic DNA (500 ng) was treated with BisulFlash DNA Modification Kit (P-1026-050; Epigentek) according to the manufacturer’s protocol. 1 μl of 20 μl eluate was used for real time methylation-specific qPCR using Methylamp MS-qPCR Fast Kit (P-1028; Epigentek) according to the manufacturer’s instructions. Methylation-specific primers for *KLF4* promoter (Table S2) were designed using Methprimer (http://www.urogene.org/methprimer/). Percentage of methylation in each sample was calculated by the following equation: $\%\text{Methylation}=100/\left[1+2^{\text{DCt}(\text{meth}-\text{unmeth})}\right]\%$.

### TF binding prediction

The mouse-*MERTK* promoter was retrieved from the Eucaryotic Promoter Database and analyzed for the presence of KLF4-binding motif (GC-rich DNA sequences with a consensus core sequence CACCC) by using the Eucaryotic Promoter Database and JASPAR. Genomic sequences spanning the promoter of *MERTK* gene were analyzed using CLUSTALW to identify conserved regions (National Center for Biotechnology Information accessions: NM_022943, NM_006343, NC_000068, NC_041766).

### ChIP

AMs were isolated by FACS and were crosslinked with the treatment of 1% formaldehyde followed by cell lysis. The nuclei portion was sonicated to fragment the DNA by using S220 Focused-ultrasonicator (Covaris). Further procedures were carried out using the Chromatin Immunoprecipitation (ChIP) Assay Kit (#17-295; Millipore Sigma) according to the manufacturer’s protocol. 5 μg of anti-KLF4 antibody (#AF3158; R&D Systems) or an equal amount of goat IgG was used for respective ChIP assay. DNA was recovered from antibody–protein–DNA complexes by phenol/chloroform extraction and ethanol precipitation. The qPCR following ChIP was performed with the specific primer sets as listed (Table S3). Normal rabbit IgG was used as a negative antibody control. For the negative control region, a mouse negative control primer set from Active Motif (#71011) was used.

### shRNA-liposome preparation

We purchased the four unique 29mer shRNA constructs in a lentiviral GFP vector (#TL501193; Origene). To prepare the liposome, dimethyldioctadecylammonium bromide (#D2779; Sigma-Aldrich) was dissolved in chloroform (#C606SK-1; Fisher Chemical) in a 1:1 M ratio with cholesterol (#228111; Millipore). In a Rotavapor (#R-300; Buchi), chloroform was evaporated at 37°C, 100 rpm for 20–30 min. The dried lipid film was resuspended in 5% dextrose (#D16–500; Thermo Fisher Scientific) in water and further sonicated in Branson 2510 Ultrasonic Cleaner (#Z244910; Sigma-Aldrich) for 30 min, followed by 0.45 μm filtration. Liposome–shRNA (a cocktail of four shRNAs) complex was prepared with a concentration of 50 μg DNA/100 μg liposome per mouse (Liu et al., 2019). The physical characterization of the prepared liposome-shRNA complex was performed by measuring the hydrodynamic size and the surface zeta potential by using Malvern Zetasizer. The mice were fully anesthetized with ketamine/xylazine and the liposomes were i.t. injected.

### Adoptive transfer

AMs were enriched by positive magnetic selection using anti-CD11c (Miltenyi Biotech). The positively selected fraction was collected after passing through the LS column (Miltenyi Biotech) on an MACS magnetic separator (Miltenyi Biotech) and plated with DMEM with 10% fetal calf serum, *L*-glutamine, penicillin, and streptomycin. AMs were kept for 2 h to adhere and washed with warm media. The purity of the cells was confirmed by flow cytometry. From this, 2 × 10^6^ macrophages were transferred per recipient. The adoptive transfer was performed in 50 μl of sterile PBS by i.t. delivery into mice anesthetized with ketamine/xylazine. Before transfer, host AMs were not depleted to avoid bystander inflammatory response (Aegerter et al., 2020).

### Lung histological analysis

For histological analysis of the lung, after perfusion, the lung tissues were fixed using 10% buffered formaldehyde and processed for the paraffin section. The histological sectioning and staining for H&E were done by Research Histology Core, University of Illinois, Chicago. Images were taken by using an Olympus BX51/DP72 microscope.

### ELISA from bronchoalveolar macrophage

The trachea was dissected and cannulated with an 18-gauge catheter. Lungs were lavaged single time with 1 ml PBS supplemented with protease inhibitor. Collected samples were centrifuged at 2,000 rpm for 5 min, and the supernatant was collected for ELISA. The levels of TNFα and IL-10 were measured using an ELISA kit (LEGEND MAX Mouse IL-10 ELISA Kit Cat #431417, LEGEND MAX Mouse TNF-α ELISA Kit Cat #430907; BioLegend) according to the manufacturer’s instructions.

### Statistical analysis

Statistical analyses were performed using Graph Pad Prism, version 9.0, and with corrections for multiple comparisons whenever appropriate. Data are displayed as mean ± SEM. Statistical significance was evaluated as indicated in the figure legends and is represented by the following scheme: *P ≤ 0.05, **P ≤ 0.01, ***P ≤ 0.001, ****P ≤ 0.0001.

### Online supplemental material

Fig. S1 shows the phenotypic characteristics of in vivo generated trained AM. Fig. S2 shows the shifts in cell populations of naïve vs. trained AMs in response to the LPS challenge. Fig. S3 demonstrates the phenotypic characteristics of lung macrophage sub-populations in trained mice. Fig. S4 shows differential chromatin accessibility in naïve vs. trained AM as analyzed by ATAC-seq. It also illustrates the conservation of the KLF4-binding sites in MERTK promoter across different species. Fig. S5 visualizes the breeding and treatment schematic to generate macrophage-specific deletion of KLF4 in mice. Tables S1, S2, S3, and S4 contains the lists of antibodies for flow cytometry and mass cytometry, a list of primers, and a list of genes represented in the heatmap for transcriptomic analysis.

## Supplementary Material

Table S1 lists the sources of antibodies used for flow cytometry along with their dilution ratios.

Table S2 lists the antibodies used for CyTOF assay in this study as well as their dilution ratios.

Table S3 lists the sequences for primers used in qPCR, chromatin accessibility assay, MSP-qPCR, and ChIP-qPCR.

Table S4 lists differentially expressed genes of naïve vs. trained AM shown in the Fig. 1 H heatmap.

## Data Availability

RNA-seq (Fig. 1) and ATAC-seq (Fig. 6) data generated for this study are publicly available under GEO accession no. GSE231219. All the other data underlying the research are available in the article itself and its supplementary material.

## References

[bib1] Aegerter, H., J. Kulikauskaite, S. Crotta, H. Patel, G. Kelly, E.M. Hessel, M. Mack, S. Beinke, and A. Wack. 2020. Influenza-induced monocyte-derived alveolar macrophages confer prolonged antibacterial protection. Nat. Immunol. 21:145–157. 10.1038/s41590-019-0568-x 31932810 PMC6983324

[bib2] Akalu, Y.T., M.E. Mercau, M. Ansems, L.D. Hughes, J. Nevin, E.J. Alberto, X.N. Liu, L.Z. He, D. Alvarado, T. Keler, . 2022. Tissue-specific modifier alleles determine Mertk loss-of-function traits. Elife. 11:e80530. 10.7554/eLife.80530 35969037 PMC9433089

[bib3] Amir, el-A.D., K.L. Davis, M.D. Tadmor, E.F. Simonds, J.H. Levine, S.C. Bendall, D.K. Shenfeld, S. Krishnaswamy, G.P. Nolan, and D. Pe’er. 2013. viSNE enables visualization of high dimensional single-cell data and reveals phenotypic heterogeneity of leukemia. Nat. Biotechnol. 31:545–552. 10.1038/nbt.2594 23685480 PMC4076922

[bib4] Ampomah, P.B., B. Cai, S.R. Sukka, B.D. Gerlach, A. Yurdagul Jr, X. Wang, G. Kuriakose, L.N.F. Darville, Y. Sun, S. Sidoli, . 2022. Macrophages use apoptotic cell-derived methionine and DNMT3A during efferocytosis to promote tissue resolution. Nat. Metab. 4:444–457. 10.1038/s42255-022-00551-7 35361955 PMC9050866

[bib5] Aran, D., A.P. Looney, L. Liu, E. Wu, V. Fong, A. Hsu, S. Chak, R.P. Naikawadi, P.J. Wolters, A.R. Abate, . 2019. Reference-based analysis of lung single-cell sequencing reveals a transitional profibrotic macrophage. Nat. Immunol. 20:163–172. 10.1038/s41590-018-0276-y 30643263 PMC6340744

[bib6] Bailey, T.L., J. Johnson, C.E. Grant, and W.S. Noble. 2015. The MEME suite. Nucleic Acids Res. 43:W39–W49. 10.1093/nar/gkv416 25953851 PMC4489269

[bib7] Bekkering, S., R.J.W. Arts, B. Novakovic, I. Kourtzelis, C.D.C.C. van der Heijden, Y. Li, C.D. Popa, R. Ter Horst, J. van Tuijl, R.T. Netea-Maier, . 2018. Metabolic induction of trained immunity through the mevalonate pathway. Cell. 172:135–146.e9. 10.1016/j.cell.2017.11.025 29328908

[bib8] Bonnardel, J., W. T’Jonck, D. Gaublomme, R. Browaeys, C.L. Scott, L. Martens, B. Vanneste, S. De Prijck, S.A. Nedospasov, A. Kremer, . 2019. Stellate cells, hepatocytes, and endothelial cells imprint the kupffer cell identity on monocytes colonizing the liver macrophage niche. Immunity. 51:638–654.e9. 10.1016/j.immuni.2019.08.017 31561945 PMC6876284

[bib9] Boring, L., J. Gosling, S.W. Chensue, S.L. Kunkel, R.V. Farese Jr., H.E. Broxmeyer, and I.F. Charo. 1997. Impaired monocyte migration and reduced type 1 (Th1) cytokine responses in C-C chemokine receptor 2 knockout mice. J. Clin. Invest. 100:2552–2561. 10.1172/JCI119798 9366570 PMC508456

[bib10] Bosurgi, L., Y.G. Cao, M. Cabeza-Cabrerizo, A. Tucci, L.D. Hughes, Y. Kong, J.S. Weinstein, P. Licona-Limon, E.T. Schmid, F. Pelorosso, . 2017. Macrophage function in tissue repair and remodeling requires IL-4 or IL-13 with apoptotic cells. Science. 356:1072–1076. 10.1126/science.aai8132 28495875 PMC5556699

[bib11] Bouchet-Delbos, L., A. Even, E. Varey, S. Saïagh, S. Bercegeay, C. Braudeau, B. Dréno, G. Blancho, R. Josien, M.C. Cuturi, and A. Moreau. 2021. Preclinical assessment of autologous tolerogenic dendritic cells from end-stage renal disease patients. Transplantation. 105:832–841. 10.1097/TP.0000000000003315 32433241

[bib12] Bruggner, R.V., B. Bodenmiller, D.L. Dill, R.J. Tibshirani, and G.P. Nolan. 2014. Automated identification of stratifying signatures in cellular subpopulations. Proc. Natl. Acad. Sci. USA. 111:E2770–E2777. 10.1073/pnas.1408792111 24979804 PMC4084463

[bib13] Cai, B., E.B. Thorp, A.C. Doran, B.E. Sansbury, M.J.A.P. Daemen, B. Dorweiler, M. Spite, G. Fredman, and I. Tabas. 2017. MerTK receptor cleavage promotes plaque necrosis and defective resolution in atherosclerosis. J. Clin. Invest. 127:564–568. 10.1172/JCI90520 28067670 PMC5272169

[bib105] Cai, B., C. Kasikara, A.C. Doran, R. Ramakrishnan, R.B. Birge, and I. Tabas. 2018. MerTK signaling in macrophages promotes the synthesis of inflammation resolution mediators by suppressing CaMKII activity. Sci. Signal. 11:eaar3721. 10.1126/scisignal.aar3721 30254055 PMC6171110

[bib14] Castro-Mondragon, J.A., R. Riudavets-Puig, I. Rauluseviciute, R.B. Lemma, L. Turchi, R. Blanc-Mathieu, J. Lucas, P. Boddie, A. Khan, N. Manosalva Pérez, . 2022. JASPAR 2022: The 9th release of the open-access database of transcription factor binding profiles. Nucleic Acids Res. 50:D165–D173. 10.1093/nar/gkab1113 34850907 PMC8728201

[bib15] Chakarov, S., H.Y. Lim, L. Tan, S.Y. Lim, P. See, J. Lum, X.M. Zhang, S. Foo, S. Nakamizo, K. Duan, . 2019. Two distinct interstitial macrophage populations coexist across tissues in specific subtissular niches. Science. 363:eaau0964. 10.1126/science.aau0964 30872492

[bib16] Chang, C.F., B.A. Goods, M.H. Askenase, M.D. Hammond, S.C. Renfroe, A.F. Steinschneider, M.J. Landreneau, Y. Ai, H.E. Beatty, L.H.A. da Costa, . 2018. Erythrocyte efferocytosis modulates macrophages towards recovery after intracerebral hemorrhage. J. Clin. Invest. 128:607–624. 10.1172/JCI95612 29251628 PMC5785262

[bib17] Chen, Y., J. Zhang, W. Cui, and R.L. Silverstein. 2022. CD36, a signaling receptor and fatty acid transporter that regulates immune cell metabolism and fate. J. Exp. Med. 219:e20211314. 10.1084/jem.20211314 35438721 PMC9022290

[bib18] Cheng, S.C., J. Quintin, R.A. Cramer, K.M. Shepardson, S. Saeed, V. Kumar, E.J. Giamarellos-Bourboulis, J.H.A. Martens, N.A. Rao, A. Aghajanirefah, . 2014. mTOR- and HIF-1α-mediated aerobic glycolysis as metabolic basis for trained immunity. Science. 345:1250684. 10.1126/science.1250684 25258083 PMC4226238

[bib19] Cowan, C.E., E.E. Kohler, T.A. Dugan, M.K. Mirza, A.B. Malik, and K.K. Wary. 2010. Kruppel-like factor-4 transcriptionally regulates VE-cadherin expression and endothelial barrier function. Circ. Res. 107:959–966. 10.1161/CIRCRESAHA.110.219592 20724706 PMC3018700

[bib20] Cupovic, J., S.S. Ring, L. Onder, J.M. Colston, M. Lütge, H.W. Cheng, A. De Martin, N.M. Provine, L. Flatz, A. Oxenius, . 2021. Adenovirus vector vaccination reprograms pulmonary fibroblastic niches to support protective inflating memory CD8^+^ T cells. Nat. Immunol. 22:1042–1051. 10.1038/s41590-021-00969-3 34267375 PMC7611414

[bib21] Dagvadorj, J., K. Shimada, S. Chen, H.D. Jones, G. Tumurkhuu, W. Zhang, K.A. Wawrowsky, T.R. Crother, and M. Arditi. 2015. Lipopolysaccharide induces alveolar macrophage necrosis via CD14 and the P2X7 receptor leading to interleukin-1α release. Immunity. 42:640–653. 10.1016/j.immuni.2015.03.007 25862090 PMC4423803

[bib22] Danecek, P., J.K. Bonfield, J. Liddle, J. Marshall, V. Ohan, M.O. Pollard, A. Whitwham, T. Keane, S.A. McCarthy, R.M. Davies, and H. Li. 2021. Twelve years of SAMtools and BCFtools. Gigascience. 10:giab008. 10.1093/gigascience/giab008 33590861 PMC7931819

[bib23] de Laval, B., J. Maurizio, P.K. Kandalla, G. Brisou, L. Simonnet, C. Huber, G. Gimenez, O. Matcovitch-Natan, S. Reinhardt, E. David, . 2020. C/EBPβ-Dependent epigenetic memory induces trained immunity in hematopoietic stem cells. Cell Stem Cell. 26:657–674.e8. 10.1016/j.stem.2020.01.017 32169166

[bib24] Dhaliwal, N.K., L.E. Abatti, and J.A. Mitchell. 2019. KLF4 protein stability regulated by interaction with pluripotency transcription factors overrides transcriptional control. Genes Dev. 33:1069–1082. 10.1101/gad.324319.119 31221664 PMC6672055

[bib25] Dhaliwal, N.K., K. Miri, S. Davidson, H. Tamim El Jarkass, and J.A. Mitchell. 2018. KLF4 nuclear export requires ERK activation and initiates exit from naive pluripotency. Stem Cell Rep. 10:1308–1323. 10.1016/j.stemcr.2018.02.007 PMC6000723 29526737

[bib26] Divangahi, M., P. Aaby, S.A. Khader, L.B. Barreiro, S. Bekkering, T. Chavakis, R. van Crevel, N. Curtis, A.R. DiNardo, J. Dominguez-Andres, . 2021. Trained immunity, tolerance, priming and differentiation: Distinct immunological processes. Nat. Immunol. 22:2–6. 10.1038/s41590-020-00845-6 33293712 PMC8020292

[bib27] Dobin, A., C.A. Davis, F. Schlesinger, J. Drenkow, C. Zaleski, S. Jha, P. Batut, M. Chaisson, and T.R. Gingeras. 2013. STAR: Ultrafast universal RNA-seq aligner. Bioinformatics. 29:15–21. 10.1093/bioinformatics/bts635 23104886 PMC3530905

[bib28] Elliott, M.R., K.M. Koster, and P.S. Murphy. 2017. Efferocytosis signaling in the regulation of macrophage inflammatory responses. J. Immunol. 198:1387–1394. 10.4049/jimmunol.1601520 28167649 PMC5301545

[bib29] Evavold, C.L., I. Hafner-Bratkovič, P. Devant, J.M. D’Andrea, E.M. Ngwa, E. Boršić, J.G. Doench, M.W. LaFleur, A.H. Sharpe, J.R. Thiagarajah, and J.C. Kagan. 2021. Control of gasdermin D oligomerization and pyroptosis by the Ragulator-Rag-mTORC1 pathway. Cell. 184:4495–4511.e19. 10.1016/j.cell.2021.06.028 34289345 PMC8380731

[bib30] Fan, Y., H. Lu, W. Liang, W. Hu, J. Zhang, and Y.E. Chen. 2017. Krüppel-like factors and vascular wall homeostasis. J. Mol. Cell Biol. 9:352–363. 10.1093/jmcb/mjx037 28992202 PMC5907833

[bib31] Fanucchi, S., E.T. Fok, E. Dalla, Y. Shibayama, K. Börner, E.Y. Chang, S. Stoychev, M. Imakaev, D. Grimm, K.C. Wang, . 2019. Immune genes are primed for robust transcription by proximal long noncoding RNAs located in nuclear compartments. Nat. Genet. 51:138–150. 10.1038/s41588-018-0298-2 30531872

[bib32] Feuerstein, R., A.J. Forde, F. Lohrmann, J. Kolter, N.J. Ramirez, J. Zimmermann, M. Gomez de Aguero, and P. Henneke. 2020. Resident macrophages acquire innate immune memory in staphylococcal skin infection. Elife. 9:e55602. 10.7554/eLife.55602 32639232 PMC7343389

[bib33] Fossati, G., R.J. Moots, R.C. Bucknall, and S.W. Edwards. 2002. Differential role of neutrophil Fcgamma receptor IIIB (CD16) in phagocytosis, bacterial killing, and responses to immune complexes. Arthritis Rheum. 46:1351–1361. 10.1002/art.10230 12115243

[bib34] Giannoukakis, N., B. Phillips, D. Finegold, J. Harnaha, and M. Trucco. 2011. Phase I (safety) study of autologous tolerogenic dendritic cells in type 1 diabetic patients. Diabetes Care. 34:2026–2032. 10.2337/dc11-0472 21680720 PMC3161299

[bib35] Ginhoux, F., and M. Guilliams. 2016. Tissue-resident macrophage ontogeny and homeostasis. Immunity. 44:439–449. 10.1016/j.immuni.2016.02.024 26982352

[bib36] Ginhoux, F., and S. Jung. 2014. Monocytes and macrophages: Developmental pathways and tissue homeostasis. Nat. Rev. Immunol. 14:392–404. 10.1038/nri3671 24854589

[bib37] Grandi, F.C., R. Baskar, P. Smeriglio, S. Murkherjee, P.F. Indelli, D.F. Amanatullah, S. Goodman, C. Chu, S. Bendall, and N. Bhutani. 2020. Single-cell mass cytometry reveals cross-talk between inflammation-dampening and inflammation-amplifying cells in osteoarthritic cartilage. Sci. Adv. 6:eaay5352. 10.1126/sciadv.aay5352 32201724 PMC7069698

[bib38] Guilliams, M., I. De Kleer, S. Henri, S. Post, L. Vanhoutte, S. De Prijck, K. Deswarte, B. Malissen, H. Hammad, and B.N. Lambrecht. 2013. Alveolar macrophages develop from fetal monocytes that differentiate into long-lived cells in the first week of life via GM-CSF. J. Exp. Med. 210:1977–1992. 10.1084/jem.20131199 24043763 PMC3782041

[bib39] Guillon, A., E.I. Arafa, K.A. Barker, A.C. Belkina, I. Martin, A.T. Shenoy, A.K. Wooten, C. Lyon De Ana, A. Dai, A. Labadorf, . 2020. Pneumonia recovery reprograms the alveolar macrophage pool. JCI Insight. 5:e133042. 10.1172/jci.insight.133042 31990682 PMC7101156

[bib40] Hasenberg, A., M. Hasenberg, L. Mann, F. Neumann, L. Borkenstein, M. Stecher, A. Kraus, D.R. Engel, A. Klingberg, P. Seddigh, . 2015. Catchup: A mouse model for imaging-based tracking and modulation of neutrophil granulocytes. Nat. Methods. 12:445–452. 10.1038/nmeth.3322 25775045

[bib41] He, W., C.J. Chen, C.E. Mullarkey, J.R. Hamilton, C.K. Wong, P.E. Leon, M.B. Uccellini, V. Chromikova, C. Henry, K.W. Hoffman, . 2017. Alveolar macrophages are critical for broadly-reactive antibody-mediated protection against influenza A virus in mice. Nat. Commun. 8:846. 10.1038/s41467-017-00928-3 29018261 PMC5635038

[bib42] Heinz, S., C. Benner, N. Spann, E. Bertolino, Y.C. Lin, P. Laslo, J.X. Cheng, C. Murre, H. Singh, and C.K. Glass. 2010. Simple combinations of lineage-determining transcription factors prime cis-regulatory elements required for macrophage and B cell identities. Mol. Cell. 38:576–589. 10.1016/j.molcel.2010.05.004 20513432 PMC2898526

[bib43] Hoyer, F.F., K. Naxerova, M.J. Schloss, M. Hulsmans, A.V. Nair, P. Dutta, D.M. Calcagno, F. Herisson, A. Anzai, Y. Sun, . 2019. Tissue-specific macrophage responses to remote injury impact the outcome of subsequent local immune challenge. Immunity. 51:899–914.e7. 10.1016/j.immuni.2019.10.010 31732166 PMC6892583

[bib44] Ishii, M., H. Wen, C.A.S. Corsa, T. Liu, A.L. Coelho, R.M. Allen, W.F. Carson IV, K.A. Cavassani, X. Li, N.W. Lukacs, . 2009. Epigenetic regulation of the alternatively activated macrophage phenotype. Blood. 114:3244–3254. 10.1182/blood-2009-04-217620 19567879 PMC2759649

[bib45] Jambusaria, A., Z. Hong, L. Zhang, S. Srivastava, A. Jana, P.T. Toth, Y. Dai, A.B. Malik, and J. Rehman. 2020. Endothelial heterogeneity across distinct vascular beds during homeostasis and inflammation. Elife. 9:e51413. 10.7554/eLife.51413 31944177 PMC7002042

[bib46] Janssen, W.J., L. Barthel, A. Muldrow, R.E. Oberley-Deegan, M.T. Kearns, C. Jakubzick, and P.M. Henson. 2011. Fas determines differential fates of resident and recruited macrophages during resolution of acute lung injury. Am. J. Respir. Crit. Care Med. 184:547–560. 10.1164/rccm.201011-1891OC 21471090 PMC3175550

[bib47] Kapoor, N., J. Niu, Y. Saad, S. Kumar, T. Sirakova, E. Becerra, X. Li, and P.E. Kolattukudy. 2015. Transcription factors STAT6 and KLF4 implement macrophage polarization via the dual catalytic powers of MCPIP. J. Immunol. 194:6011–6023. 10.4049/jimmunol.1402797 25934862 PMC4458412

[bib48] Kaufmann, E., J. Sanz, J.L. Dunn, N. Khan, L.E. Mendonça, A. Pacis, F. Tzelepis, E. Pernet, A. Dumaine, J.C. Grenier, . 2018. BCG educates hematopoietic stem cells to generate protective innate immunity against tuberculosis. Cell. 172:176–190.e19. 10.1016/j.cell.2017.12.031 29328912

[bib49] Khan, N., J. Downey, J. Sanz, E. Kaufmann, B. Blankenhaus, A. Pacis, E. Pernet, E. Ahmed, S. Cardoso, A. Nijnik, . 2020. *M. tuberculosis* reprograms hematopoietic stem cells to limit myelopoiesis and impair trained immunity. Cell. 183:752–770.e22. 10.1016/j.cell.2020.09.062 33125891 PMC7599081

[bib50] Korkmaz, F.T., A.T. Shenoy, E.M. Symer, L.A. Baird, C.V. Odom, E.I. Arafa, E.L. Dimbo, E. Na, W. Molina-Arocho, M. Brudner, . 2022. Lectin-like oxidized low-density lipoprotein receptor 1 attenuates pneumonia-induced lung injury. JCI Insight. 7:e149955. 10.1172/jci.insight.149955 36264633 PMC9746901

[bib51] Lambrecht, B.N. 2006. Alveolar macrophage in the driver’s seat. Immunity. 24:366–368. 10.1016/j.immuni.2006.03.008 16618595

[bib52] Langmead, B., and S.L. Salzberg. 2012. Fast gapped-read alignment with Bowtie 2. Nat. Methods. 9:357–359. 10.1038/nmeth.1923 22388286 PMC3322381

[bib53] Lau, C.M., N.M. Adams, C.D. Geary, O.E. Weizman, M. Rapp, Y. Pritykin, C.S. Leslie, and J.C. Sun. 2018. Epigenetic control of innate and adaptive immune memory. Nat. Immunol. 19:963–972. 10.1038/s41590-018-0176-1 30082830 PMC6225771

[bib54] Lavin, Y., D. Winter, R. Blecher-Gonen, E. David, H. Keren-Shaul, M. Merad, S. Jung, and I. Amit. 2014. Tissue-resident macrophage enhancer landscapes are shaped by the local microenvironment. Cell. 159:1312–1326. 10.1016/j.cell.2014.11.018 25480296 PMC4437213

[bib55] Li, Z., X. Xu, X. Feng, and P.M. Murphy. 2016. The macrophage-depleting agent clodronate promotes durable hematopoietic chimerism and donor-specific skin allograft tolerance in mice. Sci. Rep. 6:22143. 10.1038/srep22143 26917238 PMC4768260

[bib56] Liao, X., N. Sharma, F. Kapadia, G. Zhou, Y. Lu, H. Hong, K. Paruchuri, G.H. Mahabeleshwar, E. Dalmas, N. Venteclef, . 2011. Krüppel-like factor 4 regulates macrophage polarization. J. Clin. Invest. 121:2736–2749. 10.1172/JCI45444 21670502 PMC3223832

[bib57] Liao, Y., G.K. Smyth, and W. Shi. 2014. featureCounts: An efficient general purpose program for assigning sequence reads to genomic features. Bioinformatics. 30:923–930. 10.1093/bioinformatics/btt656 24227677

[bib58] Liao, Y., G.K. Smyth, and W. Shi. 2019. The R package Rsubread is easier, faster, cheaper and better for alignment and quantification of RNA sequencing reads. Nucleic Acids Res. 47:e47. 10.1093/nar/gkz114 30783653 PMC6486549

[bib59] Liu, M., L. Zhang, G. Marsboom, A. Jambusaria, S. Xiong, P.T. Toth, E.V. Benevolenskaya, J. Rehman, and A.B. Malik. 2019. Sox17 is required for endothelial regeneration following inflammation-induced vascular injury. Nat. Commun. 10:2126. 10.1038/s41467-019-10134-y 31073164 PMC6509327

[bib60] Lou, G., W. Hu, Z. Wu, H. Xu, H. Yao, Y. Wang, Q. Huang, B. Wang, L. Wen, D. Gong, . 2020. Tanshinone II A attenuates vascular remodeling through klf4 mediated smooth muscle cell phenotypic switching. Sci. Rep. 10:13858. 10.1038/s41598-020-70887-1 32807822 PMC7431534

[bib61] Love, M.I., W. Huber, and S. Anders. 2014. Moderated estimation of fold change and dispersion for RNA-seq data with DESeq2. Genome Biol. 15:550. 10.1186/s13059-014-0550-8 25516281 PMC4302049

[bib62] McCarthy, D.J., Y. Chen, and G.K. Smyth. 2012. Differential expression analysis of multifactor RNA-Seq experiments with respect to biological variation. Nucleic Acids Res. 40:4288–4297. 10.1093/nar/gks042 22287627 PMC3378882

[bib63] Medzhitov, R., D.S. Schneider, and M.P. Soares. 2012. Disease tolerance as a defense strategy. Science. 335:936–941. 10.1126/science.1214935 22363001 PMC3564547

[bib64] Mehrotra, P., and K.S. Ravichandran. 2022. Drugging the efferocytosis process: Concepts and opportunities. Nat. Rev. Drug Discov. 21:601–620. 10.1038/s41573-022-00470-y 35650427 PMC9157040

[bib65] Mentrup, T., A.Y. Stumpff-Niggemann, N. Leinung, C. Schlosser, K. Schubert, R. Wehner, A. Tunger, V. Schatz, P. Neubert, A.C. Gradtke, . 2022. Phagosomal signalling of the C-type lectin receptor Dectin-1 is terminated by intramembrane proteolysis. Nat. Commun. 13:1880. 10.1038/s41467-022-29474-3 35388002 PMC8987071

[bib66] Min-Oo, G., and L.L. Lanier. 2014. Cytomegalovirus generates long-lived antigen-specific NK cells with diminished bystander activation to heterologous infection. J. Exp. Med. 211:2669–2680. 10.1084/jem.20141172 25422494 PMC4267234

[bib67] Misharin, A.V., L. Morales-Nebreda, P.A. Reyfman, C.M. Cuda, J.M. Walter, A.C. McQuattie-Pimentel, C.I. Chen, K.R. Anekalla, N. Joshi, K.J.N. Williams, . 2017. Monocyte-derived alveolar macrophages drive lung fibrosis and persist in the lung over the life span. J. Exp. Med. 214:2387–2404. 10.1084/jem.20162152 28694385 PMC5551573

[bib68] Morioka, S., J.S.A. Perry, M.H. Raymond, C.B. Medina, Y. Zhu, L. Zhao, V. Serbulea, S. Onengut-Gumuscu, N. Leitinger, S. Kucenas, . 2018. Efferocytosis induces a novel SLC program to promote glucose uptake and lactate release. Nature. 563:714–718. 10.1038/s41586-018-0735-5 30464343 PMC6331005

[bib69] Netea, M.G., J. Domínguez-Andrés, L.B. Barreiro, T. Chavakis, M. Divangahi, E. Fuchs, L.A.B. Joosten, J.W.M. van der Meer, M.M. Mhlanga, W.J.M. Mulder, . 2020. Defining trained immunity and its role in health and disease. Nat. Rev. Immunol. 20:375–388. 10.1038/s41577-020-0285-6 32132681 PMC7186935

[bib70] Netea, M.G., L.A.B. Joosten, E. Latz, K.H.G. Mills, G. Natoli, H.G. Stunnenberg, L.A.J. O'Neill, and R.J. Xavier. 2016. Trained immunity: A program of innate immune memory in health and disease. Science. 352:aaf1098. 10.1126/science.aaf1098 27102489 PMC5087274

[bib71] Ochocka, N., P. Segit, K.A. Walentynowicz, K. Wojnicki, S. Cyranowski, J. Swatler, J. Mieczkowski, and B. Kaminska. 2021. Single-cell RNA sequencing reveals functional heterogeneity of glioma-associated brain macrophages. Nat. Commun. 12:1151. 10.1038/s41467-021-21407-w 33608526 PMC7895824

[bib72] Quinlan, A.R., and I.M. Hall. 2010. BEDTools: A flexible suite of utilities for comparing genomic features. Bioinformatics. 26:841–842. 10.1093/bioinformatics/btq033 20110278 PMC2832824

[bib73] Quintin, J., S. Saeed, J.H.A. Martens, E.J. Giamarellos-Bourboulis, D.C. Ifrim, C. Logie, L. Jacobs, T. Jansen, B.J. Kullberg, C. Wijmenga, . 2012. Candida albicans infection affords protection against reinfection via functional reprogramming of monocytes. Cell Host Microbe. 12:223–232. 10.1016/j.chom.2012.06.006 22901542 PMC3864037

[bib74] Ramírez, F., D.P. Ryan, B. Grüning, V. Bhardwaj, F. Kilpert, A.S. Richter, S. Heyne, F. Dündar, and T. Manke. 2016. deepTools2: A next generation web server for deep-sequencing data analysis. Nucleic Acids Res. 44:W160–W165. 10.1093/nar/gkw257 27079975 PMC4987876

[bib75] Roberts, A.W., B.L. Lee, J. Deguine, S. John, M.J. Shlomchik, and G.M. Barton. 2017. Tissue-resident macrophages are locally programmed for silent clearance of apoptotic cells. Immunity. 47:913–927.e6. 10.1016/j.immuni.2017.10.006 29150239 PMC5728676

[bib76] Robinson, M.D., D.J. McCarthy, and G.K. Smyth. 2010. edgeR: A bioconductor package for differential expression analysis of digital gene expression data. Bioinformatics. 26:139–140. 10.1093/bioinformatics/btp616 19910308 PMC2796818

[bib106] Rothlin, C.V., and S. Ghosh. 2023. When aging gets on the way of disposal: Senescent cell suppression of efferocytosis. J. Cell Biol. 222:e202212023. 10.1083/jcb.202212023 36602762 PMC9827511

[bib77] Rothlin, C.V., S. Ghosh, E.I. Zuniga, M.B.A. Oldstone, and G. Lemke. 2007. TAM receptors are pleiotropic inhibitors of the innate immune response. Cell. 131:1124–1136. 10.1016/j.cell.2007.10.034 18083102

[bib78] Rowland, B.D., R. Bernards, and D.S. Peeper. 2005. The KLF4 tumour suppressor is a transcriptional repressor of p53 that acts as a context-dependent oncogene. Nat. Cell Biol. 7:1074–1082. 10.1038/ncb1314 16244670

[bib79] Saeed, S., J. Quintin, H.H.D. Kerstens, N.A. Rao, A. Aghajanirefah, F. Matarese, S.C. Cheng, J. Ratter, K. Berentsen, M.A. van der Ent, . 2014. Epigenetic programming of monocyte-to-macrophage differentiation and trained innate immunity. Science. 345:1251086. 10.1126/science.1251086 25258085 PMC4242194

[bib80] Sangwung, P., G. Zhou, L. Nayak, E.R. Chan, S. Kumar, D.W. Kang, R. Zhang, X. Liao, Y. Lu, K. Sugi, . 2017. KLF2 and KLF4 control endothelial identity and vascular integrity. JCI Insight. 2:e91700. 10.1172/jci.insight.91700 28239661 PMC5313061

[bib81] Schneider, C., S.P. Nobs, M. Kurrer, H. Rehrauer, C. Thiele, and M. Kopf. 2014. Induction of the nuclear receptor PPAR-γ by the cytokine GM-CSF is critical for the differentiation of fetal monocytes into alveolar macrophages. Nat. Immunol. 15:1026–1037. 10.1038/ni.3005 25263125

[bib82] Schyns, J., Q. Bai, C. Ruscitti, C. Radermecker, S. De Schepper, S. Chakarov, F. Farnir, D. Pirottin, F. Ginhoux, G. Boeckxstaens, . 2019. Non-classical tissue monocytes and two functionally distinct populations of interstitial macrophages populate the mouse lung. Nat. Commun. 10:3964. 10.1038/s41467-019-11843-0 31481690 PMC6722135

[bib83] Seeley, J.J., R.G. Baker, G. Mohamed, T. Bruns, M.S. Hayden, S.D. Deshmukh, D.E. Freedberg, and S. Ghosh. 2018. Induction of innate immune memory via microRNA targeting of chromatin remodelling factors. Nature. 559:114–119. 10.1038/s41586-018-0253-5 29950719 PMC6044474

[bib84] Shankman, L.S., D. Gomez, O.A. Cherepanova, M. Salmon, G.F. Alencar, R.M. Haskins, P. Swiatlowska, A.A.C. Newman, E.S. Greene, A.C. Straub, . 2015. KLF4-dependent phenotypic modulation of smooth muscle cells has a key role in atherosclerotic plaque pathogenesis. Nat. Med. 21:628–637. 10.1038/nm.3866 25985364 PMC4552085

[bib85] van de Laar, L., W. Saelens, S. De Prijck, L. Martens, C.L. Scott, G. Van Isterdael, E. Hoffmann, R. Beyaert, Y. Saeys, B.N. Lambrecht, and M. Guilliams. 2016. Yolk sac macrophages, fetal liver, and adult monocytes can colonize an empty niche and develop into functional tissue-resident macrophages. Immunity. 44:755–768. 10.1016/j.immuni.2016.02.017 26992565

[bib86] Van Gassen, S., B. Callebaut, M.J. Van Helden, B.N. Lambrecht, P. Demeester, T. Dhaene, and Y. Saeys. 2015. FlowSOM: Using self-organizing maps for visualization and interpretation of cytometry data. Cytometry A. 87:636–645. 10.1002/cyto.a.22625 25573116

[bib87] Vaz de Paula, C.B., M.L.V. de Azevedo, S. Nagashima, A.P.C. Martins, M.A.S. Malaquias, A.F.R.D.S. Miggiolaro, J. da Silva Motta Júnior, G. Avelino, L.A.P. do Carmo, L.B. Carstens, and L. de Noronha. 2020. IL-4/IL-13 remodeling pathway of COVID-19 lung injury. Sci. Rep. 10:18689. 10.1038/s41598-020-75659-5 33122784 PMC7596721

[bib88] Wang, D., H. Zhang, J. Liang, H. Wang, B. Hua, X. Feng, G.S. Gilkeson, D. Farge, S. Shi, and L. Sun. 2018. A long-term follow-up study of allogeneic mesenchymal stem/stromal cell transplantation in patients with drug-resistant systemic lupus erythematosus. Stem Cell Rep. 10:933–941. 10.1016/j.stemcr.2018.01.029 PMC5918528 29478901

[bib89] Wang, Z., X. Liu, F. Cao, J.A. Bellanti, J. Zhou, and S.G. Zheng. 2020. Prospects of the use of cell therapy to induce immune tolerance. Front. Immunol. 11:792. 10.3389/fimmu.2020.00792 32477335 PMC7235417

[bib90] Watanabe, S., M. Alexander, A.V. Misharin, and G.R.S. Budinger. 2019. The role of macrophages in the resolution of inflammation. J. Clin. Invest. 129:2619–2628. 10.1172/JCI124615 31107246 PMC6597225

[bib91] Wynn, T.A., and K.M. Vannella. 2016. Macrophages in tissue repair, regeneration, and fibrosis. Immunity. 44:450–462. 10.1016/j.immuni.2016.02.015 26982353 PMC4794754

[bib92] Yao, Y., M. Jeyanathan, S. Haddadi, N.G. Barra, M. Vaseghi-Shanjani, D. Damjanovic, R. Lai, S. Afkhami, Y. Chen, A. Dvorkin-Gheva, . 2018. Induction of autonomous memory alveolar macrophages requires T cell help and is critical to trained immunity. Cell. 175:1634–1650.e17. 10.1016/j.cell.2018.09.042 30433869

[bib93] Yevshin, I., R. Sharipov, T. Valeev, A. Kel, and F. Kolpakov. 2017. GTRD: A database of transcription factor binding sites identified by ChIP-seq experiments. Nucleic Acids Res. 45:D61–D67. 10.1093/nar/gkw951 27924024 PMC5210645

[bib94] Yin, C., and B. Heit. 2021. Cellular responses to the efferocytosis of apoptotic cells. Front. Immunol. 12:631714. 10.3389/fimmu.2021.631714 33959122 PMC8093429

[bib95] Yona, S., K.W. Kim, Y. Wolf, A. Mildner, D. Varol, M. Breker, D. Strauss-Ayali, S. Viukov, M. Guilliams, A. Misharin, . 2013. Fate mapping reveals origins and dynamics of monocytes and tissue macrophages under homeostasis. Immunity. 38:79–91. 10.1016/j.immuni.2012.12.001 23273845 PMC3908543

[bib96] Yoshida, K., T. Maekawa, Y. Zhu, C. Renard-Guillet, B. Chatton, K. Inoue, T. Uchiyama, K.i. Ishibashi, T. Yamada, N. Ohno, . 2015. The transcription factor ATF7 mediates lipopolysaccharide-induced epigenetic changes in macrophages involved in innate immunological memory. Nat. Immunol. 16:1034–1043. 10.1038/ni.3257 26322480

[bib97] Yu, G., L.G. Wang, and Q.Y. He. 2015. ChIPseeker: An R/bioconductor package for ChIP peak annotation, comparison and visualization. Bioinformatics. 31:2382–2383. 10.1093/bioinformatics/btv145 25765347

[bib98] Yu, X., A. Buttgereit, I. Lelios, S.G. Utz, D. Cansever, B. Becher, and M. Greter. 2017. The cytokine TGF-β promotes the development and homeostasis of alveolar macrophages. Immunity. 47:903–912.e4. 10.1016/j.immuni.2017.10.007 29126797

[bib99] Yurdagul, A., Jr., A.C. Doran, B. Cai, G. Fredman, and I.A. Tabas. 2017. Mechanisms and consequences of defective efferocytosis in atherosclerosis. Front. Cardiovasc. Med. 4:86. 10.3389/fcvm.2017.00086 29379788 PMC5770804

[bib100] Yurdagul, A., Jr., M. Subramanian, X. Wang, S.B. Crown, O.R. Ilkayeva, L. Darville, G.K. Kolluru, C.C. Rymond, B.D. Gerlach, Z. Zheng, . 2020. Macrophage metabolism of apoptotic cell-derived arginine promotes continual efferocytosis and resolution of injury. Cell Metab. 31:518–533.e10. 10.1016/j.cmet.2020.01.001 32004476 PMC7173557

[bib101] Zhang, J., C. Qu, T. Li, W. Cui, X. Wang, and J. Du. 2019a. Phagocytosis mediated by scavenger receptor class BI promotes macrophage transition during skeletal muscle regeneration. J. Biol. Chem. 294:15672–15685. 10.1074/jbc.RA119.008795 31462534 PMC6816089

[bib102] Zhang, S., S. Weinberg, M. DeBerge, A. Gainullina, M. Schipma, J.M. Kinchen, I. Ben-Sahra, D.R. Gius, L. Yvan-Charvet, N.S. Chandel, . 2019b. Efferocytosis fuels requirements of fatty acid oxidation and the electron transport chain to polarize macrophages for tissue repair. Cell Metab. 29:443–456.e5. 10.1016/j.cmet.2018.12.004 30595481 PMC6471613

[bib103] Zhang, Y., T. Liu, C.A. Meyer, J. Eeckhoute, D.S. Johnson, B.E. Bernstein, C. Nusbaum, R.M. Myers, M. Brown, W. Li, and X.S. Liu. 2008. Model-based analysis of ChIP-seq (MACS). Genome Biol. 9:R137. 10.1186/gb-2008-9-9-r137 18798982 PMC2592715

[bib104] Zhu, B., Y. Wu, S. Huang, R. Zhang, Y.M. Son, C. Li, I.S. Cheon, X. Gao, M. Wang, Y. Chen, . 2021. Uncoupling of macrophage inflammation from self-renewal modulates host recovery from respiratory viral infection. Immunity. 54:1200–1218.e9. 10.1016/j.immuni.2021.04.001 33951416 PMC8192557

