# Supplementary material for: Trained immunity of alveolar macrophages enhances injury resolution via KLF4-MERTK-mediated efferocytosis
Source: J Exp Med. 2023 Aug 24;220(11):e20221388. doi: 10.1084/jem.20221388 (PMC10450795; doi:10.1084/jem.20221388)
Supplement: Table S1 — lists the sources of antibodies used for flow cytometry along with their dilution ratios. [file JEM_20221388_TableS1.docx]

**Table S1. Antibodies used for flow cytometry**

| **Antibodies** | **Source** | **Cat. #** | **Dilution** |
| --- | --- | --- | --- |
| TruStain FcX (anti-mouse CD16/32) Antibody | Biolegend | 101320 | 1:100 |
| APC anti-mouse CD170 (Siglec-F) Antibody | Biolegend | 155507 | 1:200 |
| PE Rat Anti-Mouse Siglec-F | BD Bioscience | 562068 | 1:200 |
| APC anti-mouse CD11c Antibody | Biolegend | 117310 | 1:200 |
| FITC anti-mouse CD11c Antibody | Biolegend | 117306 | 1:200 |
| PE anti-mouse/human CD11b Antibody | Biolegend | 101208 | 1:200 |
| PE/Cy7 anti-mouse CD64 (FcγRI) Antibody | Biolegend | 139313 | 1:200 |
| APC/Fire 750 anti-mouse CD45 Antibody | Biolegend | 103154 | 1:200 |
| Brilliant Violet 421 anti-mouse Ly-6C Antibody | Biolegend | 128031 | 1:200 |
| Brilliant Violet 421 anti-mouse Ly-6G Antibody | Biolegend | 127627 | 1:200 |
| Alexa Fluor 647 anti-mouse IL-10 Antibody | Biolegend | 505016 | 1:100 |
| Brilliant Violet 421 anti-mouse TNF-α Antibody | Biolegend | 506327 | 1:100 |
| Brilliant Violet 421 anti-mouse CD80 Antibody | Biolegend | 104725 | 1:200 |
| FITC anti-mouse CD206 (MMR) Antibody | Biolegend | 141703 | 1:200 |
| Alexa Fluor® 700 anti-mouse CD86 Antibody | Biolegend | 105023 | 1:200 |
| Alexa Fluor® 700 anti-mouse I-A/I-E Antibody | Biolegend | 107621 | 1:200 |
| FITC anti-mouse MERTK (Mer) Antibody | Biolegend | 151503 | 1:100 |
| PE anti-mouse MERTK (Mer) Antibody | Biolegend | 151505 | 1:200 |
| APC anti-BrdU Antibody | Biolegend | 364113 | As recommended |
| FITC Annexin V | Biolegend | 640905 | As recommended |
| Klf4 antibody (FITC) | Biorbyt | orb14085 | 1:100 |
| CD11b Monoclonal Antibody (M1/70), eFluor 450, eBioscience™ | Thermo Fischer | 48-0112-82 | 1:200 |
| Ly-6G/Ly-6C Monoclonal Antibody (RB6-8C5), eFluor 450, eBioscience™ | Thermo Fischer | 48-5931-82 | 1:200 |
| Ki-67 Monoclonal Antibody (SolA15), eFluor 450, eBioscience™ | Thermo Fischer | 48-5698-80 | 1:100 |
| Alexa Fluor 647 Rabbit Anti-Active Caspase-3, BD Bioscience | BD Bioscience | 560626 | As recommended |
