# Supplementary material for: Trained immunity of alveolar macrophages enhances injury resolution via KLF4-MERTK-mediated efferocytosis
Source: J Exp Med. 2023 Aug 24;220(11):e20221388. doi: 10.1084/jem.20221388 (PMC10450795; doi:10.1084/jem.20221388)
Supplement: Table S2 — lists the antibodies used for CyTOF assay in this study as well as their dilution ratios. [file JEM_20221388_TableS2.docx]

**Table S2. Antibodies used for CyTOF (mass cytometry)**

| **Conjugated antibodies** | | | |
| --- | --- | --- | --- |
| **Target** | **Metal tag** | **Company** | **Cat #** |
| CD45 | 89Y | Fluidigm | 3089005B |
| CD64 | 151Eu | Fluidigm | 3151012B |
| CD11b | 154Sm | Fluidigm | 3154006B |
| CD11c | 209Bi | Fluidigm | 3209005B |
| CD206 | 169Tm | Fluidigm | 3169021B |
| CD16/32 | 153Eu | Fluidigm | 3153011B |
| CD86 | 172Yb | Fluidigm | 3172016B |
| CD80 | 171Yb | Fluidigm | 3171008B |
| MHC I | 144Nd | Fluidigm | 3144016B |
| MHCII | 174Yb | Fluidigm | 3174003B |
| CX3CR1 | 164Dy | Fluidigm | 3164023B |
| F/480 | 159Tb | Fluidigm | 3159009B |
| CD169 | 170Er | Fluidigm | 3170018B |
| Ly6G | 141Pr | Fluidigm | 3141008B |
| Ly6c | 150Nd | Fluidigm | 3150010B |
| CD4 | 145Nd | Fluidigm | 3145002B |
| CD8 | 146Nd | Fluidigm | 3146003B |
| CD19 | 149Sm | Fluidigm | 3149002B |
| NK1.1 | 165Ho | Fluidigm | 3165018B |
| Epcam | 166Er | Fluidigm | 3166014B |
| TNFα | 162Dy | Fluidigm | 3162002B |
| IL10 | 158Gd | Fluidigm | 3158002B |
| Active Caspase-3 | 142Nd | Fluidigm | 3142004A |
| **Custom labeled antibodies** | | | |
| Siglec-F | 155Gd | BD Bioscience | 552125 |
| MerTK | 161Dy | R&D Biosystems | AF591 |
| Tim4 | 167Er | R&D Biosystems | MAB2826-100 |
| Marco | 175Lu | R&D Biosystems | MAB2956-100 |
| CD68 | 148Nd | R&D Biosystems | MAB101141-100 |
| Arg1 | 152Sm | R&D Biosystems | AF5868 |
| Brdu | 156Gd | R&D Biosystems | MAB7225 |
| CCR2 | 147Sm | R&D Biosystems | MAB55381R-100 |
| Lyve1 | 176Yb | R&D Biosystems | MAB2125-100 |
| CD163 | 168Er | Thermo Fischer | 16646-1-AP |
| CD103 | 173Yb | R and D Biosystem | AF1990 |
| CD31 | 143Nd | Biolegend | 102425 |
| V-ATPase | 163Dy | Thermo Fischer | PA5-29899 |
| Anti-NOX2 | 160Gd | Thermo Fischer | PA5-76034 |
| CD24 | 116Cd | Biolegend | 101829 |
