# Supplementary material for: Trained immunity of alveolar macrophages enhances injury resolution via KLF4-MERTK-mediated efferocytosis
Source: J Exp Med. 2023 Aug 24;220(11):e20221388. doi: 10.1084/jem.20221388 (PMC10450795; doi:10.1084/jem.20221388)
Supplement: Table S3 — lists the sequences for primers used in qPCR, chromatin accessibility assay, MSP-qPCR, and ChIP-qPCR. [file JEM_20221388_TableS3.docx]

**Table S3. Primer sequences used in this study**

| Gene | Forward | Reverse | Use for |
| --- | --- | --- | --- |
| MERTK promoter primer set-1 | TGACCCAAGTTCCATTCTGC | GGAAGGGCAAAGAGAGCAAA | ChIP-qPCR |
| MERTK promoter primer set-2 | CCCTCCTTAGCTCTGTTCCA | GCAGAATGGAACTTGGGTCA | ChIP-qPCR |
| MERTK promoter primer set-3 | CCAGAGATCATCCTCCCGAT | TGGAACAGAGCTAAGGAGGG | ChIP-qPCR |
| MERTK promoter primer set-4 | CTCTTGCACTCCGTTTCAGT | GCATAAGAGGTCCTAGCAGC | ChIP-qPCR |
| KLF4 promoter | GACTTTCGAGCCCAGGGAAC | GTTCCCTGGGCTCGAAAGTC | Chromatin accessibility assay |
| KLF4 promoter Methylated primer | TGGTCGTTTAGAGGATTTCGA | CAAACAACTAACGAACTAAAACCGT | MSP-qPCR |
| KLF4 promoter Unmethylated primer | GTGGTTGTTTAGAGGATTTTGA | ACAACTAACAAACTAAAACCATA | MSP-qPCR |
| PPIA | GGCAAATGCTGGACCAAACAC | TTCCTGGACCCAAAACGCTC | qPCR |
| MERTK | GTGGCAGTGAAGACCATGAAGTTG | ATGACTCCCTATCCCGGAGTTC | qPCR |
| Bax | CTACAGGGTTTCATCCAG | CCAGTTCATCTCCAATTCG | qPCR |
| MCL1 | AGCCTTGTGAGTGCAATAGG | AAAATCCTGGGCAGCTTCAA | qPCR |
| IL10 | GCTCTTACTGACTGGCATGAG | CGCAGCTCTAGGAGCATGTG | qPCR |
| TNFα | CCCTCACACTCAGATCATCTTCT | GCTACGACGTGGGCTACAG | qPCR |
| KLF4 | GTCAAGTTCCCAGCAAGTCA | AGACCCCATCTGTTCTGTGA | qPCR |
| BAX | CTACAGGGTTTCATCCAG | CCAGTTCATCTCCAATTCG | qPCR |
| MCL1 | AGCCTTGTGAGTGCAATAGG | AAAATCCTGGGCAGCTTCAA | qPCR |
| DUSP1 | AGGATATGCTTGACGCCTTG | CCACAGGGATGCTCTTGTAC | qPCR |
| SOCS3 | ACCTTCAGCTCCAAAAGCGAGTAC | CGCTCCAGTAGAATCCGCTCTC | qPCR |
| CLEC7a | ATCAAGCCTACTGAACCCCAAG | CTTCTCCCTGTGTCTGGTGTAC | qPCR |
| CD16 | CCTAGTGATGTGCCTCCTGTTT | GCCTGCTTGTAAGTTGCTTTCA | qPCR |
| TYRO3 | GAACTTCATCCACCGAGACC | TGACGATAATAGTCCCCGCT | qPCR |
| AXL | TGAGCCAACCGTGGAAAGAG | AGGCCACCTTATGCCGATCTA | qPCR |
| SCARB1 | ATTCCCACGTATCGCTTCAC | ACAGCTTCTGACAACACAGG | qPCR |
| CD36 | TGGCCTTACTTGGGATTGG | CCAGTGTATATGTAGGCTCATCCA- | qPCR |
| TIM4 | GACAGCGTCCTCAATGACAT | ATACAACCAGACAGGACCCA | qPCR |
| TIM3 | GACCCTCCATAATAACAA | TAATAAGGCTCAAACTCG | qPCR |
| FCGR2B | CTCACGGACTTTGTGCCATA | CTTCCTTGAGCACCTGGATC | qPCR |
| OLR1 | GTTCCCTGCTGCTATGACTC | TAACATCTGCCCTTCCAGGA | qPCR |
| MARCO | GATCCAGTGCCCAAGAAGAG | TAGTGATCCATTGCCACAGC | qPCR |
