# Supplementary material for: Trained immunity of alveolar macrophages enhances injury resolution via KLF4-MERTK-mediated efferocytosis
Source: J Exp Med. 2023 Aug 24;220(11):e20221388. doi: 10.1084/jem.20221388 (PMC10450795; doi:10.1084/jem.20221388)
Supplement: Table S4 — lists differentially expressed genes of naïve vs. trained AM shown in the Fig. 1 H heatmap. [file JEM_20221388_TableS4.docx]

**Table S4. Differentially expressed gene list of naïve vs. trained AM shown in the Fig. 1 H heatmap**

| **Gene name** | **Gene ID** |
| --- | --- |
| Myo10 | ENSMUSG00000022272 |
| Pdgfb | ENSMUSG00000000489 |
| Adora2b | ENSMUSG00000018500 |
| Il1b | ENSMUSG00000027398 |
| Apoc1 | ENSMUSG00000040564 |
| C2 | ENSMUSG00000024371 |
| Sphk1 | ENSMUSG00000061878 |
| Msr1 | ENSMUSG00000025044 |
| Cxcl1 | ENSMUSG00000029380 |
| Tnip3 | ENSMUSG00000044162 |
| Rasgrp1 | ENSMUSG00000027347 |
| Fnbp1l | ENSMUSG00000039735 |
| Cd177 | ENSMUSG00000052212 |
| Traf1 | ENSMUSG00000026875 |
| Sash1 | ENSMUSG00000015305 |
| Sdc4 | ENSMUSG00000017009 |
| Anpep | ENSMUSG00000039062 |
| Adora2a | ENSMUSG00000020178 |
| Pf4 | ENSMUSG00000029373 |
| Ptafr | ENSMUSG00000056529 |
| Ccr5 | ENSMUSG00000079227 |
| Met | ENSMUSG00000009376 |
| Stx11 | ENSMUSG00000039232 |
| Tnip1 | ENSMUSG00000020400 |
| Acer3 | ENSMUSG00000030760 |
| Il7 | ENSMUSG00000040329 |
| Plpp3 | ENSMUSG00000028517 |
| Ereg | ENSMUSG00000029377 |
| Nlrc5 | ENSMUSG00000074151 |
| Arhgap10 | ENSMUSG00000037148 |
| Mmp14 | ENSMUSG00000000957 |
| Ptgs2 | ENSMUSG00000032487 |
| Slc7a11 | ENSMUSG00000027737 |
| Inhba | ENSMUSG00000041324 |
| Tns3 | ENSMUSG00000020422 |
| Il7r | ENSMUSG00000003882 |
| Socs3 | ENSMUSG00000053113 |
| Acod1 | ENSMUSG00000022126 |
| Procr | ENSMUSG00000027611 |
| Ptges | ENSMUSG00000050737 |
| Pmp22 | ENSMUSG00000018217 |
| Nrg1 | ENSMUSG00000062991 |
| Il10 | ENSMUSG00000016529 |
| Serpinb2 | ENSMUSG00000062345 |
| Pdpn | ENSMUSG00000028583 |
| Ebi3 | ENSMUSG00000003206 |
| Dcbld2 | ENSMUSG00000035107 |
| Cxcl11 | ENSMUSG00000060183 |
| Mcoln2 | ENSMUSG00000011008 |
| Ass1 | ENSMUSG00000076441 |
| Gbp3 | ENSMUSG00000028268 |
| Oas2 | ENSMUSG00000032690 |
| Cdc42ep2 | ENSMUSG00000045664 |
| Cxcl3 | ENSMUSG00000029379 |
| Gng12 | ENSMUSG00000036402 |
| Clec4e | ENSMUSG00000030142 |
